# Angiosperm flowers reached their highest morphological diversity early in their evolutionary history

**DOI:** 10.1111/nph.19389

**Published:** 2023-11-29

**Authors:** Andrea M. López‐Martínez, Susana Magallón, Maria von Balthazar, Jürg Schönenberger, Hervé Sauquet, Marion Chartier

**Affiliations:** ^1^ Posgrado en Ciencias Biológicas, Instituto de Biología Universidad Nacional Autónoma de México, 3er Circuito de Ciudad Universitaria Coyoacán Ciudad de México 04510 Mexico; ^2^ Departamento de Botánica, Instituto de Biología Universidad Nacional Autónoma de México, 3er Circuito de Ciudad Universitaria Coyoacán Ciudad de México 04510 Mexico; ^3^ Department of Botany and Biodiversity Research University of Vienna Rennweg 14 Vienna A‐1030 Austria; ^4^ National Herbarium of New South Wales (NSW) Royal Botanic Gardens and Domain Trust Sydney NSW 2000 Australia; ^5^ Evolution and Ecology Research Centre, School of Biological, Earth and Environmental Sciences University of New South Wales, Biological Sciences North (D26) Sydney NSW 2052 Australia

**Keywords:** ancestral state reconstruction (ASR), categorical morphospace, Cretaceous, disparity through time, floral evolution, Paleogene

## Abstract

- Flowers are the complex and highly diverse reproductive structures of angiosperms. Because of their role in sexual reproduction, the evolution of flowers is tightly linked to angiosperm speciation and diversification. Accordingly, the quantification of floral morphological diversity (disparity) among angiosperm subgroups and through time may give important insights into the evolutionary history of angiosperms as a whole.
- Based on a comprehensive dataset focusing on 30 characters describing floral structure across angiosperms, we used 1201 extant and 121 fossil flowers to measure floral disparity and explore patterns of floral evolution through time and across lineages.
- We found that angiosperms reached their highest floral disparity in the Early Cretaceous. However, decreasing disparity toward the present likely has not precluded the innovation of other complex traits at other morphological levels, which likely played a key role in the outstanding angiosperm species richness.
- Angiosperms occupy specific regions of the theoretical morphospace, indicating that only a portion of the possible floral trait combinations is observed in nature. The ANA grade, the magnoliids, and the early‐eudicot grade occupy large areas of the morphospace (higher disparity), whereas nested groups occupy narrower regions (lower disparity).

## Introduction

Disparity, or morphological diversity, is a quantification of the morphological variation displayed by a group of organisms (Foote, 1992a, 1993a, 1995, 1997; Wills *et al*., 1994; Ciampaglio *et al*., 2001; Erwin, 2007; Guillerme *et al*., 2020; Hopkins & Gerber, 2021). Measures of disparity are based on the multivariate description of morphological traits and are often coupled with the ordination of morphospaces that describe and relate the phenotypic configurations of organisms (Foote, 1994; Mitteroecker & Huttegger, 2009; Smith & Donoghue, 2022). The variation of disparity and morphospace occupation within and among clades, populations, fossil assemblages, or time periods, may provide useful information on a lineage's morphological evolution (Foote, 1994; Benton, 2015), on the evolutionary constraints shaping phenotypes (Ciampaglio, 2002; Allen *et al*., 2008), on the ecology and natural selection exerted on populations (Benitez‐Vieyra *et al*., 2010), and on the effect of mass extinction on lineages (Brusatte *et al*., 2008a; Friedman, 2010; Korn *et al*., 2013; Puttick *et al*., 2020). In particular, studying disparity through time (DTT) using fossils gives insight into the evolutionary dynamics of lineages and the general patterns of morphological evolution on Earth (McGhee, 1991; Foote, 1993b; Hughes *et al*., 2013; Oyston *et al*., 2016).

Disparity has generally evolved nonuniformly and independently from species richness throughout the history of lineages (Foote, 1994, 1995; Slater *et al*., 2010; Benton *et al*., 2014; Moon & Stubbs, 2020; Coombs *et al*., 2022). Two main trends in the variation of DTT have been observed with respect to when maximal disparity was reached. Most lineages reached maximal disparity early in their evolution (‘early‐disparity’, ‘bottom‐heavy’ distribution: Gould *et al*., 1987; Gould, 1989; Hughes *et al*., 2013). These early‐disparity trends result from abrupt and wide morphospace exploration possibly due to the rapid colonization and exploitation of new environments, or of an ecological niche that was left vacant after an extinction event (Foote, 1994; Benton *et al*., 2014; Benton, 2015; Leslie *et al*., 2021). Alternatively, new morphologies might accumulate slowly as clades diversify so that disparity might reach its maximal levels late in a clade's evolution (‘late‐disparity’, or ‘top‐heavy’ distribution; Gould *et al*., 1987; Prentice *et al*., 2011; Hughes *et al*., 2013; Puttick *et al*., 2020). Although disparity variation and morphospace occupation through time have been described for various animal clades (e.g. Foote, 1991 for blastoids; Foote, 1994, 1999 for crinoids; Fortey *et al*., 1996 for several animal groups; Brusatte *et al*., 2008b for dinosaurs; Bapst *et al*., 2012 for graptoloids; Hill *et al*., 2018 for fishes), only few studies have quantified DTT in angiosperms (e.g. Lupia, 1999; Jardine *et al*., 2022 for pollen; Oyston *et al*., 2016; Clark *et al*., 2023 for general morphology; Martínez‐Cabrera *et al*., 2017 for wood properties).

Flowers are a relatively recent evolutionary innovation and yet they have evolved remarkable morphological and functional diversity (Endress, 2011; Sauquet *et al*., 2017, 2022). Because floral morphology is directly linked to angiosperm reproductive success, flowers are under strong selective pressures and studying their morphological diversity is crucial to understand trends of lineage divergence and evolutionary success (Harder & Barrett, 2006; Endress, 2011; van der Niet & Johnson, 2012; Chartier *et al*., 2014; Sauquet & Magallón, 2018). The diversity of flowers has been extensively discussed qualitatively, based on the development and comparative morphology of various extant groups (Endress & Igersheim, 1997, 1999, 2000; Matthews & Endress, 2002, 2005, 2006; Schönenberger & von Balthazar, 2006; Endress, 2010, 2011; Schönenberger *et al*., 2010). Based on these studies, it is for example widely recognized that floral organization (Bauplan) is relatively labile among the ANA grade lineages, magnoliids, and early‐diverging eudicots, all of which exhibit, for instance, high variability in merism (organ number) and phyllotaxis (the arrangement of floral organs). By contrast, floral organization is more stable in large clades such as the monocots with almost exclusively trimerous, whorled flowers, or in Pentapetalae with mostly pentamerous, whorled flowers (Endress, 2010, 2011; Sauquet *et al*., 2017).

The first study using a morphospace approach to analyze floral morphology across angiosperms is the theoretical morphospace created by Stebbins (1951). A theoretical morphospace allows describing the entire spectrum of theoretically possible morphologies for a group of organisms and, therefore, determining limits in the evolution of biological forms and identifying areas of possible high vs low fitness (Raup & Michelson, 1965; Raup, 1967; McGhee, 1991, 2015; Avena‐Koenigsberger *et al*., 2015). Using this approach, Stebbins determined a set of floral trait combinations that were unlikely to occur in angiosperms and others that were particularly successful (common; Stebbins, 1951). A later quantitative re‐analysis of Stebbins' dataset confirmed the qualitative observations of plant morphologists that the highest floral disparity is present in the ANA grade, magnoliids, and early‐diverging eudicots, whereas the lowest disparity was found in the nested clades such as lamiids, campanulids, and malvids (Chartier *et al*., 2014). The shortcoming of these two studies is that Stebbins' original dataset only included eight binary floral characters and was coded at the family level based on a now largely outdated classification of angiosperms. The study of floral evolution and diversity during the last decades and the reconstruction of ancestral floral morphologies (e.g. Sauquet *et al*., 2017) allow us to study disparity and morphological trajectories using unprecedently large datasets through time at the level of angiosperms as a whole. In addition, the recent description of numerous fossil flowers provides information about past floral diversification (e.g. Crepet *et al*., 1991; Endress, 2006; Endress & Doyle, 2009; Friis *et al*., 2010, 2011; Doyle & Endress, 2014) and makes it possible to study DTT in angiosperms. Most fossil flowers stem from Cretaceous sediments and include predominantly three‐dimensionally, well‐preserved, charcoalified mesofossils (e.g. Schönenberger & Friis, 2001; Gandolfo *et al*., 2004; Friis & Pedersen, 2011, 2012) while a few are preserved as permineralizations (e.g. Atkinson *et al*., 2015), impressions/compressions (e.g. Dilcher & Crane, 1984; Mohr & Eklund, 2004), and amber inclusions (e.g. Gandolfo *et al*., 2018).

In this study, we use an extensive morphological dataset describing characters at the level of floral organization (Bauplan; *sensu* Endress, 1994). We include most of the best‐preserved fossil flowers known and a broad representation of living species to investigate the large‐scale temporal and phylogenetic patterns in the evolution of angiosperm flowers. First, we quantify and describe the changes in disparity and morphospace occupation through geologic time. Second, we explore the position of living species (split into 11 taxonomic groups) in a theoretical floral morphospace, and test for the correlation between disparity, clade age, and clade species richness in these groups.

## Materials and Methods

All analyses were performed in R v.4.1.2 (R Core Team, 2022; Supporting Information Dataset S1). In the following, function names and packages are referred to as *function name* {package name}.

### Analyses overview

We performed three different analyses. For each analysis, a distance matrix (see Distance matrices in the Materials and Methods section) containing the taxa of interest (see Morphological dataset in the Materials and Methods section) was computed and then used to calculate disparity (see Estimating disparity in the Materials and Methods section), then an ordination was generated to visualize the morphospace (see Ordination in the Materials and Methods section).

#### Disparity through time

We compared disparity and morphospace occupation among four stratigraphic time bins: the Early Cretaceous (145–100.5 Ma), the Late Cretaceous (100.5–66 Ma), the Paleogene (66–23.03 Ma; Cohen *et al*., 2013), and living species (present time). Detailed morphological descriptions were only available for two species from the Neogene (23.03–5.333 Ma; Castañeda‐Posadas & Cevallos‐Ferriz, 2007; Hernández‐Damián *et al*., 2018; Cohen *et al*., 2013); Neogene fossils were thus excluded from the statistical analyses.

#### Angiosperm trajectory in the morphospace

We placed ancestral flowers reconstructed for the crown nodes of 15 key angiosperm clades (see Ancestral state reconstructions in the Materials and Methods section) in the floral morphospace and compared their position with that of living and fossil angiosperms.

#### Disparity among living angiosperms

We compared disparity and morphospace occupation among living angiosperms split into 11 groups (including clades and grades) and tested for the correlation among disparity, age, and species richness for these groups. We split species into the following six clades: magnoliids, commelinids, fabids, malvids, campanulids, and lamiids (*sensu* APG IV, 2016), and five grades: ANA (Amborellales, Nymphaeales, and Austrobaileyales), other monocots (all monocot lineages except commelinids), other eudicots (Ranunculales, Proteales, Trochodendrales, and Buxales), other superrosids (Saxifragales and Vitales), and other superasterids (Santalales, Berberopsidales, Caryophyllales, Cornales, and Ericales; Dataset S2). The orders Chloranthales, Ceratophyllales, Gunnerales, and Dilleniales were excluded from the statistical analyses as they contained only two to four species in our sampling and because they cannot be assigned to any previously specified group.

### Morphological dataset

The dataset includes 30 categorical floral characters scored for 1201 living angiosperm species (36 030 matrix cells) sampled from all currently accepted families from APG IV (2016), as well as 121 fossil flowers (3630 matrix cells), which represented most of the best‐preserved fossil flowers described to date. The final data matrix used in analyses and the complete list of fossils and information on their stratigraphic age, mode of preservation, locality, and corresponding references are available in Dataset S2.

Morphological data and references were recorded in the PROTEUS database (Sauquet, 2019). One character describes the whole flower, eight describe the perianth, 11 the androecium, eight the gynoecium, and two the pollen (Sauquet *et al*., 2017; Schönenberger *et al*., 2020). Data for the 1201 living species were previously scored by López‐Martínez *et al*. (2023). Data for 24 of the 121 fossil species were obtained from Schönenberger *et al*. (2020) and López‐Martínez *et al*. (2023), and 97 fossils were additionally scored for this study. A complete extraction of PROTEUS data for the fossil dataset (2442 data records linked to 125 explicit references) is provided as Dataset S3 and the dataset used here is provided as Dataset S2.

The proportion of missing data, including inapplicable characters, is 32% (11 573 matrix cells) for the living species (Fig. S1) and 36% (1305 matrix cells) for the fossils (Fig. S2). Additionally, there were 3% (1310 matrix cells) polymorphic entries for the living species and 2% (74 matrix cells) for the fossils. As most of our analyses do not support polymorphic data, we randomly sampled one of the polymorphic states (with equal probabilities) for each polymorphic entry before each analysis following Chartier *et al*. (2017). Our results did not change among these analyses.

### Theoretical combinations

To visualize the distribution of achieved morphologies among possible ones in the ordinations, we added a background of theoretical combinations to the combinations displayed by the sampled angiosperm species (empirical data). Such an approach is helpful for categorical data like ours, where the nature of the space is discrete rather than continuous (Gerber, 2019). The theoretical morphospace contains all combinations of traits that can be obtained from the study character set. In our data, the total number of possible combinations is the product of the number of states for each character: 2^17^ × 3^8^ × 4^3^ × 5^2^ = 1.38 × 10^12^. To display a subset of the theoretical morphospace in the ordinations, we randomly sampled 2000 of these theoretical combinations without replacement each time we created an ordination. We did not sample theoretical combinations containing inapplicable or impossible combinations of character states (e.g. perianth merism in flowers without perianth); in these cases, the inapplicable character state was treated as missing data. Inapplicable and impossible combinations are listed in Methods S1.

### Distance matrices

For each analysis, a distance matrix was computed by calculating the *mean character difference* (here noted *D*) for each pair of taxa following Sneath & Sokal (1973 p. 135) and Foote (1999). The mean character difference is a version of the Gower index, suited for datasets like ours containing categorical ordered and categorical unordered characters. It ranges from zero (no difference) to 1 (largest difference in the dataset). Its calculation is detailed in Methods S2.

### Estimating disparity

Disparity was estimated for each group (clade, grade, or pool of species belonging to the same time bin) based on four metrics capturing different aspects of morphospace occupation:

The mean pairwise difference (here *meanD*) reflects the density of taxa within a group; it is the *mean character difference* (*D*, stored in the distance matrix) averaged among each pair of taxa (Foote, 1999). This metric is moderately sensitive to sample size (Ciampaglio *et al*., 2001), and since the data present large differences in sample size in some instances (e.g. 22 species in the Paleogene vs 1201 extant angiosperms), we additionally rarefied it. For the rarefaction analysis, *n* species were randomly sampled without replacement in each group (with *n* the size of the smallest group minus one); then, the distance matrix was computed and *meanD* calculated. This was repeated 1000 times.

The range (here noted *R*) gives information about the size of a group in the morphospace (Foote, 1992b); we calculated it as the maximum pairwise distance (*maxD*) in a group. The range is sensitive to sample size (Ciampaglio *et al*., 2001), and we thus rarefied it as described above (we only compared rarefied values).

*MeanD*, rarefied *meanD*, and rarefied *R* were compared among groups with Kruskal–Wallis tests for nonparametric data (Kruskal & Wallis, 1952) using the functions *kruskal* {agricolae} (de Mendiburu & Yaseen, 2020) and *kruskalmc* {pgirmess} (Giraudoux *et al*., 2018).

We also computed a measure of the contribution of a group to total disparity (here noted *Ddelta*; Foote, 1993a). We measured *Ddelta* for group *i* (*Ddelta*_*i*_), as the difference between *meanD* for the dataset without group *i*, and *meanD* for the total dataset (*Dtot*). When using *meanD* as an estimate of disparity, a positive value of *Ddelta*_*i*_ indicates that adding group *i* contributed to an increase in *Dtot*, while a negative value of *Ddelta*_*i*_ indicates that adding group *i* contributed to a decrease in *Dtot*. The magnitude of this contribution is proportional to the absolute value of *Ddelta*_*i*_.

Finally, to detect the living species with the most distinct floral combinations, we estimated the divergence of each species from the average morphology (we called it eccentricity following Oyston *et al*., 2015) by averaging *D* for each living species in the dataset. This was done by averaging each line of the distance matrix. Species with the highest eccentricity are placed at the edge of the occupied morphospace ordination.

### Ordination

We visualized morphospaces with nonmetric multidimensional scaling (nMDS) using the function *metaMDS* {vegan} (Oksanen *et al*., 2020), setting the number of dimensions (*k*) to two and the maximum number of random starts (trymax) to 20 (default value). For each group (time bin or taxonomic group), the position of the centroid was added to the graphs by averaging the coordinates of each point for each axis. Floral morphologies were compared among groups with a nonparametric multivariate analysis of variance (npMANOVA) with the function *adonis2* {vegan} (Oksanen *et al*., 2020). Post hoc tests consisted of pairwise npMANOVAs with a Bonferroni correction.

### Correlations

For living species, we investigated the correlations between disparity (*meanD*) and species richness of clades/grades (extracted from the World Flora Online; WFO, 2023), between *meanD* and clade age (crown age for six clades from Ramírez‐Barahona *et al*., 2020), and between *meanD* and *Ddelta*. Finally, we verified that sample size was positively correlated with species richness. We used Pearson correlation tests using the function *cor.test* {vegan} (Oksanen *et al*., 2020).

### Ancestral state reconstructions

We employed maximum likelihood (ML) and stochastic character mapping (SM) methods to reconstruct ancestral states for each floral trait at 15 angiosperm key nodes corresponding to broadly recognized major angiosperm clades (*sensu* Cantino *et al*., 2007; APG IV, 2016). All ancestral state reconstructions (ASR) were conducted using the maximum clade credibility dated tree from the relaxed calibration complete (RC‐complete) strategy from Ramírez‐Barahona *et al*. (2020). Maximum likelihood reconstructions were performed implementing the equal rates (ER) and all rates different (ARD) models of discrete morphological evolution with the function *rayDISC* {corHMM} (Beaulieu *et al*., 2013; Dataset S4). We subsequently compared model fit based on the Akaike information criterion. SM reconstructions were then conducted using the best‐fitted model obtained with ML. For SM analyses, we first dropped the tips with missing data, nonapplicable, and polymorphic data from the tree using the function *drop.tip* {phytools} (Revell, 2012). We then inferred ancestral states using 500 simulations along the tree with the function *make.simmap* {phytools} (Revell, 2012) and summarized with the function *describe.simmap* {phytools} (Revell, 2012). Dropped tips were removed from the trees showing ASR with SM (Dataset S5). To display ASR in the morphospace, we included the most probable combinations of ancestral states for each node in the dataset before computing the distance matrix, according to Gerber (2019).

## Results

### Morphospace and disparity through time

**Fig. 1:**
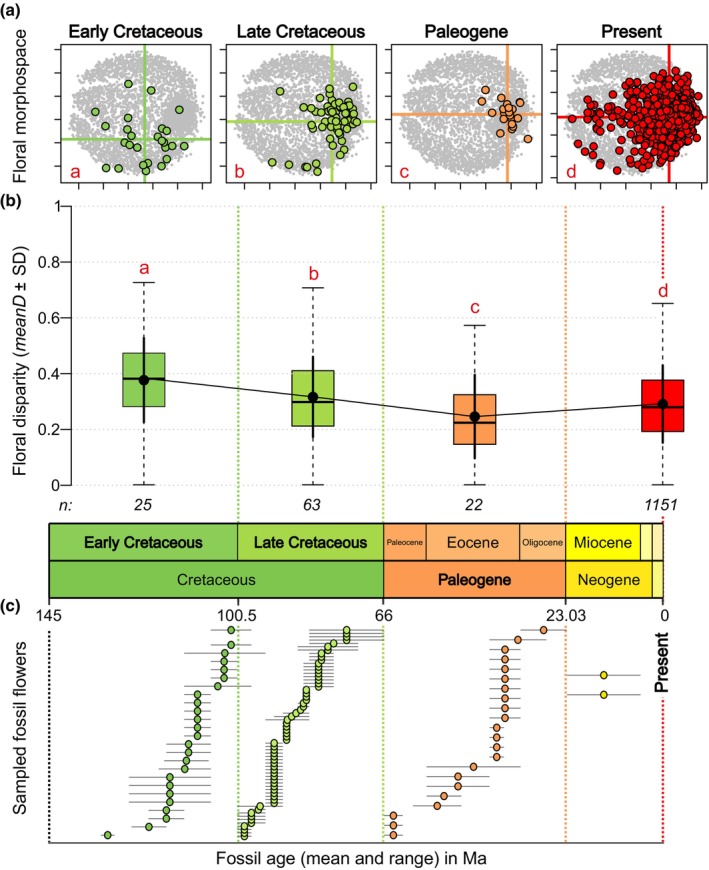
Evolution of the floral morphospace and floral disparity through time for angiosperms. (a) Evolution of morphospace occupation through time (nMDS stress value = 0.34; Shepard plot: nonmetric fit *r* ^2^ = 0.88, linear fit *r* ^2^ = 0.39). Colored lines indicate the position of the centroid for each group. In (b), black dots, disparity (*meanD*) for each time bin. SD, standard deviation (black error bars). Boxplots, distribution of *D* for each group. *n*, sample sizes after computing the distance matrix. In (a) and (b), red letters indicate *post hoc* test results, groups with a different letter significantly differ from each other. (c) Stratigraphic age ranges for each fossil flower. The position of Neogene fossils in the morphospace is presented in Supporting Information Fig. S4.

Fossil floral disparity significantly differed among the four time‐bins (Kruskal–Wallis test: χ^2^ = 207.29, df = 3, *P* = < 2.2e^−16^; Fig. 1b). Angiosperm flowers showed the highest disparity in the Early Cretaceous (*meanD* = 0.372 ± SD 0.14) followed by a decrease toward the Late Cretaceous (*meanD* = 0.316 ± 0.143) and the Paleogene (*meanD* = 0.241 ± 0.13). Disparity for living species reached values intermediate between Late Cretaceous and Paleogene (*meanD* = 0.29 ± 0.137; Fig. 1b). This pattern was confirmed by the rarefaction analyses of *meanD* (Fig. S3a) and of *R*, with the exception of the Paleogene group showing a range as high as in the Early Cretaceous (Fig. S3b). Note that this pattern applies to angiosperms as a whole and that disparity within subgroups might vary differently (Fig. S3c–e).

**Fig. 2:**
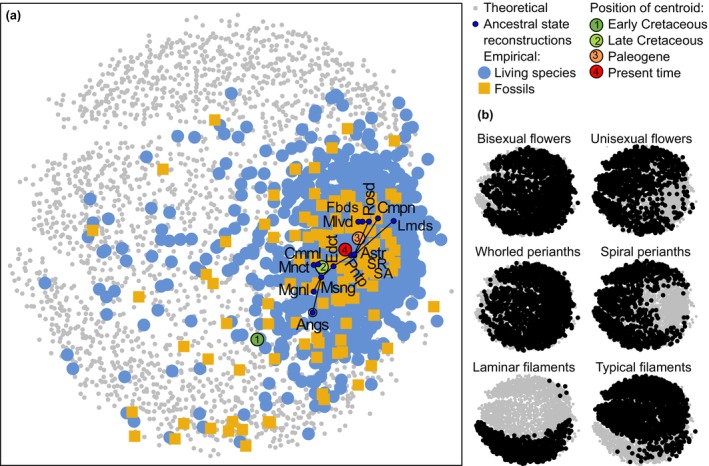
Total floral morphospace of angiosperms (nMDS stress value = 0.34; Shepard plot: nonmetric fit *r* ^2^ = 0.88, linear fit *r* ^2^ = 0.39), including 1151 living species (light blue dots, *meanD* = 0.29, *R* = 1), 113 fossils (yellow squares, *meanD* = 0.34, *R* = 1), 15 ancestral state reconstructions (ASR; stochastic character mapping (SM) analyses, navy blue dots, *meanD* = 0.16, *R* = 0.35) and 2000 theoretical combinations (gray dots), displayed in (a). Angs, angiosperms; Astr, asterids; Cmml, commelinids; Cmpn, campanulids; Edct, eudicots; Fabd, fabids; Lamd, lamiids; Mgnl, magnoliids; Mlvd, malvids; Mnct, monocots; Msng, mesangiosperms; Pntp, Pentapetalae; Rosd, rosids; SA, superasterids; SR, superrosids. Links among ancestors illustrate phylogenetic relationships. In (b), distribution of some selected character states (black dots) in the same space (gray dots; see also Supporting Information Fig. S5). The values for *meanD* and *R* given here are not rarefied. Note the round shape of the theoretical space ordination, similar to the ordinations of other theoretical spaces of categorical nature (e.g. Gerber, 2019).

The morphospace area occupied by fossil floral morphologies significantly varied in time (npMANOVA: *F* = 21.583 *P* < 1e^−4^; Fig. 1a), with the position of the centroid showing a displacement from the middle bottom part to the right middle side of the morphospace (Figs 1a, 2). Although all reconstructed ancestors analyzed here dated from the Jurassic to the Early Cretaceous (Table S1), they followed the same trajectory as the position of the centroid for fossils through time and were mostly restricted to the area densely occupied by living species (Fig. 2).

### Morphospace and disparity for living angiosperms

Ninety‐five percent of living species occupied a restricted area of the morphospace ordination (as a rough indication, the corresponding convex hull covered 36% of the total morphospace; Fig. S6) and the remaining 5% of the living species with the highest eccentricity were distributed in otherwise largely empty areas of the theoretical morphospace (the corresponding convex hull covered 60% of the total morphospace; Fig. S6).

In our dataset, 45% of living species had bisexual flowers with one whorl of sepals and one whorl of petals, a total of 6–10 perianth parts, together with a differentiated style (Fig. S7). By contrast, the floral traits characterizing the empty area of the theoretical morphospace (traits more rarely observed in living species) included, in combination or not: dimerous perianths and androecia with more than two whorls each, diaperturate and inaperturate pollen grains, the absence of a perianth, spiral perianth and androecium phyllotaxis, flap‐valvate and H‐valvate anther dehiscence, fused ovaries, undifferentiated styles, as well as free‐central and laminar placentation (Fig. S7). The five species with the highest eccentricity were as follows: *Eupomatia bennetii* F.Muell. and *E. laurina* R.Br. (magnoliids), *Cyclanthus bipartitus* Poit. ex A.Rich. (other monocots grade), *Galbulimima belgraveana* (F.Muell.) Sprague (magnoliids), and *Sarcandra chloranthoides* Gardner (Chloranthales; Fig. S6).

**Fig. 3:**
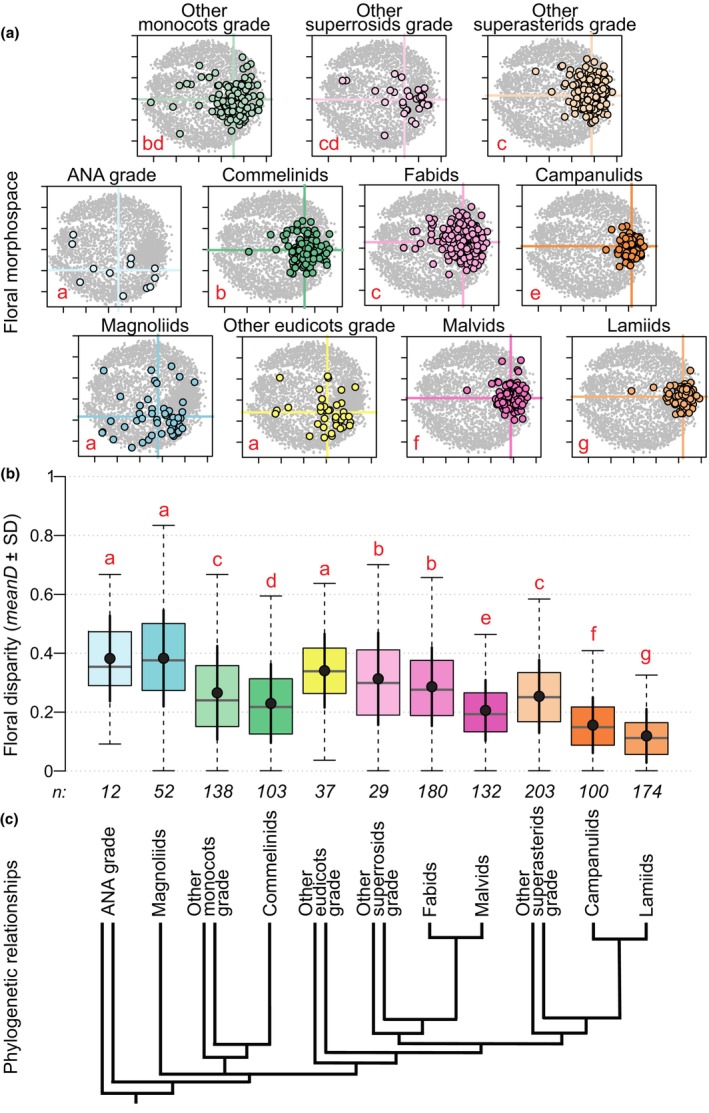
Floral morphospace and disparity for living angiosperms. (a) Morphospace occupation for 11 angiosperm groups (nMDS stress value = 0.34; Shepard plot: nonmetric fit *r* ^2^ = 0.88, linear fit *r* ^2^ = 0.38). In each plot, colored dots = species belonging to the group of interest, gray dots = 2000 theoretical combinations and the remaining angiosperm species; the intersection of the colored lines indicates the position of the centroid of each group. (b) Disparity (*meanD*, black dots) ± standard deviation (black vertical plain lines). Boxplots show the distribution of pairwise distances (*D*) for each group, numbers below each boxplot correspond to sample size after computing the distance matrix. In (a) and (b), red letters indicate *post hoc* test results, groups with a different letter significantly differ from each other. (c) Phylogenetic relationships among major angiosperm lineages following Ramírez‐Barahona *et al*. (2020). The position of Chloranthales, Ceratophyllales, Gunnerales, and Dilleniales in this morphospace is presented in Supporting Information Fig. S9.

**Fig. 4:**
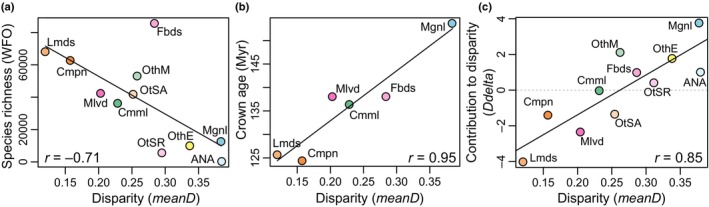
Correlations between disparity (*meanD*) and (a) species richness, (b) crown node age for six clades, (c) contribution of a group to total disparity (*Ddelta*). Black lines: linear regressions. *r* = Pearson's product‐moment correlation coefficient. ANA, ANA grade; Cmml, commelinids; Cmpn, campanulids; Fbds, fabids; Lmds, lamiids; Mgnl, magnoliids; Mlvd, malvids; Myr, million years ago; OthE, the other eudicots grade; OthM, the other monocots grade; OtSA, the other superasterids grade; OtSR, the other superrosids grade; WFO, World Flora Online (WFO, 2023).

Disparity differed significantly among angiosperm groups (Kruskal–Wallis test: χ^2^ = 18 392, df = 10, *P* = < 2.2e^−16^; Fig. 3b; Table S2). Floral disparity was highest within magnoliids (*meanD* = 0.378 ± SD 0.16), the ANA grade (*meanD* = 0.370 ± 0.14), and the other eudicots grade (*meanD* = 0.334 ± 0.16). These three groups also contributed the most to total disparity (Fig. 4c). The lowest disparity was found in nested clades within the Pentapetalae clade, such as campanulids (*meanD* = 0.158 ± 0.09) and lamiids (*meanD* = 0.12 ± 0.08; Fig. 3b). These two groups contributed the least to total disparity (Fig. 4c). Rarefied *meanD* and rarefied *R* showed similar results (Fig. S8).

The position of living clades and grades in the space varied significantly (npMANOVA: *F* = 41.78, *r* ^2^ = 0.23, *P* < 1e^−04^; Fig. 3a), meaning that each group presented specific combinations of floral traits. Exceptions (nonsignificantly different groups) were as follows: (1) the ANA grade, magnoliids, and the other eudicots grade, (2) commelinids and the other monocots grade, (3) fabids, the other superasterids grade, and the other superrosids grade, (4) the other monocots grade and the other superrosids grade (Fig. 3a).

### Correlations

Disparity significantly decreased with species richness (Pearson: *t* = −3.01, df = 9, *P* = 0.015; Fig. 4a), with the exception of fabids, which showed medium disparity (*meanD* = 0.29) and a large number of species (85621). Disparity significantly increased with crown node age (*t* = 5.80, df = 4, *P* = 0.004; Fig. 4b) and with the contribution to total disparity (*t* = 4.84, df = 9, *P* < 1e^−3^; Fig. 4c). Sample size was positively correlated with species richness (*t* = 4.69, df = 9, *P* = 0.001; Fig. S10).

## Discussion

### Floral disparity through time

Early high levels of disparity are generally characterized by high rates of character change during the early phases of a lineage's history, resulting in maximum phenotypic variation followed by developmental canalization (Foote, 1997, 1999; Wagner, 2018; Smith & Donoghue, 2022). As we show here, angiosperm flowers also followed this pattern, reaching their maximal disparity in the Early Cretaceous, meaning that major structural variations (Bauplan, e.g. fusion, number, and arrangement of organs) were explored early in the history of the group. Interestingly, this occurred when angiosperm species richness was still low compared with other land plants (Lupia *et al*., 1999) and the group was still mainly restricted to the paleotropics (Willis & McElwain, 2002). It remains uncertain whether the initial floral morphological diversification was associated with the exploitation of ecological opportunities, for example by the interaction with dispersers and pollinators. Insect pollination, for instance, existed before the origin of angiosperms (Peñalver *et al*., 2015; Asar *et al*., 2022; Peña‐Kairath *et al*., 2023) and is ancestral for the group as a whole (Friis *et al*., 1999; Stephens *et al*., 2023).

Disparity decreased in the Late Cretaceous as the average floral morphology shifted to become more similar to that of extant species (Fig. S11). For example, the higher frequency of undifferentiated free perianths, spiral androecia, basifixed anthers, and valvate anther dehiscence among Early Cretaceous fossils was, from the Late Cretaceous on, replaced by a majority of differentiated and fused perianths, whorled androecia, dorsifixed anthers, and dehiscence by longitudinal slits. A number of these Late Cretaceous flowers also display new traits considered as a step toward a further adaptation to animal pollination, like perianth differentiation, a lower number of floral organs, nectaries, and fusion of carpels (syncarpy; Friis *et al*., 2006). This timing is consistent with the diversification of various pollinating insect clades (Asar *et al*., 2022; Benton *et al*., 2022; Kawahara *et al*., 2023; Peña‐Kairath *et al*., 2023), the emergence (stem age) of most extant angiosperm families (Ramírez‐Barahona *et al*., 2020), and the progressive dominance of angiosperms in terrestrial ecosystems (Lupia *et al*., 1999; Willis & McElwain, 2002; Augusto *et al*., 2014; Condamine *et al*., 2020).

We also observe a decrease in floral disparity following the Cretaceous/Paleogene (K/Pg) extinction event. Although fossil‐based quantitative analyses have suggested that the K/Pg extinction was highly selective to certain clades (e.g. Laurales and Cyclanthaceae; McElwain & Punyasena, 2007) and regions (e.g. tropical South America; Carvalho *et al*., 2021), there is no consensus about the impact of the K/Pg extinction on angiosperms as a whole (Willis & McElwain, 2002; Cascales‐Miñana & Cleal, 2014; Thompson & Ramírez‐Barahona., 2023; Wilf *et al*., 2023), and it remains uncertain whether it had an effect on floral disparity as Paleogene floral fossils are unevenly distributed with regard to phylogeny, geography, and preservation types (Friis *et al*., 2011; Xing *et al*., 2016). In our data, the morphospace area covered by these fossils is restricted, and the area emptied during the Paleogene gets occupied again in the Present, confirming a possible sample bias in the Paleogene record. The Paleogene was characterized by profound global landscape changes with the emergence of dense subtropical forests with closed canopies (Carvalho *et al*., 2021) and the diversification (crown age) of most extant angiosperm families (Ramírez‐Barahona *et al*., 2020). Despite their lower disparity, Paleogene flowers show a higher frequency of bilateral symmetry (zygomorphy) and perianth fusion, which might indicate higher rates of specialization to different functional groups of pollinators (e.g. Stewart *et al*., 2022).

Interestingly, angiosperm pollen disparity also rapidly increased from the Early Cretaceous (Lupia, 1999) but the highest pollen disparity in the Late Cretaceous was followed by a stabilization of the morphospace due to the accumulation of different morphologies. The same pattern was estimated for Asterales pollen (Jardine *et al*., 2022). Conversely, Oyston *et al*. (2016) found that the overall disparity of vegetative and reproductive characters remained constant throughout the angiosperm's history. By contrast, a study on wood traits (Martínez‐Cabrera *et al*., 2017) found low initial disparity followed by an increase from the Late Cretaceous on, concomitant with the increased domination of land ecosystems by angiosperms, most probably due to vegetative key innovations leading to more adaptive growth strategies (Feild *et al*., 2011; Condamine *et al*., 2020). The different evolutionary trends of disparity variation between flowers, pollen, and vegetative traits probably reflect differences in function, selective value, and evolutionary potential of these traits. Knowing more about these different components would allow getting a more comprehensive understanding of the incredible success of angiosperms throughout their evolutionary history.

### Limits in the evolution of floral form

Most extant flowers are restricted to a small area of the morphospace (Fig. S6): 91% of the living species have flowers with a perianth, 85% are bisexual, 83% are whorled, and 72% have a differentiated perianth (Fig. S7). In terms of trait combinations, the most common flower is bisexual, with a differentiated perianth arranged in two whorls (45% of living species in our dataset). These traits characterize *Pentapetalae*, the angiosperm clade containing > 70% of extant species (Magallon *et al*., 1999; Christenhusz & Byng, 2016). For the flower, the higher occupation of some areas of the morphospace can be attributed to the economy of construction and/or to functional advantages (Stebbins, 1951; Endress, 1982). For example, bisexuality (85% of the living species in our dataset) increases pollination probability (function) since pollinators can deposit and take up pollen in a single flower visit; a differentiated perianth (72% of the living species in our dataset) allows sepals to protect the developing flower while petals attract pollinators at anthesis (function); a perianth with two whorls of organs (71% of the living species in our dataset) that generally alternate allows for optimal organization (economy of construction); finally, the presence of a low number of united carpels (55% of the living species in our dataset) requires a smaller amount of wall tissue during growth (economy of construction) and enables more regular pollen tube distribution among carpels (function; Endress, 1982), increasing the probability for fertilization (Armbruster *et al*., 2002). Some trait combinations, like the presence of a corolla with zygomorphy and a small number of stamens (20% of the living species in our dataset), increase speciation rates (O'Meara *et al*., 2016) as they give a selective advantage for pollination (function) by ensuring a more efficient placement of pollen on pollinator's bodies (Walker‐Larsen & Harder, 2000; Sargent, 2004).

The distribution of living species in the floral morphospace and the quantification of disparity follow a phylogenetic pattern and support conclusions from qualitative studies (e.g. Endress & Igersheim, 1997, 1999, 2000; Matthews & Endress, 2002, 2005, 2006; Schönenberger & von Balthazar, 2006; Endress, 2010, 2011). These results are consistent with the re‐analysis of Stebbin's dataset by Chartier *et al*. (2014): we found the highest floral disparity in early‐diverging and species‐poor grades and clades, and the lowest disparity in highly nested and species‐rich clades (Fig. 3).

The ANA grade, magnoliids, and the other eudicots grade display a broad range of floral trait combinations appearing widely spread in the morphospace. In addition, most species with the highest eccentricity belong to these groups (Fig. S11). It is expected for grades to show more variability than clades per definition. Old clades are also known to present more variable floral traits. For instance, floral phyllotaxis is particularly labile in members of the ANA grade and magnoliids (Endress & Doyle, 2009), where whorled and spiral phyllotaxis often coexist at shallow phylogenetic levels, and merism is highly variable within early‐diverging angiosperms and members of the other eudicots grade (Endress, 2011). The most eccentric species often show a marked reduction of organs, such as perianth loss, allowing the arrangement and number of stamens and carpels to become highly labile (e.g. *Eupomatia* and *Galbulimina* in the magnoliids, *Euptelea*, and *Trochodendron* in the other eudicots grade, and *Cyclanthus* in the other monocots grade; Endress, 2011).

Another trend is the stabilization of floral organization within highly nested groups such as malvids, campanulids, and lamiids, which occupy relatively narrow regions in the space and show low levels of disparity coupled with high species richness (Fig. 3). Some floral characters became canalized or fixed within these major clades. For example, floral phyllotaxis is generally whorled and merism became stabilized to pentamery (or tetramery) in Pentapetalae and trimery in commelinids (Endress, 2011). This tendency in angiosperm floral evolution toward fixation of the number and position of organs, and toward repeated evolution of some traits such as zygomorphy (Reyes *et al*., 2016), is often associated with increased synorganization, that is when organs of the same module (e.g. perianth) or different modules (e.g. androecium and gynoecium) become integrated into architecturally and functionally complex structures (Endress, 2002, 2006, 2016). The lower disparity in floral organization found in nested clades does not preclude, and possibly even has enabled variation in other aspects of floral structure, for instance, traits associated with mechanical properties (e.g. size and proportions) or traits directly related to interactions with pollinators (e.g. organ shape, floral color, and rewards). Such floral traits, which have not been considered in this study, tend to be very labile, even among closely related species (Endress, 1994, 2011).

### Conclusion

Our quantification of disparity through time revealed a pattern of highest disparity early during angiosperm floral evolution. Disparity in the Early Cretaceous might have been even higher than our present estimation since numerous plant fossils with reproductive organs possibly belonging to the angiosperms have not been formally described yet (Sauquet & Magallón, 2018). Studies on disparity would greatly benefit from the description of such fossils, even if they cannot be assigned to extant lineages. It seems very surprising that there are hardly any described extinct angiosperm families and orders, which one would expect when considering the long evolutionary history of the group (but see Sun *et al*., 2002; Pessoa *et al*., 2023). Other fossils, such as *Bevhalstia pebja* Hill, 1996 (Friis *et al*., 2011), do not present enough characters to be added to disparity studies. It also has to be mentioned that the fossil record of angiosperms is geographically biased since most of the currently available specimens stem from the mid‐northern paleolatitudes (Friis *et al*., 2011; Xing *et al*., 2016). Studies in extant lineages have shown that floral disparity may vary with latitude and with regional factors (Chartier *et al*., 2021), and it would be interesting to test whether such patterns are also present in the floral fossil record. However, it is currently not possible to address this question due to the paucity of the floral fossil record from the southern hemisphere. Furthermore, our results illustrate that ASRs are, by nature, conservative. First, while the ages of the crown nodes of the evaluated groups date to the early Cretaceous (or earlier; Ramírez‐Barahona *et al*., 2020; Table S1), the corresponding ASRs fall within the small morphospace areas occupied by most extant species included in the dataset (Fig. 2). This is discrepant with the documented positive correlation between node age and disparity (Fig. 4), and hence, with our expectation that oldest nodes would occupy morphospace areas outside those occupied by the majority of extant species. Second, while ASRs may document character state combinations that are not present among the original species sample, they are methodologically precluded from estimating character states that are not present in this sample. Third, our results empirically show that fossils document greater floral disparity than ASRs (Fig. 2). Nonetheless, further ASR‐based analyses would complement our approach as they would likely add crucial qualitative and quantitative information on the evolution of the group, through the exploration of morphological rates of evolution through time and through ASR incorporating fossil data. Finally, the analyses of morphospaces for vegetative and for other reproductive characters (e.g., fruits) would achieve an integrative understanding of angiosperm evolution.

## Competing interests

None declared.

## Author contributions

MC, AML‐M, HS, JS, MvB and SM designed the study. MvB, JS, AML‐M and HS scored and curated the data for fossils. HS provided the scripts for ancestral state reconstructions. MC and AML‐M conducted the analyses. AML‐M and MC wrote the paper. All co‐authors interpreted the results and edited the manuscript.

## Supporting information


**Dataset S1** Script used to generate the distance matrix, the ordination of the space, and calculate *D*.

**Dataset S2** Morphological dataset, information about scored fossils, and about study groups.

**Dataset S3** Proteus extraction used to generate the dataset, containing all references.

**Dataset S4** Ancestral state reconstructions for each character obtained with a maximum likelihood approach.

**Dataset S5** Ancestral state reconstructions for each character obtained with a stochastic mapping approach.

**Fig. S1** Distribution of missing and nonapplicable data for living species.
**Fig. S2** Distribution of missing and nonapplicable data for fossil species.
**Fig. S3** Rarefied floral disparity (*meanD* and *R*) through time.
**Fig. S4** Position of Neogene fossils in the floral morphospace (Fig. 1).
**Fig. S5** Distribution of character states in the morphospace (Fig. 2).
**Fig. S6** Position of eccentric species in the morphospace ordination.
**Fig. S7** Distribution of character states in living Angiosperms.
**Fig. S8** Rarefied floral disparity (*meanD* and *R*) for living angiosperms.
**Fig. S9** Position of additional living angiosperm clades in the floral morphospace (Fig. 3).
**Fig. S10** Correlation between sample size and group sizes.
**Fig. S11** Phylogenetic distribution of species in the four time bins studied and for eccentric species.
**Methods S1** List of nonapplicable and impossible combinations.
**Methods S2** Calculation of the mean character difference.
**Table S1** Divergence time estimates for the 15 ASR.
**Table S2** Disparity (*meanD*) for the study angiosperm group.
Please note: Wiley is not responsible for the content or functionality of any Supporting Information supplied by the authors. Any queries (other than missing material) should be directed to the *New Phytologist* Central Office.

## Data Availability

Data are available in the Supporting Information.
