# Supplementary material for: Angiosperm flowers reached their highest morphological diversity early in their evolutionary history
Source: New Phytol. 2023 Nov 29;241(3):1348–60. doi: 10.1111/nph.19389 (PMC10952840; doi:10.1111/nph.19389)
Supplement: Supplementary file 1 — Dataset S1 Script used to generate the distance matrix, the ordination of the space, and calculate D. [file NPH-241-1348-s003.pdf]

New Phytologist Supporting Information Data S1

- Content
- How to use
- Initialisation
- Preparation of the data
- Computation of the distance matrix
- PCoA
  - Plot
  - Variance explained by the axes
  - Mantel test between the distance matrix and distances displayed in the graph
- nMDS
  - Plot
  - Shepard Plot
- Statistical test (PERMANOVA) for differences among groups

Content

Distance matrix, PCoA, nMDS and PERMANOVA as computed in Lopez-Martinez et al. 2023 New Phytologist.

Article title:

Angiosperm flowers reached their highest morphological diversity early in their evolutionary history

Authors:

Andrea M. López-Martínez, Susana Magallón, Maria von Balthazar, Jürg Schönenberger, Hervé Sauquet, Marion Chartier

Article acceptance date:

20 October 2023

How to use

This file contains a function, `MD()` , written by Sylvain Gerber, and modified by Marion Chartier, as well as examples on how to perform a PCoA, a nMDS, a PERMANOVA and the corresponding posthoc tests, using usual functions from R (including package vegan).
Please be aware that these are our working files, and that they were not debugged to work with all kinds of datasets, nor cleaned. If you have questions please contact Marion Chartier (chartier.marion@gmail.com (mailto:chartier.marion@gmail.com)).

Initialisation

Here we load the file `data` , in which we simulated different kinds of characters, including missing data, columns/rows with only missing data (that should be removed automatically during the analysis) and one column for which all data are coded the same (that should be removed is asked by the user).
!!!!! decimal separator must be `.` !!!!!
In the dataset, categorical characters must be coded with consecutive numbers, starting at 0.

```
rm(list=ls())
citation()
```

```
##
## To cite R in publications use:
##
##   R Core Team (2022). R: A language and environment for statistical
##   computing. R Foundation for Statistical Computing, Vienna, Austria.
##   URL https://www.R-project.org/.
##
## A BibTeX entry for LaTeX users is
##
##   @Manual{,
##     title = {R: A Language and Environment for Statistical Computing},
##     author = {{R Core Team}},
##     organization = {R Foundation for Statistical Computing},
##     address = {Vienna, Austria},
##     year = {2022},
##     url = {https://www.R-project.org/},
##   }
##
## We have invested a lot of time and effort in creating R, please cite it
## when using it for data analysis. See also 'citation("pkgname")' for
## citing R packages.
```

```
tab=read.csv2("data.csv", dec = ".")
tab
```

```
##    species group    colour char1 char2 char3 char4 char5c char6c char7c
## 1      sp1   gr1 #c000c0ff   1.2   0.2    NA     1      0      0      0
## 2      sp2   gr1 #c000c0ff   3.5   2.5    NA     2      0      0      0
## 3      sp3   gr1 #c000c0ff   2.1   1.1    NA     3      0      0      3
## 4      sp4   gr1 #c000c0ff   1.2   0.2    NA     4      0      0      0
## 5      sp5   gr1 #c000c0ff   2.0   1.0    NA     1      5      0      3
## 6      sp6   gr2 #00ae00ff   5.8    NA    NA     2      2      0      1
## 7      sp7   gr2 #00ae00ff   6.7   5.7    NA     3      2      0      1
## 8      sp8   gr2 #00ae00ff   5.6   4.6    NA     4      2      0     NA
## 9      sp9   gr2 #00ae00ff   4.0   3.0    NA     1      2      0      1
## 10    sp10   gr2 #00ae00ff   7.0   6.0    NA     2      3      0      2
## 11    sp11   gr2 #00ae00ff   4.5   3.5    NA     3      4      0      1
## 12    sp12   gr2 #00ae00ff   5.6   4.6    NA     4      2      0      3
## 13    sp13   gr2 #00ae00ff   6.0   5.0    NA     1      1      0      1
## 14    sp14   gr3 #ffcc00ff   3.0   2.0    NA     2      1      0      1
## 15    sp15   gr3 #ffcc00ff   3.1   2.1    NA     2      1      0      2
## 16    sp16   gr3 #ffcc00ff    NA    NA    NA    NA     NA     NA     NA
## 17    sp17   gr3 #ffcc00ff   3.3   2.3    NA     2      0      0      2
## 18    sp18   gr3 #ffcc00ff   3.1   2.1    NA     1      1      0      2
## 19    sp19   gr3 #ffcc00ff   4.0   3.0    NA     1      0      0      1
```

```
source("F_DISPARITY_functions vs5.R")
MD
```

```
## function (X, ochar, equal.w = TRUE, removinvar = FALSE)
## {
##     irr <- c()
##     for (i in 1:nrow(X)) irr <- c(irr, length(which(is.na(X[i,
##         ]) == F)))
##     remtax <- length(which(irr == 0))
##     REMTAX = which(irr == 0)
##     nX = nrow(X)
##     xname = c(1:nX)
##     xname = cbind(xname, xname, xname)
##     colnames(xname) = c("xname", "Dname", "realkill")
##     xname = data.frame(xname)
##     lremtax = length(REMTAX)
##     xname$Dname[REMTAX] = 9999
##     xname = xname[order(xname$Dname), ]
##     xname$Dname = c(c(1:(nX - remtax)), rep(9999, remtax))
##     if (remtax != 0) {
##         print("Uninformative taxa removed:", quote = FALSE)
##         print(as.numeric(which(irr == 0)))
##         X <- X[-c(which(irr == 0)), ]
##     }
##     w <- apply(X, 2, max, na.rm = T, show.error.messages = FALSE) -
##         apply(X, 2, min, na.rm = T, show.error.messages = FALSE)
##     bad <- which(w == 0)
##     nabad = integer(0)
##     for (i in 1:ncol(X)) {
##         if (length(unique(X[, i])) == 1 & all(is.na(unique(X[,
##             i])) == TRUE)) {
##             nabad = c(nabad, i)
##         }
##     }
##     bad <- sort(unique(bad))
##     nabad = sort(unique(nabad))
##     if (length(nabad != 0)) {
##         print("Columns containing NAs only and thus not included:",
##             quote = FALSE)
##         print(as.numeric(nabad))
##     }
##     if (removinvar == T) {
##         if (length(bad != 0)) {
##             print("Unvarying characters not included:", quote = FALSE)
##             print(as.numeric(bad))
##         }
##     }
##     else {
##         if (length(bad != 0)) {
##             print("Unvarying characters still included:", quote = FALSE)
##             print(as.numeric(bad))
##         }
##     }
##     w[bad] = 1
##     preochar <- rep(0, ncol(X))
##     preochar[ochar] <- 1
##     if (length(nabad) != 0)
```

```
##         X <- X[, -c(nabad)]
##     if (removinvar == T) {
##         if (length(bad) != 0) {
##             X = X[, -c(bad)]
##         }
##     }
##     D <- diag(0, nrow(X))
##     Q = D
##     FOOTE = D
##     if (length(nabad) != 0)
##         preochar <- preochar[-c(nabad)]
##     ochar <- which(preochar == 1)
##     if (length(nabad) != 0)
##         w <- w[-c(nabad)]
##     no <- c(1:dim(X)[2])[-c(ochar)]
##     w[no] <- 1
##     pw <- combn(nrow(X), 2)
##     kill <- c()
##     for (i in 1:ncol(pw)) {
##         d <- (abs(X[pw[1, i], ] - X[pw[2, i], ]))
##         d[no][which(d[no] > 1)] <- 1
##         d <- d * (1/w)
##         d2 <- sum(na.omit(d))/(length(na.omit(d)))
##         Q[pw[1, i], pw[2, i]] <- length(na.omit(d))/length(d)
##         d <- c(na.omit(d), rep(d2, length(d) - length(na.omit(d))))
##         d <- sqrt(sum(unlist(d)^2))
##         if (is.nan(d) == TRUE)
##             kill <- c(kill, c(pw[, i]))
##         D[pw[1, i], pw[2, i]] <- d
##         FOOTE[pw[1, i], pw[2, i]] <- d2
##     }
##     DD <- D + t(D)
##     FOOTE2 <- FOOTE + t(FOOTE)
##     if (length(kill) != 0) {
##         killmat <- cbind(unique(kill), rep(0, length(unique(kill))))
##         for (i in 1:dim(killmat)[1]) killmat[i, 2] <- length(which(kill ==
##             killmat[i, 1]))
##         order <- rank(killmat[, 2], ties.method = "f")
##         killmat[order, 2] <- killmat[, 2]
##         killmat[order, 1] <- killmat[, 1]
##         i <- dim(killmat)[1]
##         while (length(which(is.nan(D) == T)) != 0) {
##             D[c(killmat[i, 1]), ] <- 999
##             D[, c(killmat[i, 1])] <- 999
##             FOOTE[c(killmat[i, 1]), ] <- 999
##             FOOTE[, c(killmat[i, 1])] <- 999
##             i <- i - 1
##         }
##         DD <- D + t(D)
##         FOOTE2 <- FOOTE + t(FOOTE)
##         kill = killmat[(i + 1):nrow(killmat), 1]
##         if (length(kill) != 0) {
##             D <- D[-c(kill), -c(kill)]
##             Q <- Q[-c(kill), -c(kill)]
##             FOOTE <- FOOTE[-c(kill), -c(kill)]
```

```
##         }
##     }
##     D <- D + t(D)
##     Q <- Q + t(Q)
##     diag(Q) = 1
##     FOOTE <- FOOTE + t(FOOTE)
##     lkill = length(kill)
##     xname$realkill = xname$Dname
##     for (i in 1:length(kill)) {
##         xname$realkill[which(xname$Dname == kill[i])] = 99999
##     }
##     xname = xname[order(xname$realkill), ]
##     REALKILL = c()
##     if ((lkill + lremtax) > 0) {
##         REALKILL = xname$xname[(nX - lkill - lremtax + 1):(nX)]
##         REALKILL = REALKILL[order(REALKILL)]
##         nb = c()
##         for (i in (1:length(REMTAX))) {
##             nb = c(nb, which(REALKILL == REMTAX[i]))
##         }
##         if (lkill > 0) {
##             if (length(nb) > 0) {
##                 print("Taxa removed to produce distance matrix:",
##                   quote = FALSE)
##                 print(REALKILL[-nb])
##             }
##             else {
##                 print("Taxa removed to produce distance matrix:",
##                   quote = FALSE)
##                 print(REALKILL)
##             }
##         }
##     }
##     remtax = which(irr == 0)
##     remtax = unique(c(which(irr == 0), REALKILL))
##     list(DD = DD, D = D, Q = Q, FOOTE = FOOTE, kill = REALKILL,
##         FOOTE2 = FOOTE2, remtax = remtax, remchar = nabad)
## }
```

```
library(vegan)
```

```
## Loading required package: permute
```

```
## Loading required package: lattice
```

```
## This is vegan 2.5-7
```

Preparation of the data

Here, we introduce `ochar`, a vector containing the list of columns of the data matrix `CHAR` that are NOT categorical (ie that are numerical or categorical ordered). For these characters, the distance will be

calculated as the difference between two species divided by the range of the character in the whole matrix. For all other columns, the distance will be 0 if the two species are different, 1 if they are coded the same.

!!!!! VS5 OF THE FUNCTION DOES NOT WORK IF REMOVINVAR=T _check before that you have no invariable column (eg a column with only `1`)!!!!!

```
tab
```

```
##    species group    colour char1 char2 char3 char4 char5c char6c char7c
## 1      sp1   gr1 #c000c0ff   1.2   0.2    NA     1      0      0      0
## 2      sp2   gr1 #c000c0ff   3.5   2.5    NA     2      0      0      0
## 3      sp3   gr1 #c000c0ff   2.1   1.1    NA     3      0      0      3
## 4      sp4   gr1 #c000c0ff   1.2   0.2    NA     4      0      0      0
## 5      sp5   gr1 #c000c0ff   2.0   1.0    NA     1      5      0      3
## 6      sp6   gr2 #00ae00ff   5.8    NA    NA     2      2      0      1
## 7      sp7   gr2 #00ae00ff   6.7   5.7    NA     3      2      0      1
## 8      sp8   gr2 #00ae00ff   5.6   4.6    NA     4      2      0     NA
## 9      sp9   gr2 #00ae00ff   4.0   3.0    NA     1      2      0      1
## 10    sp10   gr2 #00ae00ff   7.0   6.0    NA     2      3      0      2
## 11    sp11   gr2 #00ae00ff   4.5   3.5    NA     3      4      0      1
## 12    sp12   gr2 #00ae00ff   5.6   4.6    NA     4      2      0      3
## 13    sp13   gr2 #00ae00ff   6.0   5.0    NA     1      1      0      1
## 14    sp14   gr3 #ffcc00ff   3.0   2.0    NA     2      1      0      1
## 15    sp15   gr3 #ffcc00ff   3.1   2.1    NA     2      1      0      2
## 16    sp16   gr3 #ffcc00ff    NA    NA    NA    NA     NA     NA     NA
## 17    sp17   gr3 #ffcc00ff   3.3   2.3    NA     2      0      0      2
## 18    sp18   gr3 #ffcc00ff   3.1   2.1    NA     1      1      0      2
## 19    sp19   gr3 #ffcc00ff   4.0   3.0    NA     1      0      0      1
```

```
CHAR=tab[,4:10]
CHAR
```

```
##    char1 char2 char3 char4 char5c char6c char7c
## 1    1.2   0.2    NA     1      0      0      0
## 2    3.5   2.5    NA     2      0      0      0
## 3    2.1   1.1    NA     3      0      0      3
## 4    1.2   0.2    NA     4      0      0      0
## 5    2.0   1.0    NA     1      5      0      3
## 6    5.8    NA    NA     2      2      0      1
## 7    6.7   5.7    NA     3      2      0      1
## 8    5.6   4.6    NA     4      2      0     NA
## 9    4.0   3.0    NA     1      2      0      1
## 10   7.0   6.0    NA     2      3      0      2
## 11   4.5   3.5    NA     3      4      0      1
## 12   5.6   4.6    NA     4      2      0      3
## 13   6.0   5.0    NA     1      1      0      1
## 14   3.0   2.0    NA     2      1      0      1
## 15   3.1   2.1    NA     2      1      0      2
## 16    NA    NA    NA    NA     NA     NA     NA
## 17   3.3   2.3    NA     2      0      0      2
## 18   3.1   2.1    NA     1      1      0      2
## 19   4.0   3.0    NA     1      0      0      1
```

```
ochar=c(1:4)
```

Computation of the distance matrix

Note that it is important the CHAR is a MATRIX, if not the function doesn't work.

```
dis=MD(as.matrix(CHAR),ochar,removinvar=F)
```

```
## [1] Uninformative taxa removed:
## [1] 16
## [1] Columns containing NAs only and thus not included:
## [1] 3
## [1] Unvarying characters still included:
## [1] 3 6
```

In vs5 of the function, missing data are counted as unvarying characters if the columns only contain missing data.

```
names(dis)
```

```
## [1] "DD"      "D"       "Q"       "FOOTE"   "kill"    "FOOTE2"  "remtax"
## [8] "remchar"
```

The objects you are interested in are:

`dis\$remtax` is the list of taxa (rows) that have been removed during the calculation,
`dis\$remchar` is the list of characters (columns) that have been removed from `CHAR` during the calculation,
`dis\$FOOTE` is the distance matrix.

PCoA

Plot

```
mapco=PCO(dis$FOOTE, correction=" ")
```

```
## Warning in sqrt(S$values): NaNs produced
```

```
plot(mapco$pco, col=as.character(tab$colour[-dis$remtax]), pch=16, cex=1.3)
```

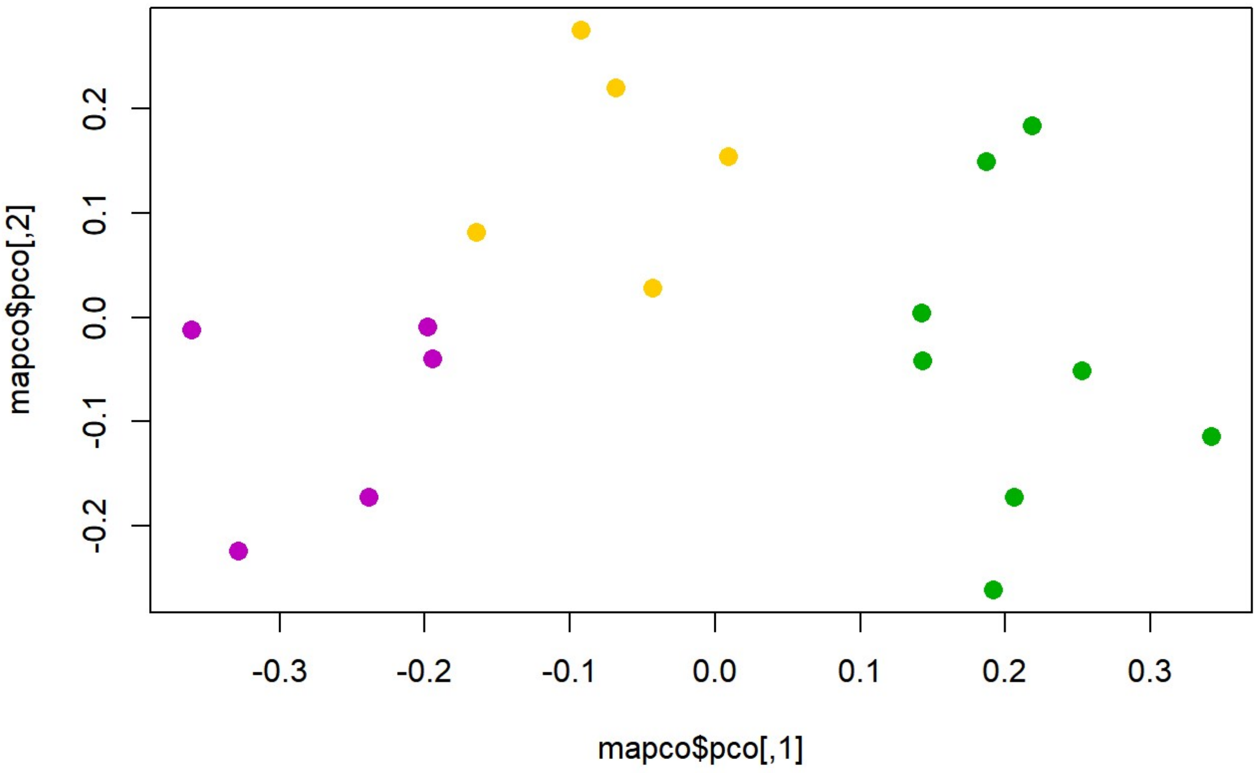

Variance explained by the axes

```
Eig=mapco$PCAEval[mapco$PCAEval>0]
var1=Eig[1]*100/sum(Eig)
var2=Eig[2]*100/sum(Eig)
var3=Eig[3]*100/sum(Eig)

c(var1, var2)
```

```
## [1] 40.84933 20.96984
```

var1 to 3 give the percentage of variance explained by the axes.

Mantel test between the distance matrix and distances displayed in the graph

```
Dismatrix=dis$FOOTE
Disgraph=dist(mapco$pco[,1:2])
mantel(Disgraph,Dismatrix) #renvoit un coefficient de Pearson.
```

```
##
## Mantel statistic based on Pearson's product-moment correlation
##
## Call:
## mantel(xdis = Disgraph, ydis = Dismatrix)
##
## Mantel statistic r: 0.8146
##       Significance: 0.001
##
## Upper quantiles of permutations (null model):
##   90%   95% 97.5%   99%
## 0.120 0.167 0.201 0.244
## Permutation: free
## Number of permutations: 999
```

```
par(mar=rep(4,4))
plot(as.dist(Disgraph),as.dist(Dismatrix), xlim=c(0,0.6), ylim=c(0,0.6),  pch=16, cex=0.5)
abline(0,1, col="red")
```

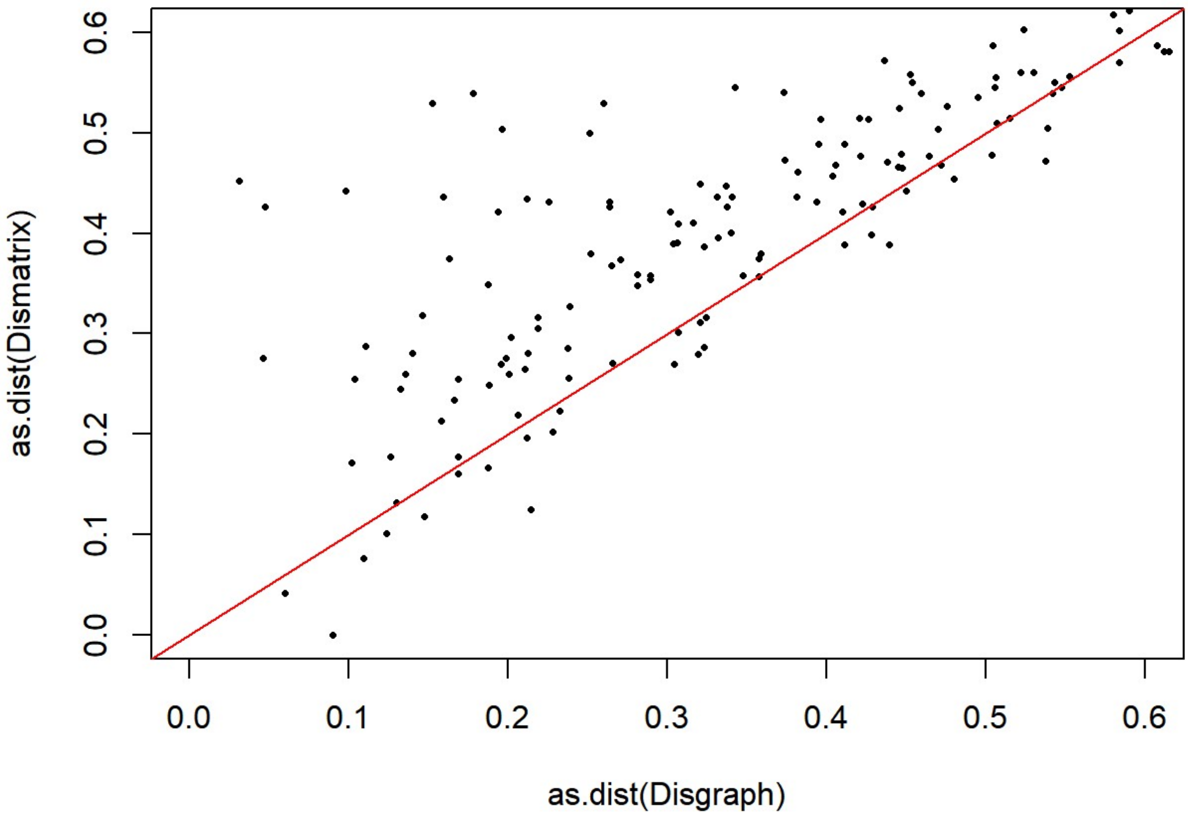

nMDS

Plot

```
myNMDS=metaMDS(dis$FOOTE, k=2, trymax=20, wascores=F, autotransform=F, noshare=F)
```

```
## Run 0 stress 0.1540275
## Run 1 stress 0.156512
## Run 2 stress 0.1833413
## Run 3 stress 0.156512
## Run 4 stress 0.1540275
## ... Procrustes: rmse 4.757451e-06  max resid 1.190263e-05
## ... Similar to previous best
## Run 5 stress 0.1817349
## Run 6 stress 0.1540275
## ... Procrustes: rmse 1.240188e-05  max resid 2.94862e-05
## ... Similar to previous best
## Run 7 stress 0.1540275
## ... New best solution
## ... Procrustes: rmse 4.69491e-06  max resid 1.228552e-05
## ... Similar to previous best
## Run 8 stress 0.1540275
## ... New best solution
## ... Procrustes: rmse 1.977078e-06  max resid 5.421487e-06
## ... Similar to previous best
## Run 9 stress 0.1540275
## ... Procrustes: rmse 2.307173e-05  max resid 6.625908e-05
## ... Similar to previous best
## Run 10 stress 0.1832671
## Run 11 stress 0.1802844
## Run 12 stress 0.1833413
## Run 13 stress 0.1573323
## Run 14 stress 0.1540275
## ... Procrustes: rmse 5.668454e-06  max resid 1.745679e-05
## ... Similar to previous best
## Run 15 stress 0.1540275
## ... Procrustes: rmse 5.617631e-06  max resid 1.348082e-05
## ... Similar to previous best
## Run 16 stress 0.1540275
## ... Procrustes: rmse 9.225e-06  max resid 1.854986e-05
## ... Similar to previous best
## Run 17 stress 0.1540275
## ... Procrustes: rmse 1.327287e-06  max resid 2.657885e-06
## ... Similar to previous best
## Run 18 stress 0.1540275
## ... Procrustes: rmse 2.632417e-06  max resid 8.851007e-06
## ... Similar to previous best
## Run 19 stress 0.1833413
## Run 20 stress 0.1540275
## ... Procrustes: rmse 9.021782e-06  max resid 3.060712e-05
## ... Similar to previous best
## *** Solution reached
```

```
x=myNMDS$points[,1]
y=myNMDS$points[,2]

plot(x, y, col=as.character(tab$colour[-dis$remtax]), pch=16, cex=1.3)
```

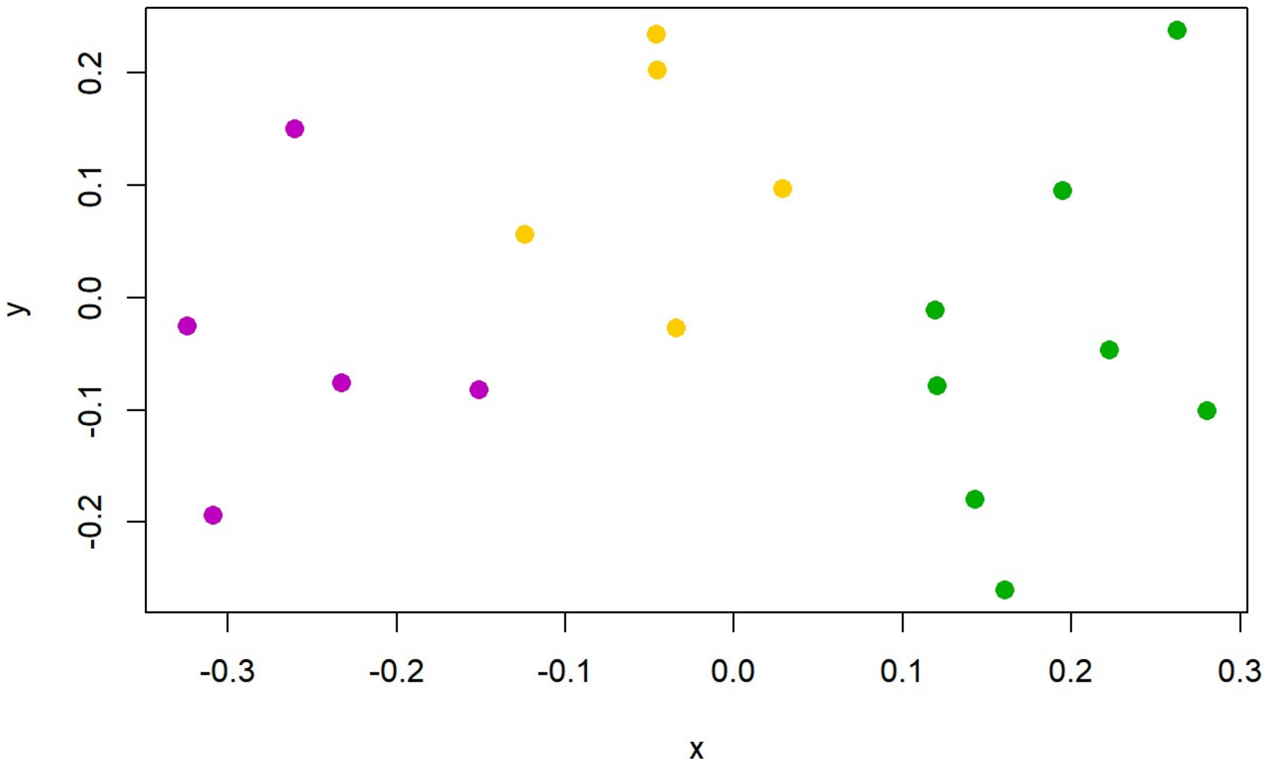

Shepard Plot

This plot gives an indiaction about the quality of the ordination. It is good to report at least the stress value and the result of the correlation between the ordination distances and the observed dissimilarities (distance matrix) in the results.

```
stressplot(myNMDS,dis$FOOTE, main="Shepard plot", ylim=c(0,1), xlim=c(0,1), pch=20, p.col
="#0000FF80")
lines(c(0,1),c(0,1), col="#ff7f00", lwd=1.5)
```

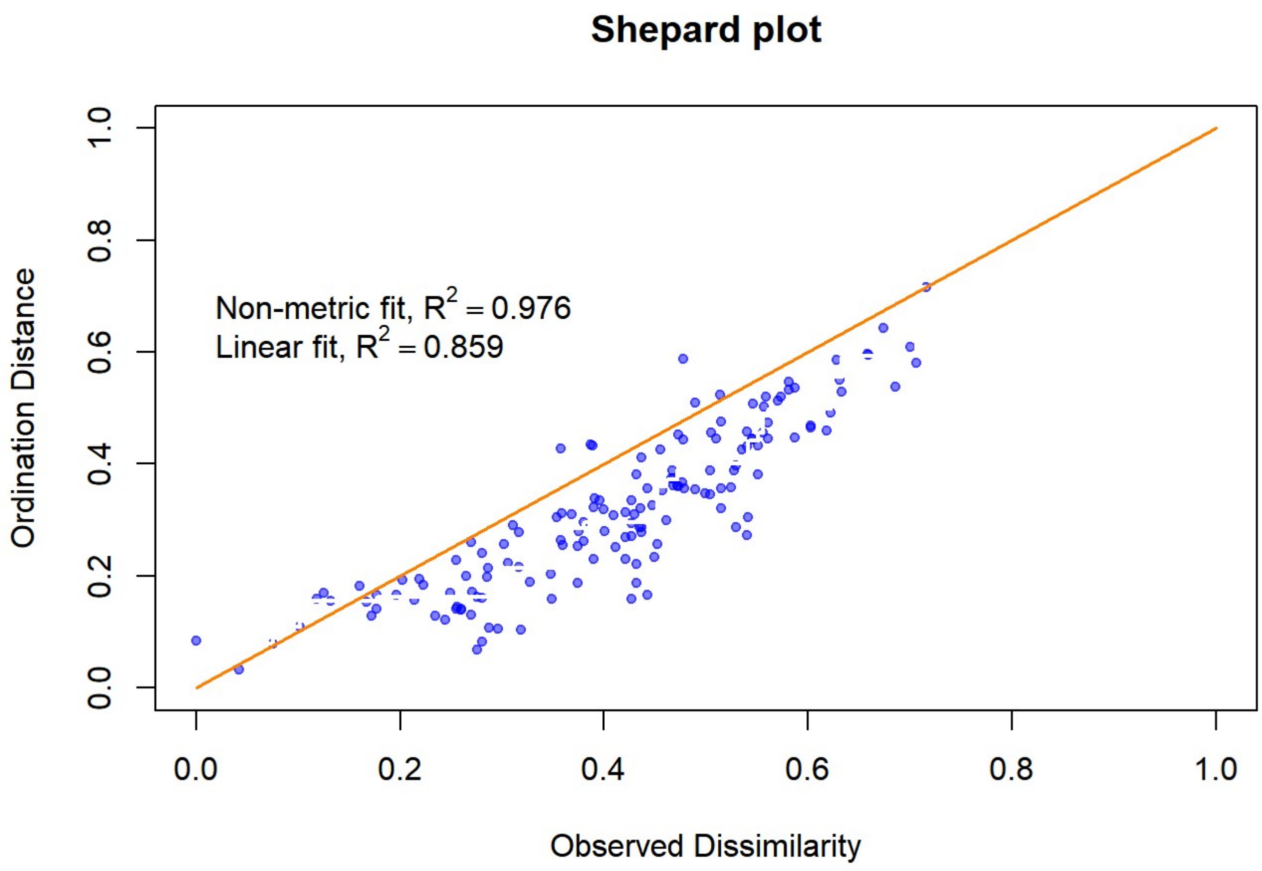


```
paste("Stress value: ",round(myNMDS$stress, digit=2), sep="")
```

```
## [1] "Stress value: 0.15"
```

```
paste("No species: ", nrow(tab), sep="")
```

```
## [1] "No species: 19"
```

Statistical test (PERMANOVA) for differences among groups

Using functions from package vegan.

```
source("F_adonisph.r")
adonisph
```

```
## function (DATA, GROUP, METH = "bray", removezero = F, permutations = 9999)
## {
##     posthoc = NULL
##     posthoc2 = NULL
##     a = levels(as.factor(GROUP))
##     posthoc = matrix(NA, nrow = length(a), ncol = length(a))
##     colnames(posthoc) = a
##     rownames(posthoc) = a
##     posthoc = as.data.frame(posthoc)
##     posthoc2 = posthoc
##     c = ncol(posthoc)
##     n = (c * c - c)/2
##     Bonf = 0.05/n
##     for (i in 1:(length(a) - 1)) {
##         for (j in (i + 1):length(a)) {
##             tab2 = DATA[GROUP == a[i] | GROUP == a[j], ]
##             group2 = GROUP[GROUP == a[i] | GROUP == a[j]]
##             if (removezero == T) {
##                 k = 1
##                 while (k <= ncol(tab2)) {
##                   if (all(tab2[, k] == 0)) {
##                     tab2 = tab2[, -k]
##                   }
##                   else k = k + 1
##                 }
##             }
##             ado = adonis(tab2 ~ as.factor(group2), method = as.character(METH),
##                 permutations = permutations)
##             p = ado$aov.tab$Pr[1]
##             r = ado$aov.tab$R2[1]
##             f = ado$aov.tab$F.Model[1]
##             posthoc[i, j] <- round(r, digit = 3)
##             posthoc[j, i] <- round(p, digit = 4)
##             if (posthoc[j, i] > Bonf) {
##                 posthoc2[j, i] = "ns"
##             }
##             else {
##                 posthoc2[j, i] = "*"
##             }
##             posthoc2[i, j] <- round(f, digit = 3)
##             rm(ado, p, r, f)
##         }
##     }
##     res <- list(posthoc = posthoc, posthoc2 = posthoc2, pvalue = paste("Corrected p-val
ue: ",
##         round(Bonf, digit = 6)))
##     return(res)
## }
```

```
library(vegan)
citation("vegan")
```

```
##
## To cite package 'vegan' in publications use:
##
##   Jari Oksanen, F. Guillaume Blanchet, Michael Friendly, Roeland Kindt,
##   Pierre Legendre, Dan McGlinn, Peter R. Minchin, R. B. O'Hara, Gavin
##   L. Simpson, Peter Solymos, M. Henry H. Stevens, Eduard Szoecs and
##   Helene Wagner (2020). vegan: Community Ecology Package. R package
##   version 2.5-7. https://CRAN.R-project.org/package=vegan
##
## A BibTeX entry for LaTeX users is
##
##   @Manual{,
##     title = {vegan: Community Ecology Package},
##     author = {Jari Oksanen and F. Guillaume Blanchet and Michael Friendly and Roeland K
indt and Pierre Legendre and Dan McGlinn and Peter R. Minchin and R. B. O'Hara and Gavin
L. Simpson and Peter Solymos and M. Henry H. Stevens and Eduard Szoecs and Helene Wagner},
##     year = {2020},
##     note = {R package version 2.5-7},
##     url = {https://CRAN.R-project.org/package=vegan},
##   }
##
## ATTENTION: This citation information has been auto-generated from the
## package DESCRIPTION file and may need manual editing, see
## 'help("citation")'.
```

```
if(length(dis$kill)==0){
    per=adonis2(dis$FOOTE~tab$group, method="bray", permutation=9999)
}else{ per=adonis2(dis$FOOTE~tab$group[-dis$remtax], method="bray", permutation=9999) }

per
```

```
## Permutation test for adonis under reduced model
## Terms added sequentially (first to last)
## Permutation: free
## Number of permutations: 9999
##
## adonis2(formula = dis$FOOTE ~ tab$group[-dis$remtax], permutations = 9999, method = "br
ay")
##                          Df SumOfSqs      R2      F Pr(>F)
## tab$group[-dis$remtax]  2  0.90243 0.55236 9.2545  1e-04 ***
## Residual                15  0.73135 0.44764
## Total                   17  1.63378 1.00000
## ---
## Signif. codes:  0 '***' 0.001 '**' 0.01 '*' 0.05 '.' 0.1 ' ' 1
```

And finally the posthoc test (pairwise PERMANOVAS with a Bonferroni correction):

```
if(length(dis$remtax)==0){
    adph=adonisph(dis$FOOTE, tab$group, permutation=999)
    }else{
    adph=adonisph(dis$FOOTE, tab$group[-dis$remtax], permutation=999)
    }

adph$posthoc
```

```
##       gr1   gr2   gr3
## gr1    NA 0.603 0.462
## gr2 0.002    NA 0.477
## gr3 0.011 0.001    NA
```

```
adph$posthoc2
```

```
##      gr1    gr2    gr3
## gr1 <NA> 16.738  6.875
## gr2    *   <NA> 10.025
## gr3    *      *     NA
```

```
adph$pvalue
```

```
## [1] "Corrected p-value:  0.016667"
```

In `posthoc` , r2 is given in the upper part of the table, p (non corrected) in the lower part.
In `posthoc2` , F is given in the upper part of the table, and `*` or `ns` in the lower part of the table, showing which comparisons are significantly different after the Bonferroni correction.
Note that the function “adonisph” has the option of removing columns containing “0” only, that can be activated by the option `removezero=T` . This is to be used in case of comparisons made on scent data, for ex.

```
## [1] "Marion CHARTIER | University of Vienna | 20231026."
## [1] "Please cite our article and the authors of the used R packages."
```
