# Supplementary material for: Angiosperm flowers reached their highest morphological diversity early in their evolutionary history
Source: New Phytol. 2023 Nov 29;241(3):1348–60. doi: 10.1111/nph.19389 (PMC10952840; doi:10.1111/nph.19389)

New Phytologist **Supporting Information Data S4**

Article title: Angiosperm flowers reached their highest morphological diversity early in their evolutionary history

Authors: Andrea M. López-Martínez, Maria von Balthazar, Jürg Schönenberger, Susana Magallón, Hervé Sauquet, Marion Chartier

Article acceptance date: 20 October 2023

Content: Ancestral state reconstructions for each character obtained with a maximum likelihood approach.

ML ancestral state reconstruction using rayDISC (R:corHMM)
100_B. Structural sex of flowers (D2d), ARD model

- bisexual
- unisexual

Node	ML state	Prob
Angiospermae	bisexual	0.9423
Mesangiospermae	bisexual	0.9997
Magnoliidae	bisexual	1
Monocotyledoneae	bisexual	1
Eudicotyledoneae	bisexual	1
Commelinidae	bisexual	1
Pentapetalae	bisexual	1
Superasteridae	bisexual	1
Asteridae	bisexual	1
Lamiidae	bisexual	0.9999
Campanulidae	bisexual	1
Superrosidae	bisexual	1
Rosidae	bisexual	1
Malvidae	bisexual	1
Fabidae	bisexual	1

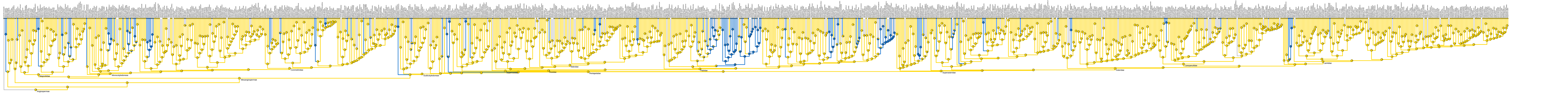

ML ancestral state reconstruction using rayDISC (R:corHMM)
100_B. Structural sex of flowers (D2d), ER model

bisexual
unisexual

Node	ML state	Prob
Angiospermae	bisexual	0.987
Mesangiospermae	bisexual	1
Magnoliidae	bisexual	1
Monocotyledoneae	bisexual	1
Eudicotyledoneae	bisexual	1
Commelinidae	bisexual	1
Pentapetalae	bisexual	1
Superasteridae	bisexual	1
Asteridae	bisexual	1
Lamiidae	bisexual	1
Campanulidae	bisexual	1
Superrosidae	bisexual	1
Rosidae	bisexual	1
Malvidae	bisexual	1
Fabidae	bisexual	1

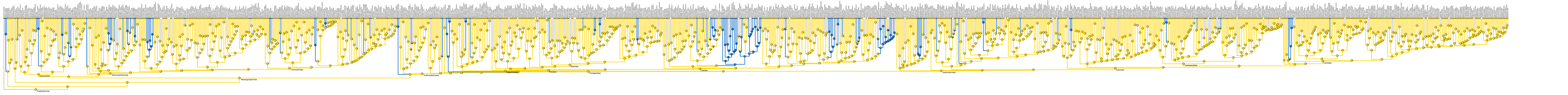

ML ancestral state reconstruction using rayDISC (R:corHMM)
102_B. Ovary position (binary) (D2d), ARD model

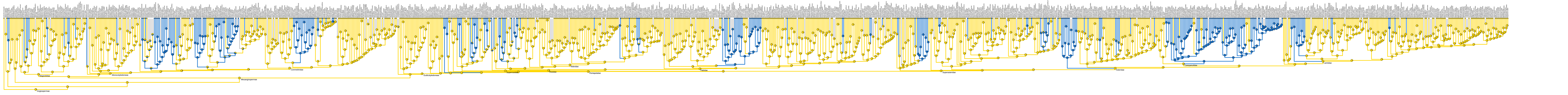

Node	ML state	Prob
Angiospermae	superior	0.9959
Mesangiospermae	superior	1
Magnoliidae	superior	1
Monocotyledoneae	superior	0.9998
Eudicotyledoneae	superior	1
Commelinidae	superior	0.9985
Pentapetalae	superior	1
Superasteridae	superior	1
Asteridae	superior	0.9993
Lamiidae	superior	0.9997
Campanulidae	superior	0.9063
Superrosidae	superior	1
Rosidae	superior	1
Malvidae	superior	1
Fabidae	superior	1

ML ancestral state reconstruction using rayDISC (R:corHMM)
102_B. Ovary position (binary) (D2d), ER model

- superior
- inferior

Node	ML state	Prob
Angiospermae	superior	0.9953
Mesangiospermae	superior	1
Magnoliidae	superior	1
Monocotyledoneae	superior	0.9997
Eudicotyledoneae	superior	1
Commelinidae	superior	0.9975
Pentapetalae	superior	1
Superasteridae	superior	1
Asteridae	superior	0.9992
Lamiidae	superior	0.9997
Campanulidae	superior	0.8963
Superrosidae	superior	1
Rosidae	superior	1
Malvidae	superior	1
Fabidae	superior	1

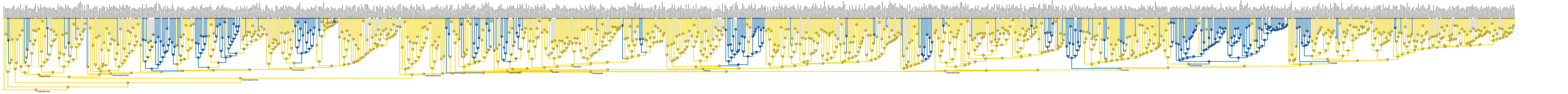

ML ancestral state reconstruction using rayDISC (R:corHMM)
201_A. Perianth presence (D2c), ARD model

Node	ML state	Prob
Angiospermae	absent	0.996
Mesangiospermae	absent	0.9999
Magnoliidae	absent	0.9989
Monocotyledoneae	absent	0.9983
Eudicotyledoneae	absent	0.9381
Commelinidae	present	0.9194
Pentapetalae	present	0.6551
Superasteridae	present	0.975
Asteridae	present	0.9983
Lamiidae	present	0.9999
Campanulidae	present	0.9999
Superrosidae	present	0.8857
Rosidae	present	0.9391
Malvidae	present	1
Fabidae	present	0.9978

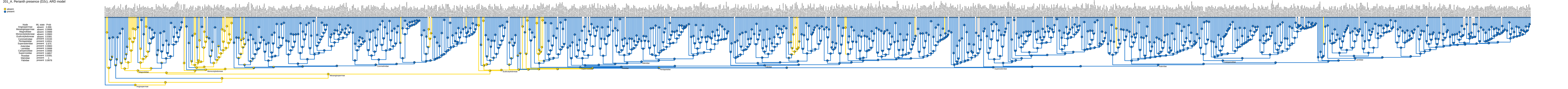

ML ancestral state reconstruction using rayDISC (R:corHMM)
201_A. Perianth presence (D2c), ER model

- absent
- present

Node	ML state	Prob
Angiospermae	present	0.9997
Mesangiospermae	present	1
Magnoliidae	present	1
Monocotyledoneae	present	1
Eudicotyledoneae	present	1
Commelinidae	present	1
Pentapetalae	present	1
Superasteridae	present	1
Asteridae	present	1
Lamiidae	present	1
Campanulidae	present	1
Superrosidae	present	1
Rosidae	present	1
Malvidae	present	1
Fabidae	present	1

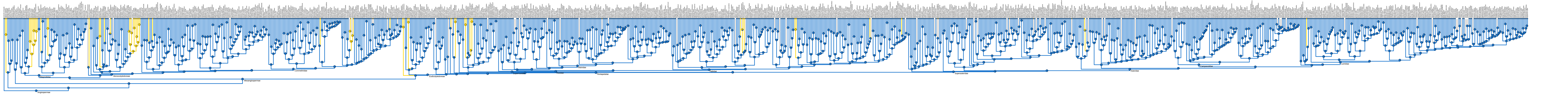

ML ancestral state reconstruction using rayDISC (R:corHMM)
201_B. Number of perianth parts (3−state) (D2c), ARD model

- one to five (1−5)
- six to ten (6−10)
- more than ten (>10)

Node	ML state	Prob
Angiospermae	more than ten (>10)	0.9929
Mesangiospermae	more than ten (>10)	0.9999
Magnoliidae	more than ten (>10)	0.9998
Monocotyledoneae	six to ten (6−10)	0.8164
Eudicotyledoneae	more than ten (>10)	0.9972
Commelinidae	six to ten (6−10)	1
Pentapetalae	six to ten (6−10)	0.8827
Superasteridae	six to ten (6−10)	0.9993
Asteridae	six to ten (6−10)	1
Lamiidae	six to ten (6−10)	1
Campanulidae	six to ten (6−10)	1
Superrosidae	six to ten (6−10)	0.9994
Rosidae	six to ten (6−10)	0.9996
Malvidae	six to ten (6−10)	1
Fabidae	six to ten (6−10)	1

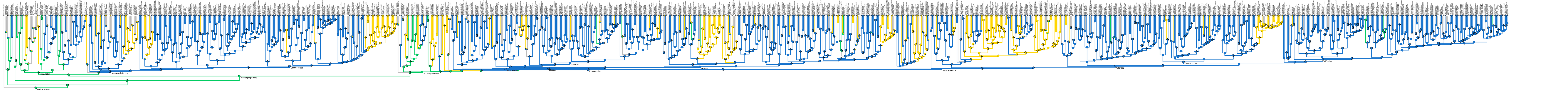

ML ancestral state reconstruction using rayDISC (R:corHMM)
201_B. Number of perianth parts (3−state) (D2c), ER model

- one to five (1−5)
- six to ten (6−10)
- more than ten (>10)

Node	ML state	Prob
Angiospermae	six to ten (6−10)	0.9315
Mesangiospermae	six to ten (6−10)	0.9993
Magnoliidae	six to ten (6−10)	0.9989
Monocotyledoneae	six to ten (6−10)	1
Eudicotyledoneae	six to ten (6−10)	0.9935
Commelinidae	six to ten (6−10)	1
Pentapetalae	six to ten (6−10)	0.9996
Superasteridae	six to ten (6−10)	1
Asteridae	six to ten (6−10)	1
Lamiidae	six to ten (6−10)	1
Campanulidae	six to ten (6−10)	1
Superrosidae	six to ten (6−10)	1
Rosidae	six to ten (6−10)	1
Malvidae	six to ten (6−10)	1
Fabidae	six to ten (6−10)	1

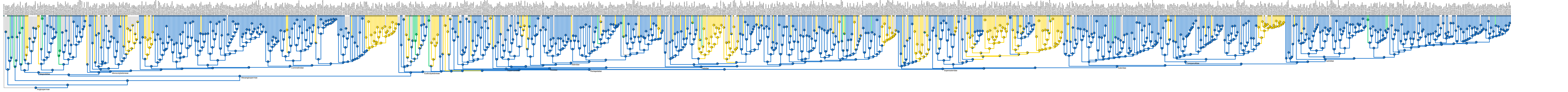

ML ancestral state reconstruction using rayDISC (R:corHMM)
204_A. Fusion of perianth (D2c), ARD model

free (<5%)
fused (>5%)

Node	ML state	Prob
Angiospermae	free (<5%)	0.9937
Mesangiospermae	free (<5%)	1
Magnoliidae	free (<5%)	1
Monocotyledoneae	free (<5%)	1
Eudicotyledoneae	free (<5%)	1
Commelinidae	free (<5%)	0.9996
Pentapetalae	free (<5%)	1
Superasteridae	free (<5%)	0.9952
Asteridae	free (<5%)	0.9237
Lamiidae	fused (>5%)	0.9693
Campanulidae	fused (>5%)	0.9431
Superrosidae	free (<5%)	1
Rosidae	free (<5%)	0.9999
Malvidae	free (<5%)	1
Fabidae	free (<5%)	1

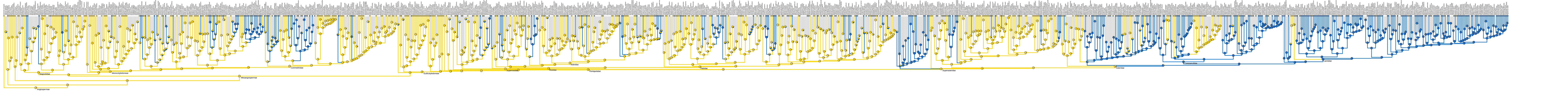

ML ancestral state reconstruction using rayDISC (R:corHMM)
204_A. Fusion of perianth (D2c), ER model

- free (<5%)
- fused (>5%)

Node	ML state	Prob
Angiospermae	free (<5%)	0.9878
Mesangiospermae	free (<5%)	1
Magnoliidae	free (<5%)	0.9999
Monocotyledoneae	free (<5%)	1
Eudicotyledoneae	free (<5%)	1
Commelinidae	free (<5%)	0.9981
Pentapetalae	free (<5%)	0.9999
Superasteridae	free (<5%)	0.9744
Asteridae	free (<5%)	0.852
Lamiidae	fused (>5%)	0.9951
Campanulidae	fused (>5%)	0.9912
Superrosidae	free (<5%)	0.9998
Rosidae	free (<5%)	0.9998
Malvidae	free (<5%)	0.9999
Fabidae	free (<5%)	1

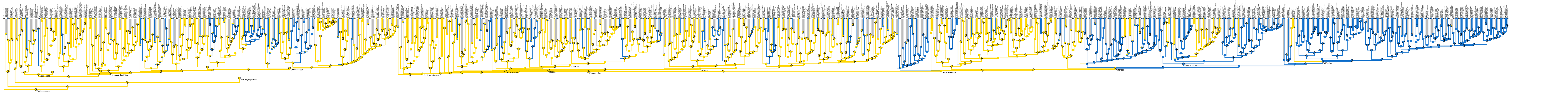

ML ancestral state reconstruction using rayDISC (R:corHMM)
207_A. Symmetry of perianth (binary) (D2d), ARD model

- actinomorphic
- zygomorphic

Node	ML state	Prob
Angiospermae	actinomorphic	0.9842
Mesangiospermae	actinomorphic	1
Magnoliidae	actinomorphic	1
Monocotyledoneae	actinomorphic	0.9997
Eudicotyledoneae	actinomorphic	1
Commelinidae	actinomorphic	0.9997
Pentapetalae	actinomorphic	1
Superasteridae	actinomorphic	1
Asteridae	actinomorphic	1
Lamiidae	actinomorphic	0.9998
Campanulidae	actinomorphic	1
Superrosidae	actinomorphic	1
Rosidae	actinomorphic	1
Malvidae	actinomorphic	1
Fabidae	actinomorphic	1

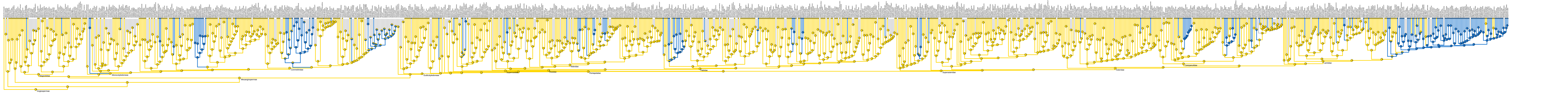

ML ancestral state reconstruction using rayDISC (R:corHMM)
207_A. Symmetry of perianth (binary) (D2d), ER model

- actinomorphic
- zygomorphic

Node	ML state	Prob
Angiospermae	actinomorphic	0.9965
Mesangiospermae	actinomorphic	1
Magnoliidae	actinomorphic	1
Monocotyledoneae	actinomorphic	0.9999
Eudicotyledoneae	actinomorphic	1
Commelinidae	actinomorphic	1
Pentapetalae	actinomorphic	1
Superasteridae	actinomorphic	1
Asteridae	actinomorphic	1
Lamiidae	actinomorphic	1
Campanulidae	actinomorphic	1
Superrosidae	actinomorphic	1
Rosidae	actinomorphic	1
Malvidae	actinomorphic	1
Fabidae	actinomorphic	1

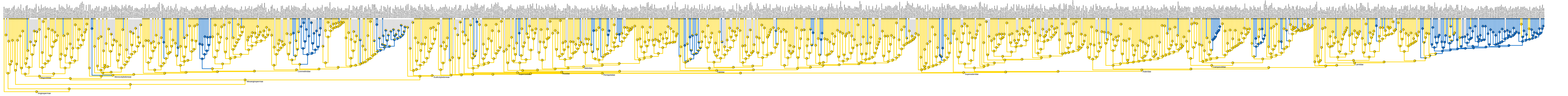

ML ancestral state reconstruction using rayDISC (R:corHMM)
230_A. Perianth phyllotaxy (binary) (D2d), ARD model

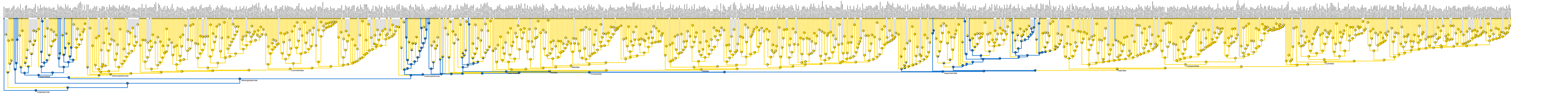

Node	ML state	Prob
Angiospermae	spiral	1
Mesangiospermae	spiral	1
Magnoliidae	spiral	0.9997
Monocotyledoneae	whorled	0.797
Eudicotyledoneae	spiral	1
Commelinidae	whorled	0.9998
Pentapetalae	spiral	0.9899
Superasteridae	spiral	0.9884
Asteridae	whorled	0.7375
Lamiidae	whorled	0.9987
Campanulidae	whorled	0.9947
Superrosidae	spiral	0.9252
Rosidae	spiral	0.566
Malvidae	whorled	0.9986
Fabidae	whorled	0.9814

ML ancestral state reconstruction using rayDISC (R:corHMM)
230_A. Perianth phyllotaxy (binary) (D2d), ER model

- whorled
- spiral

Node	ML state	Prob
Angiospermae	whorled	0.8709
Mesangiospermae	whorled	0.9999
Magnoliidae	whorled	1
Monocotyledoneae	whorled	1
Eudicotyledoneae	whorled	1
Commelinidae	whorled	1
Pentapetalae	whorled	1
Superasteridae	whorled	1
Asteridae	whorled	1
Lamiidae	whorled	1
Campanulidae	whorled	1
Superrosidae	whorled	1
Rosidae	whorled	1
Malvidae	whorled	1
Fabidae	whorled	1

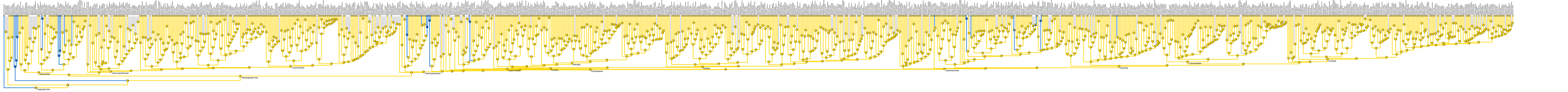

ML ancestral state reconstruction using rayDISC (R:corHMM)
231_A. Number of perianth whorls (D2c), ARD model

- one (1)
- two (2)
- more than two (>2)

Node	ML state	Prob
Angiospermae	more than two (>2)	0.9986
Mesangiospermae	more than two (>2)	0.9998
Magnoliidae	more than two (>2)	1
Monocotyledoneae	two (2)	0.8761
Eudicotyledoneae	more than two (>2)	0.9949
Commelinidae	two (2)	1
Pentapetalae	two (2)	0.9824
Superasteridae	two (2)	1
Asteridae	two (2)	1
Lamiidae	two (2)	1
Campanulidae	two (2)	1
Superrosidae	two (2)	1
Rosidae	two (2)	1
Malvidae	two (2)	1
Fabidae	two (2)	1

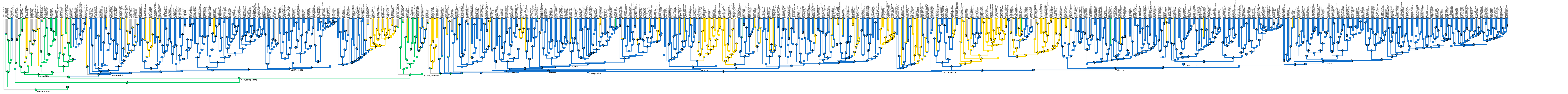

ML ancestral state reconstruction using rayDISC (R:corHMM)
231_A. Number of perianth whorls (D2c), ER model

- one (1)
- two (2)
- more than two (>2)

Node	ML state	Prob
Angiospermae	two (2)	0.8534
Mesangiospermae	two (2)	0.937
Magnoliidae	two (2)	0.8258
Monocotyledoneae	two (2)	0.9982
Eudicotyledoneae	two (2)	0.9414
Commelinidae	two (2)	1
Pentapetalae	two (2)	1
Superasteridae	two (2)	1
Asteridae	two (2)	1
Lamiidae	two (2)	1
Campanulidae	two (2)	1
Superrosidae	two (2)	1
Rosidae	two (2)	1
Malvidae	two (2)	1
Fabidae	two (2)	1

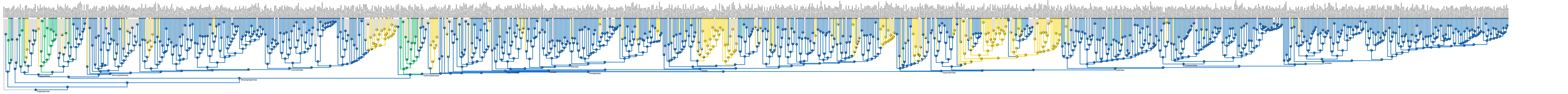

ML ancestral state reconstruction using rayDISC (R:corHMM)
232_A. Perianth merism (4−state) (D2c), ARD model

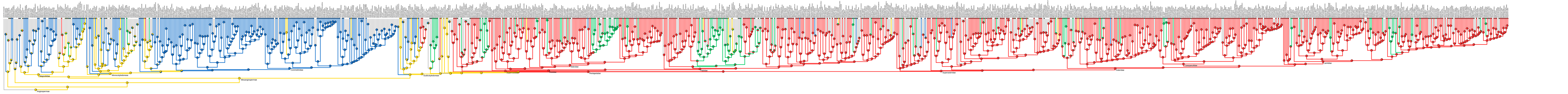

Node	ML state	Prob
Angiospermae	dimerous	0.9654
Mesangiospermae	dimerous	0.9997
Magnoliidae	dimerous	0.9979
Monocotyledoneae	dimerous	0.9864
Eudicotyledoneae	dimerous	0.9979
Commelinidae	trimerous	0.9957
Pentapetalae	pentamerous	0.9116
Superasteridae	pentamerous	0.9999
Asteridae	pentamerous	0.9998
Lamiidae	pentamerous	0.9999
Campanulidae	pentamerous	0.9998
Superrosidae	pentamerous	0.9997
Rosidae	pentamerous	0.9999
Malvidae	pentamerous	1
Fabidae	pentamerous	0.9998

ML ancestral state reconstruction using rayDISC (R:corHMM)
232_A. Perianth merism (4−state) (D2c), ER model

- dimerous
- trimerous
- tetramerous
- pentamerous

Node	ML state	Prob
Angiospermae	trimerous	0.9618
Mesangiospermae	trimerous	0.987
Magnoliidae	trimerous	1
Monocotyledoneae	trimerous	1
Eudicotyledoneae	trimerous	0.8384
Commelinidae	trimerous	1
Pentapetalae	pentamerous	0.9912
Superasteridae	pentamerous	1
Asteridae	pentamerous	1
Lamiidae	pentamerous	1
Campanulidae	pentamerous	1
Superrosidae	pentamerous	1
Rosidae	pentamerous	1
Malvidae	pentamerous	1
Fabidae	pentamerous	1

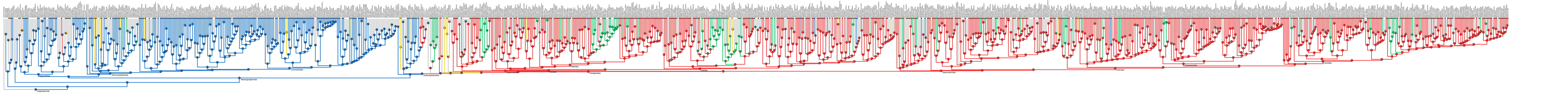

ML ancestral state reconstruction using rayDISC (R:corHMM)
234_B. Perianth differentiation (binary) (D2d), ARD model

- undifferentiated
- differentiated

Node	ML state	Prob
Angiospermae	undifferentiated	0.9853
Mesangiospermae	undifferentiated	0.9969
Magnoliidae	undifferentiated	0.9979
Monocotyledoneae	undifferentiated	0.9999
Eudicotyledoneae	undifferentiated	0.9269
Commelinidae	undifferentiated	0.9999
Pentapetalae	undifferentiated	0.5467
Superasteridae	differentiated	0.5039
Asteridae	differentiated	0.9274
Lamiidae	differentiated	0.9999
Campanulidae	differentiated	0.9999
Superrosidae	differentiated	0.7821
Rosidae	differentiated	0.8547
Malvidae	differentiated	0.998
Fabidae	differentiated	0.9304

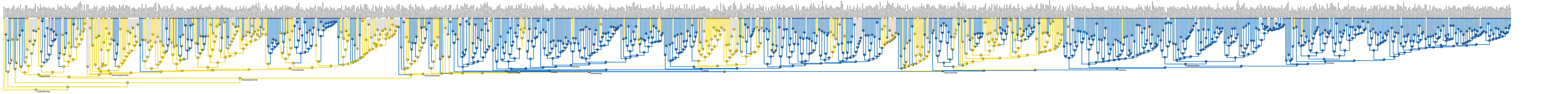

ML ancestral state reconstruction using rayDISC (R:corHMM)
234_B. Perianth differentiation (binary) (D2d), ER model

undifferentiated
differentiated

Node	ML state	Prob
Angiospermae	differentiated	0.9311
Mesangiospermae	differentiated	0.9985
Magnoliidae	differentiated	0.9974
Monocotyledoneae	differentiated	0.8343
Eudicotyledoneae	differentiated	1
Commelinidae	differentiated	0.9536
Pentapetalae	differentiated	1
Superasteridae	differentiated	1
Asteridae	differentiated	1
Lamiidae	differentiated	1
Campanulidae	differentiated	1
Superrosidae	differentiated	1
Rosidae	differentiated	1
Malvidae	differentiated	1
Fabidae	differentiated	1

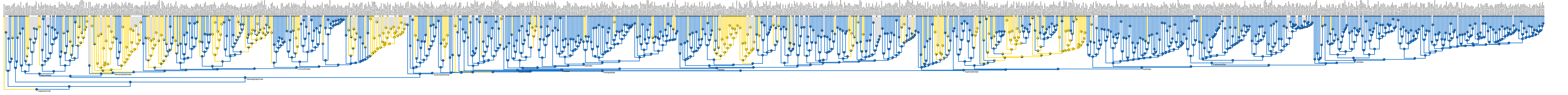

ML ancestral state reconstruction using rayDISC (R:corHMM)
301_B. Number of fertile stamens (3−state) (D2c), ARD model

- one to five (1−5)
- six to ten (6−10)
- more than ten (>10)

Node	ML state	Prob
Angiospermae	more than ten (>10)	0.9981
Mesangiospermae	more than ten (>10)	0.9999
Magnoliidae	more than ten (>10)	0.9995
Monocotyledoneae	six to ten (6−10)	0.8627
Eudicotyledoneae	more than ten (>10)	1
Commelinidae	six to ten (6−10)	1
Pentapetalae	more than ten (>10)	1
Superasteridae	more than ten (>10)	0.9999
Asteridae	more than ten (>10)	0.9982
Lamiidae	one to five (1−5)	0.9999
Campanulidae	one to five (1−5)	0.9976
Superrosidae	more than ten (>10)	1
Rosidae	more than ten (>10)	1
Malvidae	more than ten (>10)	0.9602
Fabidae	more than ten (>10)	0.9999

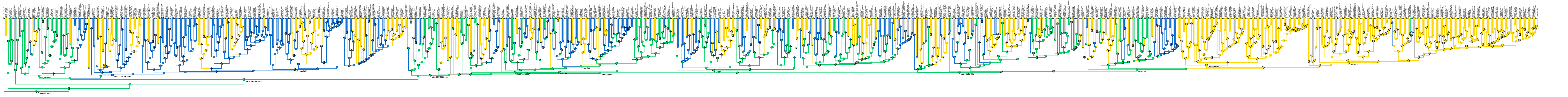

ML ancestral state reconstruction using rayDISC (R:corHMM)
301_B. Number of fertile stamens (3−state) (D2c), ER model

- one to five (1−5)
- six to ten (6−10)
- more than ten (>10)

Node	ML state	Prob
Angiospermae	more than ten (>10)	0.7003
Mesangiospermae	more than ten (>10)	0.505
Magnoliidae	more than ten (>10)	0.6221
Monocotyledoneae	six to ten (6−10)	0.9349
Eudicotyledoneae	one to five (1−5)	0.8217
Commelinidae	six to ten (6−10)	0.9925
Pentapetalae	one to five (1−5)	0.9984
Superasteridae	one to five (1−5)	0.9985
Asteridae	one to five (1−5)	0.9997
Lamiidae	one to five (1−5)	1
Campanulidae	one to five (1−5)	1
Superrosidae	one to five (1−5)	0.9906
Rosidae	one to five (1−5)	0.9448
Malvidae	six to ten (6−10)	0.9891
Fabidae	six to ten (6−10)	0.6234

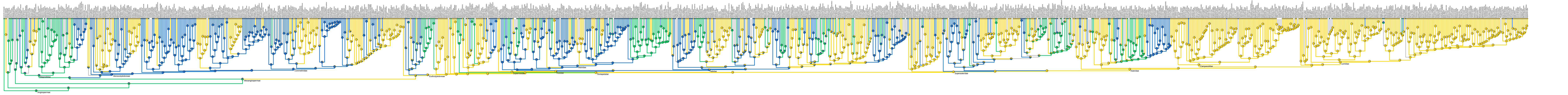

ML ancestral state reconstruction using rayDISC (R:corHMM)
305_A. Filament (binary) (D2d), ARD model

- laminar (wide)
- typical (narrow)

Node	ML state	Prob
Angiospermae	laminar (wide)	0.9775
Mesangiospermae	laminar (wide)	0.9887
Magnoliidae	laminar (wide)	0.9995
Monocotyledoneae	laminar (wide)	0.9812
Eudicotyledoneae	typical (narrow)	0.5408
Commelinidae	laminar (wide)	0.7714
Pentapetalae	typical (narrow)	0.9906
Superasteridae	typical (narrow)	0.9995
Asteridae	typical (narrow)	0.9996
Lamiidae	typical (narrow)	0.9999
Campanulidae	typical (narrow)	0.9972
Superrosidae	typical (narrow)	0.9996
Rosidae	typical (narrow)	0.9998
Malvidae	typical (narrow)	0.9999
Fabidae	typical (narrow)	0.9999

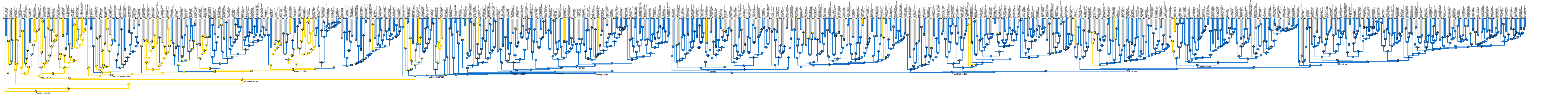

ML ancestral state reconstruction using rayDISC (R:corHMM)
305_A. Filament (binary) (D2d), ER model

- laminar (wide)
- typical (narrow)

Node	ML state	Prob
Angiospermae	typical (narrow)	0.9281
Mesangiospermae	typical (narrow)	0.9914
Magnoliidae	typical (narrow)	0.9253
Monocotyledoneae	typical (narrow)	0.9983
Eudicotyledoneae	typical (narrow)	1
Commelinidae	typical (narrow)	1
Pentapetalae	typical (narrow)	1
Superasteridae	typical (narrow)	1
Asteridae	typical (narrow)	1
Lamiidae	typical (narrow)	1
Campanulidae	typical (narrow)	0.9999
Superrosidae	typical (narrow)	1
Rosidae	typical (narrow)	1
Malvidae	typical (narrow)	1
Fabidae	typical (narrow)	1

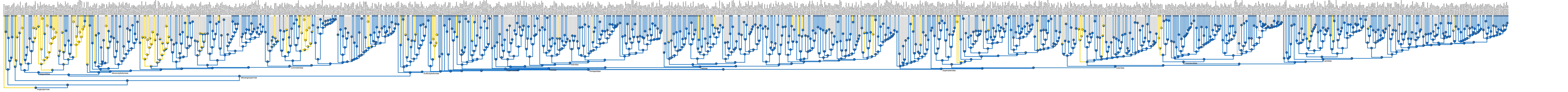

ML ancestral state reconstruction using rayDISC (R:corHMM)
306_A. Fusion of filaments (binary) (D2c), ARD model

free (<5%)
fused (>5%)

Node	ML state	Prob
Angiospermae	fused (>5%)	0.9455
Mesangiospermae	fused (>5%)	0.9799
Magnoliidae	fused (>5%)	0.8314
Monocotyledoneae	fused (>5%)	0.9223
Eudicotyledoneae	fused (>5%)	0.9911
Commelinidae	fused (>5%)	0.9296
Pentapetalae	fused (>5%)	0.9994
Superasteridae	fused (>5%)	1
Asteridae	fused (>5%)	0.998
Lamiidae	free (<5%)	0.8014
Campanulidae	fused (>5%)	0.7792
Superrosidae	fused (>5%)	0.9999
Rosidae	fused (>5%)	0.9999
Malvidae	fused (>5%)	0.9973
Fabidae	fused (>5%)	0.9999

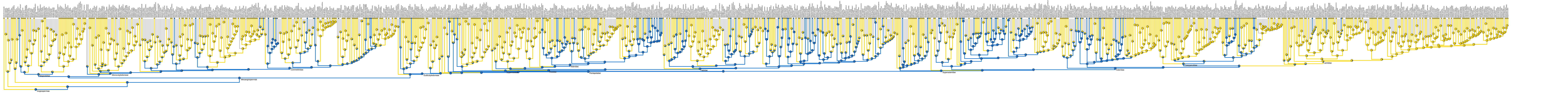

ML ancestral state reconstruction using rayDISC (R:corHMM)
306_A. Fusion of filaments (binary) (D2c), ER model

- free (<5%)
- fused (>5%)

Node	ML state	Prob
Angiospermae	free (<5%)	0.9949
Mesangiospermae	free (<5%)	1
Magnoliidae	free (<5%)	1
Monocotyledoneae	free (<5%)	1
Eudicotyledoneae	free (<5%)	1
Commelinidae	free (<5%)	0.9999
Pentapetalae	free (<5%)	1
Superasteridae	free (<5%)	1
Asteridae	free (<5%)	1
Lamiidae	free (<5%)	1
Campanulidae	free (<5%)	1
Superrosidae	free (<5%)	1
Rosidae	free (<5%)	1
Malvidae	free (<5%)	1
Fabidae	free (<5%)	1

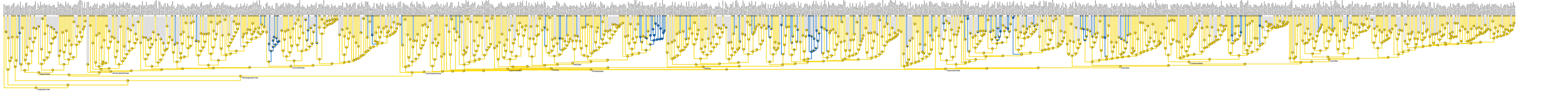

ML ancestral state reconstruction using rayDISC (R:corHMM)
308_A. Fusion of filaments to inner perianth series (binary) (D2c), ARD model

- free (<5%)
- fused (>5%)

Node	ML state	Prob
Angiospermae	free (<5%)	0.9463
Mesangiospermae	free (<5%)	0.9982
Magnoliidae	free (<5%)	0.9993
Monocotyledoneae	free (<5%)	0.9853
Eudicotyledoneae	free (<5%)	0.9961
Commelinidae	fused (>5%)	0.9919
Pentapetalae	free (<5%)	0.9938
Superasteridae	free (<5%)	0.7761
Asteridae	fused (>5%)	0.5391
Lamiidae	fused (>5%)	0.9982
Campanulidae	fused (>5%)	0.9755
Superrosidae	free (<5%)	0.9989
Rosidae	free (<5%)	0.9993
Malvidae	free (<5%)	1
Fabidae	free (<5%)	0.9999

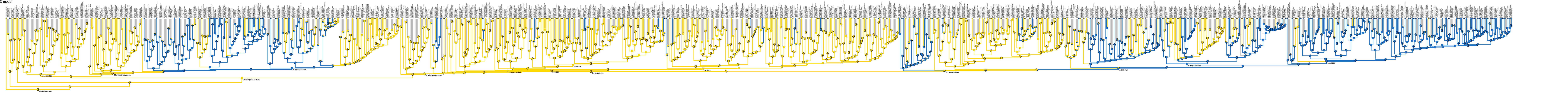

ML ancestral state reconstruction using rayDISC (R:corHMM)
308_A. Fusion of filaments to inner perianth series (binary) (D2c), ER model

free (<5%)
fused (>5%)

Node	ML state	Prob
Angiospermae	free (<5%)	0.9894
Mesangiospermae	free (<5%)	1
Magnoliidae	free (<5%)	1
Monocotyledoneae	free (<5%)	0.9996
Eudicotyledoneae	free (<5%)	1
Commelinidae	free (<5%)	0.8493
Pentapetalae	free (<5%)	1
Superasteridae	free (<5%)	0.9999
Asteridae	free (<5%)	0.9948
Lamiidae	fused (>5%)	0.7084
Campanulidae	free (<5%)	0.9927
Superrosidae	free (<5%)	1
Rosidae	free (<5%)	1
Malvidae	free (<5%)	1
Fabidae	free (<5%)	0.9999

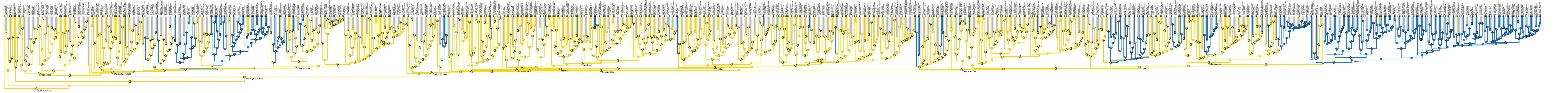

ML ancestral state reconstruction using rayDISC (R:corHMM)
311_A. Anther orientation (D2d), ARD model

- introrse
- latrorse
- extrorse

Node	ML state	Prob
Angiospermae	introrse	0.9568
Mesangiospermae	introrse	0.9713
Magnoliidae	extrorse	0.6554
Monocotyledoneae	introrse	0.9802
Eudicotyledoneae	introrse	0.984
Commelinidae	introrse	0.9999
Pentapetalae	introrse	1
Superasteridae	introrse	1
Asteridae	introrse	0.9996
Lamiidae	introrse	1
Campanulidae	introrse	1
Superrosidae	introrse	1
Rosidae	introrse	1
Malvidae	introrse	1
Fabidae	introrse	1

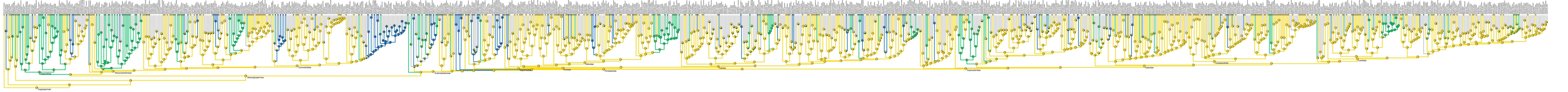

ML ancestral state reconstruction using rayDISC (R:corHMM)
311_A. Anther orientation (D2d), ER model

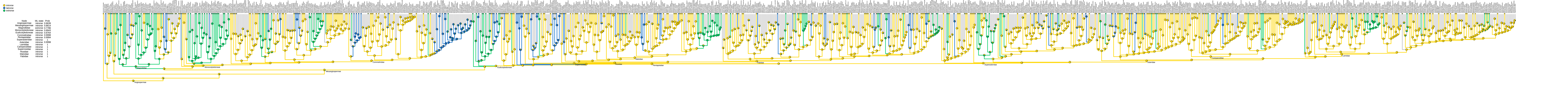

Node	ML state	Prob
Angiospermae	introrse	0.8639
Mesangiospermae	introrse	0.8614
Magnoliidae	extrorse	0.7786
Monocotyledoneae	introrse	0.8943
Eudicotyledoneae	introrse	0.8764
Commelinidae	introrse	0.9998
Pentapetalae	introrse	0.9994
Superasteridae	introrse	1
Asteridae	introrse	0.9998
Lamiidae	introrse	1
Campanulidae	introrse	1
Superrosidae	introrse	1
Rosidae	introrse	1
Malvidae	introrse	1
Fabidae	introrse	1

ML ancestral state reconstruction using rayDISC (R:corHMM)
312_A. Anther attachment (binary) (D2d), ARD model

- basifixed
- dorsifixed

Node	ML state	Prob
Angiospermae	basifixed	0.5
Mesangiospermae	basifixed	0.5466
Magnoliidae	basifixed	0.5466
Monocotyledoneae	basifixed	0.5466
Eudicotyledoneae	basifixed	0.5466
Commelinidae	basifixed	0.5466
Pentapetalae	basifixed	0.5466
Superasteridae	basifixed	0.5466
Asteridae	basifixed	0.5466
Lamiidae	basifixed	0.5466
Campanulidae	basifixed	0.5466
Superrosidae	basifixed	0.5466
Rosidae	basifixed	0.5466
Malvidae	basifixed	0.5466
Fabidae	basifixed	0.5466

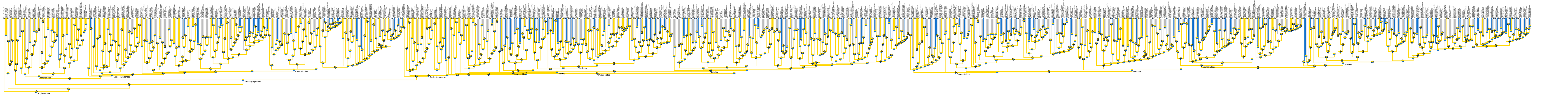

ML ancestral state reconstruction using rayDISC (R:corHMM)
312_A. Anther attachment (binary) (D2d), ER model

- basifixed
- dorsifixed

Node	ML state	Prob
Angiospermae	basifixed	0.5
Mesangiospermae	basifixed	0.5
Magnoliidae	basifixed	0.5
Monocotyledoneae	basifixed	0.5
Eudicotyledoneae	basifixed	0.5
Commelinidae	basifixed	0.5
Pentapetalae	basifixed	0.5
Superasteridae	basifixed	0.5
Asteridae	basifixed	0.5
Lamiidae	basifixed	0.5
Campanulidae	basifixed	0.5
Superrosidae	basifixed	0.5
Rosidae	basifixed	0.5
Malvidae	basifixed	0.5
Fabidae	basifixed	0.5

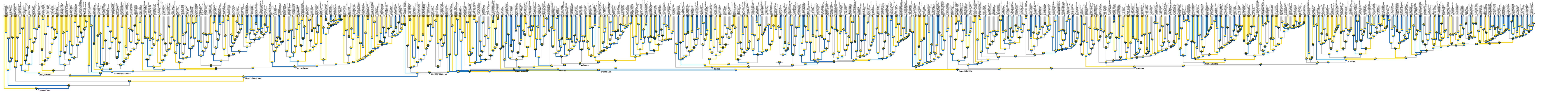

ML ancestral state reconstruction using rayDISC (R:corHMM)
313_A. Anther dehiscence (3−state) (D2d), ARD model

- longitudinal slit
- H−valvate
- flap−valvate

Node	ML state	Prob
Angiospermae	H−valvate	0.9977
Mesangiospermae	H−valvate	1
Magnoliidae	H−valvate	1
Monocotyledoneae	longitudinal slit	0.5548
Eudicotyledoneae	H−valvate	1
Commelinidae	longitudinal slit	0.9998
Pentapetalae	longitudinal slit	0.8014
Superasteridae	longitudinal slit	0.969
Asteridae	longitudinal slit	0.9991
Lamiidae	longitudinal slit	1
Campanulidae	longitudinal slit	1
Superrosidae	longitudinal slit	0.8929
Rosidae	longitudinal slit	0.9379
Malvidae	longitudinal slit	1
Fabidae	longitudinal slit	0.9984

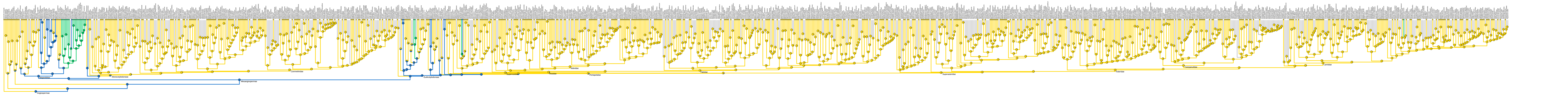

ML ancestral state reconstruction using rayDISC (R:corHMM)
313_A. Anther dehiscence (3−state) (D2d), ER model

- longitudinal slit
- H−valvate
- flap−valvate

Node	ML state	Prob
Angiospermae	longitudinal slit	1
Mesangiospermae	longitudinal slit	1
Magnoliidae	longitudinal slit	1
Monocotyledoneae	longitudinal slit	1
Eudicotyledoneae	longitudinal slit	1
Commelinidae	longitudinal slit	1
Pentapetalae	longitudinal slit	1
Superasteridae	longitudinal slit	1
Asteridae	longitudinal slit	1
Lamiidae	longitudinal slit	1
Campanulidae	longitudinal slit	1
Superrosidae	longitudinal slit	1
Rosidae	longitudinal slit	1
Malvidae	longitudinal slit	1
Fabidae	longitudinal slit	1

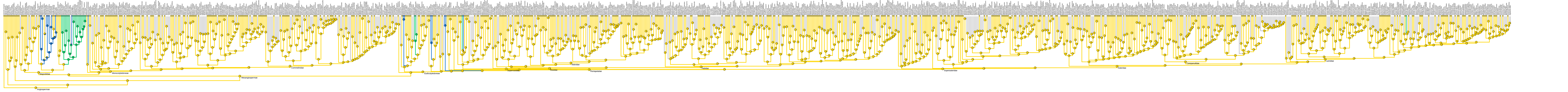

ML ancestral state reconstruction using rayDISC (R:corHMM)
314_A. Connective extension (apical) (D2d), ARD model

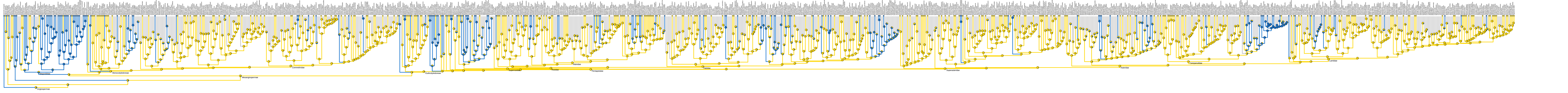

Node	ML state	Prob
Angiospermae	absent	0.7648
Mesangiospermae	absent	0.8673
Magnoliidae	present	0.5222
Monocotyledoneae	absent	0.9511
Eudicotyledoneae	absent	0.9339
Commelinidae	absent	1
Pentapetalae	absent	0.9925
Superasteridae	absent	1
Asteridae	absent	0.9999
Lamiidae	absent	0.9999
Campanulidae	absent	1
Superrosidae	absent	0.9994
Rosidae	absent	0.9996
Malvidae	absent	0.9999
Fabidae	absent	0.9999

ML ancestral state reconstruction using rayDISC (R:corHMM)
314_A. Connective extension (apical) (D2d), ER model

- absent
- present

Node	ML state	Prob
Angiospermae	absent	0.592
Mesangiospermae	absent	0.6958
Magnoliidae	present	0.6676
Monocotyledoneae	absent	0.8838
Eudicotyledoneae	absent	0.8109
Commelinidae	absent	0.9999
Pentapetalae	absent	0.9721
Superasteridae	absent	0.9998
Asteridae	absent	0.9998
Lamiidae	absent	0.9998
Campanulidae	absent	0.9999
Superrosidae	absent	0.9971
Rosidae	absent	0.9981
Malvidae	absent	0.9999
Fabidae	absent	0.9997

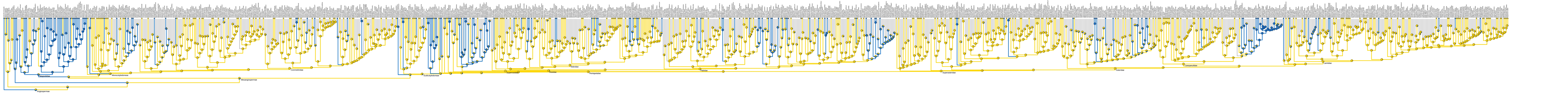

ML ancestral state reconstruction using rayDISC (R:corHMM)
330_A. Androecium structural phyllotaxy (binary) (D2d), ARD model

- whorled
- spiral

Node	ML state	Prob
Angiospermae	spiral	1
Mesangiospermae	spiral	0.9983
Magnoliidae	spiral	0.9972
Monocotyledoneae	whorled	0.8679
Eudicotyledoneae	spiral	0.9681
Commelinidae	whorled	1
Pentapetalae	whorled	0.9932
Superasteridae	whorled	1
Asteridae	whorled	1
Lamiidae	whorled	1
Campanulidae	whorled	1
Superrosidae	whorled	1
Rosidae	whorled	1
Malvidae	whorled	1
Fabidae	whorled	1

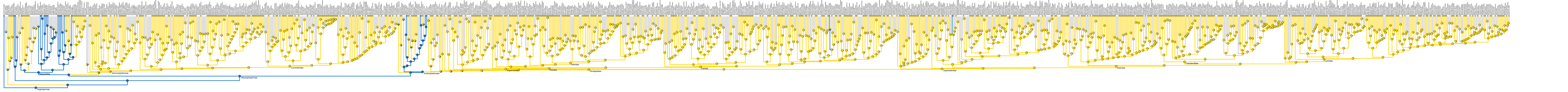

ML ancestral state reconstruction using rayDISC (R:corHMM)
330_A. Androecium structural phyllotaxy (binary) (D2d), ER model

- whorled
- spiral

Node	ML state	Prob
Angiospermae	whorled	0.8773
Mesangiospermae	whorled	0.9998
Magnoliidae	whorled	0.999
Monocotyledoneae	whorled	1
Eudicotyledoneae	whorled	1
Commelinidae	whorled	1
Pentapetalae	whorled	1
Superasteridae	whorled	1
Asteridae	whorled	1
Lamiidae	whorled	1
Campanulidae	whorled	1
Superrosidae	whorled	1
Rosidae	whorled	1
Malvidae	whorled	1
Fabidae	whorled	1

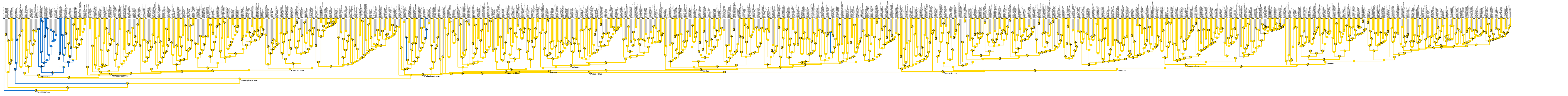

ML ancestral state reconstruction using rayDISC (R:corHMM)
331_A. Number of androecium structural whorls (3−state) (D2c), ARD model

- one (1)
- two (2)
- more than two (>2)

Node	ML state	Prob
Angiospermae	more than two (>2)	0.9959
Mesangiospermae	more than two (>2)	0.9928
Magnoliidae	more than two (>2)	0.994
Monocotyledoneae	two (2)	0.5811
Eudicotyledoneae	more than two (>2)	0.9381
Commelinidae	two (2)	0.9999
Pentapetalae	two (2)	0.9654
Superasteridae	two (2)	0.9999
Asteridae	two (2)	0.9999
Lamiidae	one (1)	0.9996
Campanulidae	one (1)	0.9939
Superrosidae	two (2)	0.9998
Rosidae	two (2)	0.9999
Malvidae	two (2)	1
Fabidae	two (2)	1

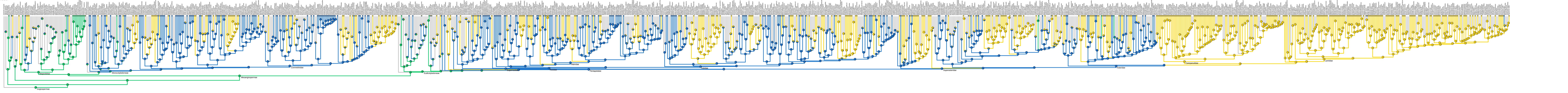

ML ancestral state reconstruction using rayDISC (R:corHMM)
331_A. Number of androecium structural whorls (3−state) (D2c), ER model

- one (1)
- two (2)
- more than two (>2)

Node	ML state	Prob
Angiospermae	one (1)	0.4483
Mesangiospermae	two (2)	0.4983
Magnoliidae	one (1)	0.5677
Monocotyledoneae	two (2)	0.978
Eudicotyledoneae	one (1)	0.5108
Commelinidae	two (2)	0.9999
Pentapetalae	one (1)	0.5998
Superasteridae	one (1)	0.9883
Asteridae	one (1)	0.9998
Lamiidae	one (1)	1
Campanulidae	one (1)	1
Superrosidae	two (2)	0.5587
Rosidae	two (2)	0.6023
Malvidae	two (2)	0.9949
Fabidae	two (2)	0.9746

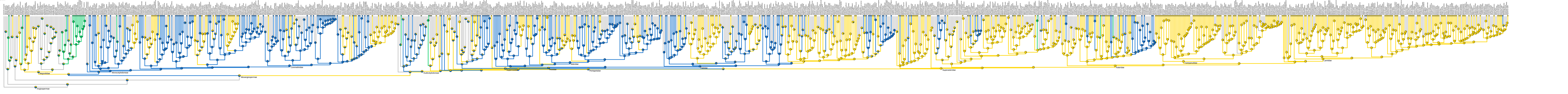

ML ancestral state reconstruction using rayDISC (R:corHMM)
332_A. Androecium structural merism (4−state) (D2c), ARD model

- dimerous
- trimerous
- tetramerous
- pentamerous

Node	ML state	Prob
Angiospermae	trimerous	0.5263
Mesangiospermae	trimerous	0.576
Magnoliidae	trimerous	0.9672
Monocotyledoneae	trimerous	0.9959
Eudicotyledoneae	dimerous	0.9205
Commelinidae	trimerous	1
Pentapetalae	tetramerous	0.6439
Superasteridae	tetramerous	0.9993
Asteridae	tetramerous	0.9984
Lamiidae	tetramerous	0.9734
Campanulidae	tetramerous	0.8477
Superrosidae	tetramerous	0.9992
Rosidae	tetramerous	0.9992
Malvidae	tetramerous	0.9955
Fabidae	tetramerous	0.9757

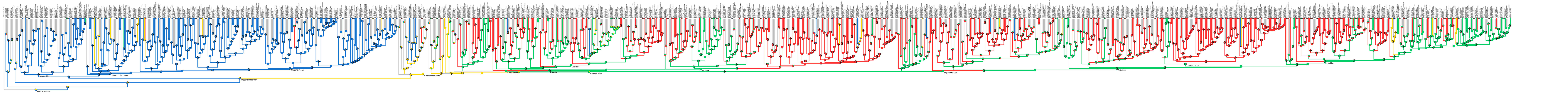

ML ancestral state reconstruction using rayDISC (R:corHMM)
332_A. Androecium structural merism (4−state) (D2c), ER model

- dimerous
- trimerous
- tetramerous
- pentamerous

Node	ML state	Prob
Angiospermae	trimerous	0.818
Mesangiospermae	trimerous	0.886
Magnoliidae	trimerous	0.9995
Monocotyledoneae	trimerous	0.9999
Eudicotyledoneae	trimerous	0.5462
Commelinidae	trimerous	1
Pentapetalae	pentamerous	0.9593
Superasteridae	pentamerous	1
Asteridae	pentamerous	0.9997
Lamiidae	pentamerous	1
Campanulidae	pentamerous	1
Superrosidae	pentamerous	1
Rosidae	pentamerous	1
Malvidae	pentamerous	1
Fabidae	pentamerous	1

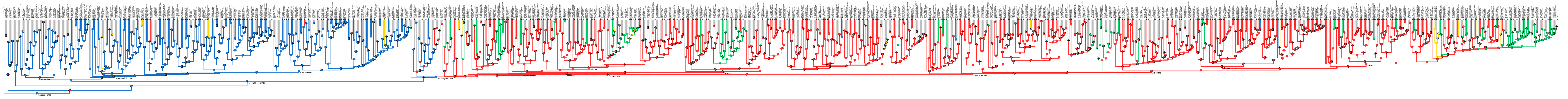

ML ancestral state reconstruction using rayDISC (R:corHMM)
400_A. Gynoecium phyllotaxy (D2d), ARD model

- whorled
- spiral

Node	ML state	Prob
Angiospermae	spiral	1
Mesangiospermae	spiral	0.999
Magnoliidae	spiral	0.9983
Monocotyledoneae	whorled	0.8219
Eudicotyledoneae	spiral	0.9771
Commelinidae	whorled	1
Pentapetalae	whorled	0.9945
Superasteridae	whorled	1
Asteridae	whorled	1
Lamiidae	whorled	1
Campanulidae	whorled	1
Superrosidae	whorled	1
Rosidae	whorled	1
Malvidae	whorled	1
Fabidae	whorled	1

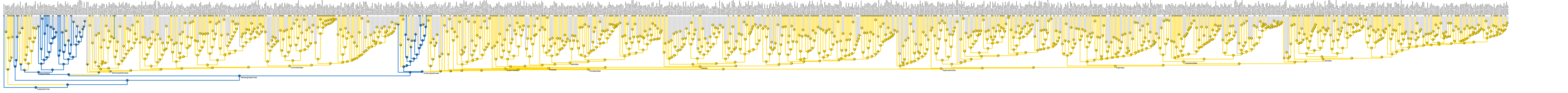

ML ancestral state reconstruction using rayDISC (R:corHMM)
400_A. Gynoecium phyllotaxy (D2d), ER model

whorled
spiral

Node	ML state	Prob
Angiospermae	whorled	0.8767
Mesangiospermae	whorled	0.9998
Magnoliidae	whorled	0.9988
Monocotyledoneae	whorled	1
Eudicotyledoneae	whorled	1
Commelinidae	whorled	1
Pentapetalae	whorled	1
Superasteridae	whorled	1
Asteridae	whorled	1
Lamiidae	whorled	1
Campanulidae	whorled	1
Superrosidae	whorled	1
Rosidae	whorled	1
Malvidae	whorled	1
Fabidae	whorled	1

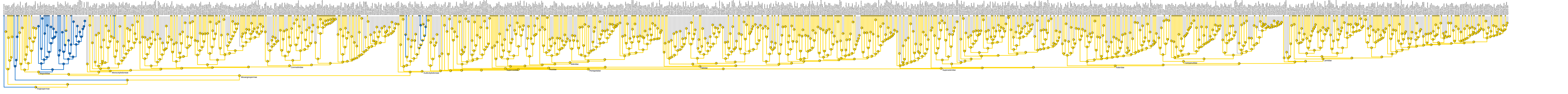

ML ancestral state reconstruction using rayDISC (R:corHMM)
401_B. Number of structural carpels (5−state) (D2c), ARD model

- one (1)
- two (2)
- three (3)
- four or five (4−5)
- more than five (>5)

Node	ML state	Prob
Angiospermae	more than five (>5)	0.9934
Mesangiospermae	more than five (>5)	0.9998
Magnoliidae	more than five (>5)	0.9995
Monocotyledoneae	more than five (>5)	0.8429
Eudicotyledoneae	more than five (>5)	0.9917
Commelinidae	three (3)	1
Pentapetalae	four or five (4−5)	0.9729
Superasteridae	four or five (4−5)	0.9999
Asteridae	four or five (4−5)	0.9971
Lamiidae	two (2)	0.9878
Campanulidae	four or five (4−5)	0.6447
Superrosidae	four or five (4−5)	1
Rosidae	four or five (4−5)	1
Malvidae	four or five (4−5)	1
Fabidae	four or five (4−5)	1

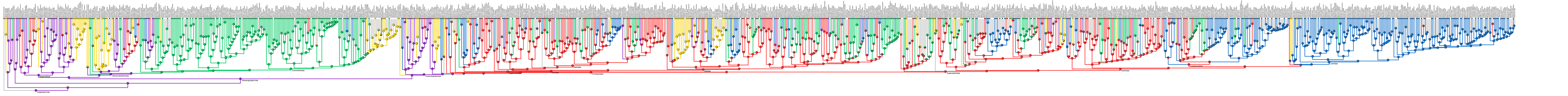

ML ancestral state reconstruction using rayDISC (R:corHMM)
401_B. Number of structural carpels (5−state) (D2c), ER model

- one (1)
- two (2)
- three (3)
- four or five (4−5)
- more than five (>5)

Node	ML state	Prob
Angiospermae	more than five (>5)	0.973
Mesangiospermae	more than five (>5)	0.9946
Magnoliidae	more than five (>5)	0.9968
Monocotyledoneae	three (3)	0.8783
Eudicotyledoneae	more than five (>5)	0.9694
Commelinidae	three (3)	1
Pentapetalae	four or five (4−5)	0.7109
Superasteridae	three (3)	0.5978
Asteridae	two (2)	0.602
Lamiidae	two (2)	1
Campanulidae	two (2)	0.9993
Superrosidae	four or five (4−5)	0.7667
Rosidae	four or five (4−5)	0.7875
Malvidae	four or five (4−5)	0.9445
Fabidae	four or five (4−5)	0.9368

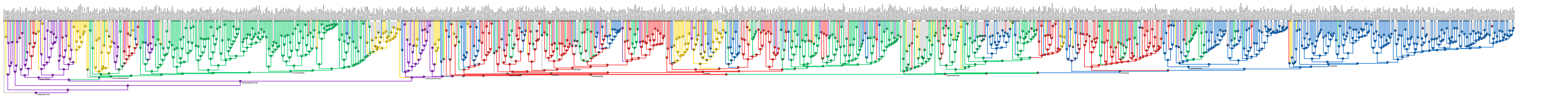

ML ancestral state reconstruction using rayDISC (R:corHMM)
403_A. Fusion of ovaries (binary) (D2c), ARD model

free (<5%)
fused (>5%)

Node	ML state	Prob
Angiospermae	free (<5%)	0.9997
Mesangiospermae	free (<5%)	0.9999
Magnoliidae	free (<5%)	0.9991
Monocotyledoneae	free (<5%)	0.8232
Eudicotyledoneae	free (<5%)	0.999
Commelinidae	fused (>5%)	1
Pentapetalae	fused (>5%)	0.8394
Superasteridae	fused (>5%)	0.9999
Asteridae	fused (>5%)	1
Lamiidae	fused (>5%)	1
Campanulidae	fused (>5%)	1
Superrosidae	fused (>5%)	0.9998
Rosidae	fused (>5%)	0.9999
Malvidae	fused (>5%)	1
Fabidae	fused (>5%)	1

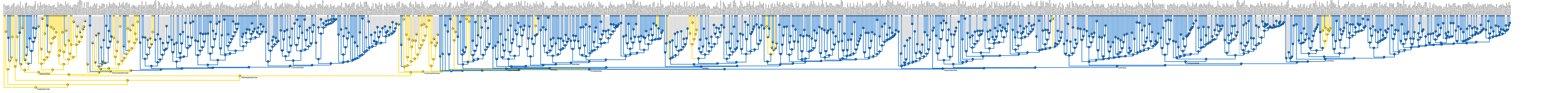

ML ancestral state reconstruction using rayDISC (R:corHMM)
403_A. Fusion of ovaries (binary) (D2c), ER model

- free (<5%)
- fused (>5%)

Node	ML state	Prob
Angiospermae	free (<5%)	0.7598
Mesangiospermae	fused (>5%)	0.8154
Magnoliidae	fused (>5%)	0.8683
Monocotyledoneae	fused (>5%)	0.9958
Eudicotyledoneae	fused (>5%)	0.8175
Commelinidae	fused (>5%)	1
Pentapetalae	fused (>5%)	1
Superasteridae	fused (>5%)	1
Asteridae	fused (>5%)	1
Lamiidae	fused (>5%)	1
Campanulidae	fused (>5%)	1
Superrosidae	fused (>5%)	1
Rosidae	fused (>5%)	1
Malvidae	fused (>5%)	1
Fabidae	fused (>5%)	1

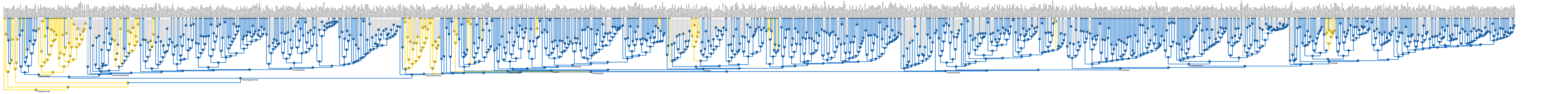

ML ancestral state reconstruction using rayDISC (R:corHMM)
404_B. Style differentiation (D2d), ARD model

absent
present

Node	ML state	Prob
Angiospermae	absent	0.9971
Mesangiospermae	absent	0.9945
Magnoliidae	absent	0.9996
Monocotyledoneae	absent	0.9745
Eudicotyledoneae	present	0.6244
Commelinidae	present	0.948
Pentapetalae	present	0.9946
Superasteridae	present	0.9999
Asteridae	present	1
Lamiidae	present	0.9998
Campanulidae	present	0.9988
Superrosidae	present	0.9996
Rosidae	present	0.9998
Malvidae	present	0.9998
Fabidae	present	1

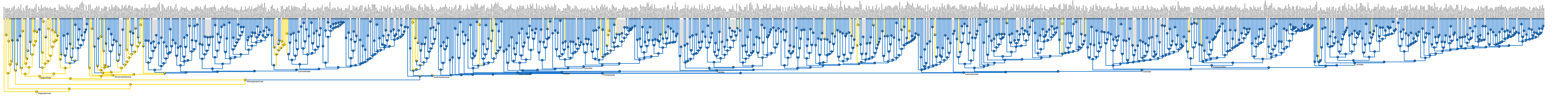

ML ancestral state reconstruction using rayDISC (R:corHMM)
404_B. Style differentiation (D2d), ER model

absent
present

Node	ML state	Prob
Angiospermae	absent	0.5095
Mesangiospermae	present	0.9999
Magnoliidae	present	1
Monocotyledoneae	present	1
Eudicotyledoneae	present	1
Commelinidae	present	1
Pentapetalae	present	1
Superasteridae	present	1
Asteridae	present	1
Lamiidae	present	1
Campanulidae	present	1
Superrosidae	present	1
Rosidae	present	1
Malvidae	present	1
Fabidae	present	1

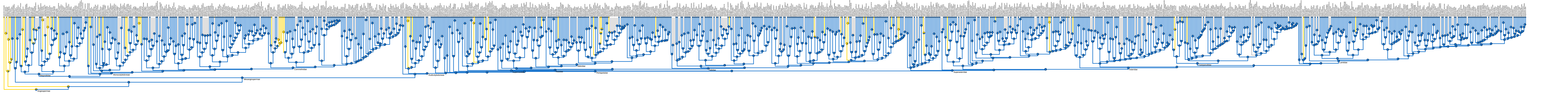

ML ancestral state reconstruction using rayDISC (R:corHMM)
406_A. Fusion of styles (D2c), ARD model

- free (<5%)
- fused (>5%)

Node	ML state	Prob
Angiospermae	free (<5%)	0.9778
Mesangiospermae	free (<5%)	0.9999
Magnoliidae	free (<5%)	0.9993
Monocotyledoneae	free (<5%)	0.9739
Eudicotyledoneae	free (<5%)	1
Commelinidae	fused (>5%)	0.9841
Pentapetalae	free (<5%)	0.9945
Superasteridae	free (<5%)	0.9264
Asteridae	fused (>5%)	0.9156
Lamiidae	fused (>5%)	0.9832
Campanulidae	fused (>5%)	0.9806
Superrosidae	free (<5%)	0.9799
Rosidae	free (<5%)	0.9036
Malvidae	fused (>5%)	0.8969
Fabidae	free (<5%)	0.8748

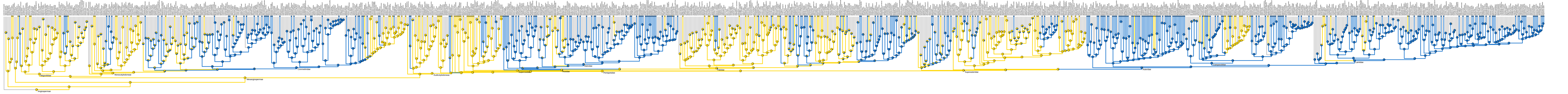

ML ancestral state reconstruction using rayDISC (R:corHMM)
406_A. Fusion of styles (D2c), ER model

- free (<5%)
- fused (>5%)

Node	ML state	Prob
Angiospermae	free (<5%)	0.9567
Mesangiospermae	free (<5%)	0.9819
Magnoliidae	free (<5%)	0.9783
Monocotyledoneae	free (<5%)	0.8618
Eudicotyledoneae	free (<5%)	0.9807
Commelinidae	fused (>5%)	0.9996
Pentapetalae	free (<5%)	0.7406
Superasteridae	fused (>5%)	0.792
Asteridae	fused (>5%)	0.9911
Lamiidae	fused (>5%)	0.9982
Campanulidae	fused (>5%)	0.9995
Superrosidae	fused (>5%)	0.7095
Rosidae	fused (>5%)	0.8889
Malvidae	fused (>5%)	0.9995
Fabidae	fused (>5%)	0.9372

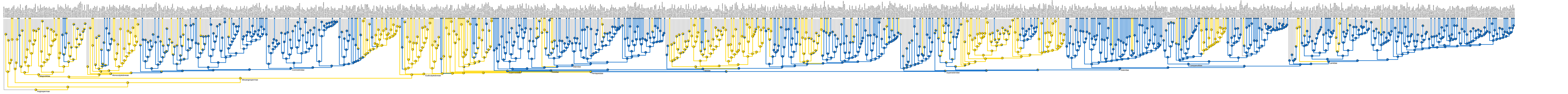

ML ancestral state reconstruction using rayDISC (R:corHMM)
411_A. Number of ovules per functional carpel (3−state) (D2c), ARD model

- one (1)
- two (2)
- three or more (>2)

Node	ML state	Prob
Angiospermae	three or more (>2)	0.9743
Mesangiospermae	three or more (>2)	1
Magnoliidae	three or more (>2)	0.9979
Monocotyledoneae	three or more (>2)	0.9999
Eudicotyledoneae	three or more (>2)	0.9998
Commelinidae	three or more (>2)	0.9469
Pentapetalae	three or more (>2)	1
Superasteridae	three or more (>2)	1
Asteridae	three or more (>2)	0.9998
Lamiidae	three or more (>2)	0.9991
Campanulidae	three or more (>2)	0.9997
Superrosidae	three or more (>2)	1
Rosidae	three or more (>2)	0.9999
Malvidae	three or more (>2)	1
Fabidae	three or more (>2)	0.999

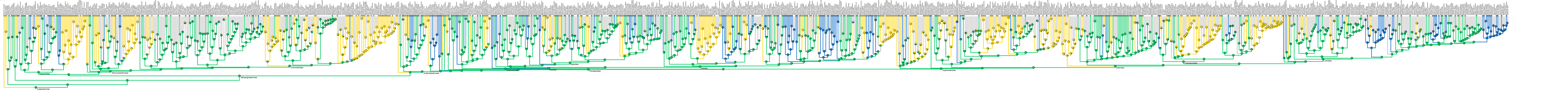

ML ancestral state reconstruction using rayDISC (R:corHMM)
411_A. Number of ovules per functional carpel (3−state) (D2c), ER model

- one (1)
- two (2)
- three or more (>2)

Node	ML state	Prob
Angiospermae	one (1)	0.8172
Mesangiospermae	one (1)	0.8488
Magnoliidae	one (1)	0.8165
Monocotyledoneae	one (1)	0.8365
Eudicotyledoneae	one (1)	0.8241
Commelinidae	one (1)	0.9495
Pentapetalae	one (1)	0.8052
Superasteridae	one (1)	0.9592
Asteridae	one (1)	0.9715
Lamiidae	one (1)	0.5193
Campanulidae	one (1)	0.9733
Superrosidae	three or more (>2)	0.4708
Rosidae	three or more (>2)	0.483
Malvidae	three or more (>2)	0.7999
Fabidae	two (2)	0.5256

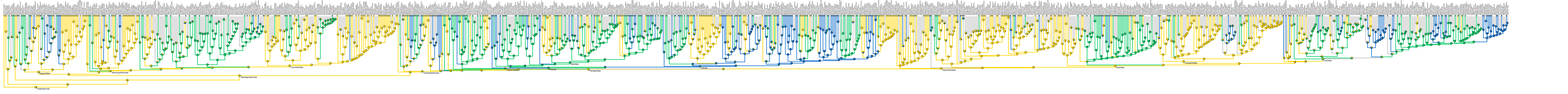

ML ancestral state reconstruction using rayDISC (R:corHMM)
412_A. Placentation (D2d), ARD model

- axile
- parietal
- free−central
- laminar

Node	ML state	Prob
Angiospermae	laminar	0.921
Mesangiospermae	axile	0.992
Magnoliidae	axile	0.9788
Monocotyledoneae	axile	0.9979
Eudicotyledoneae	axile	0.994
Commelinidae	axile	1
Pentapetalae	axile	1
Superasteridae	axile	0.9999
Asteridae	axile	1
Lamiidae	axile	0.9998
Campanulidae	axile	1
Superrosidae	axile	1
Rosidae	axile	1
Malvidae	axile	1
Fabidae	axile	0.9996

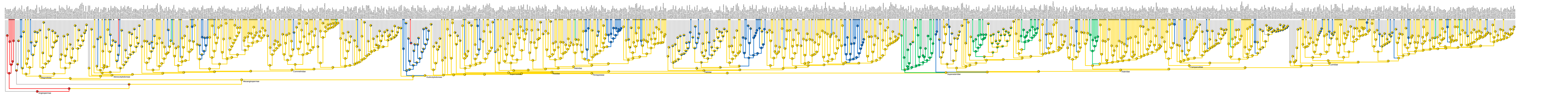

ML ancestral state reconstruction using rayDISC (R:corHMM)
412_A. Placentation (D2d), ER model

- axile
- parietal
- free−central
- laminar

Node	ML state	Prob
Angiospermae	axile	0.7081
Mesangiospermae	axile	0.9983
Magnoliidae	axile	0.9888
Monocotyledoneae	axile	0.9996
Eudicotyledoneae	axile	0.9988
Commelinidae	axile	1
Pentapetalae	axile	1
Superasteridae	axile	0.9997
Asteridae	axile	1
Lamiidae	axile	0.9999
Campanulidae	axile	1
Superrosidae	axile	1
Rosidae	axile	1
Malvidae	axile	1
Fabidae	axile	0.9997

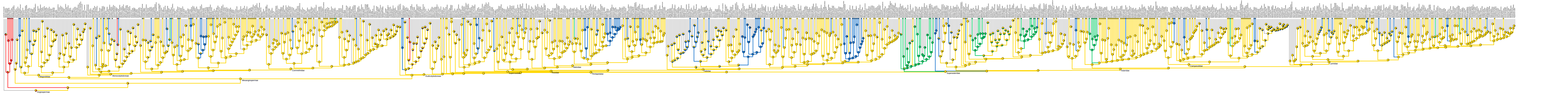

ML ancestral state reconstruction using rayDISC (R:corHMM)
5000_A. Number of apertures (D2c), ARD model

- inaperturate
- monoaperturate
- diaperturate
- triaperturate
- polyaperturate

Node	ML state	Prob
Angiospermae	diaperturate	0.9984
Mesangiospermae	diaperturate	0.9989
Magnoliidae	diaperturate	0.9931
Monocotyledoneae	diaperturate	1
Eudicotyledoneae	triaperturate	0.9951
Commelinidae	diaperturate	0.973
Pentapetalae	triaperturate	1
Superasteridae	triaperturate	1
Asteridae	triaperturate	1
Lamiidae	triaperturate	0.9999
Campanulidae	triaperturate	1
Superrosidae	triaperturate	1
Rosidae	triaperturate	1
Malvidae	triaperturate	1
Fabidae	triaperturate	1

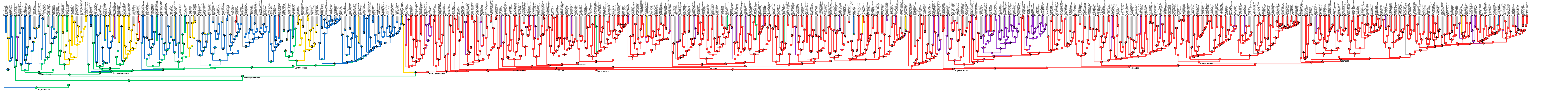

ML ancestral state reconstruction using rayDISC (R:corHMM)
5000_A. Number of apertures (D2c), ER model

- inaperturate
- monoaperturate
- diaperturate
- triaperturate
- polyaperturate

Node	ML state	Prob
Angiospermae	monoaperturate	0.9973
Mesangiospermae	monoaperturate	0.9969
Magnoliidae	monoaperturate	1
Monocotyledoneae	monoaperturate	1
Eudicotyledoneae	triaperturate	0.9992
Commelinidae	monoaperturate	1
Pentapetalae	triaperturate	1
Superasteridae	triaperturate	1
Asteridae	triaperturate	1
Lamiidae	triaperturate	1
Campanulidae	triaperturate	1
Superrosidae	triaperturate	1
Rosidae	triaperturate	1
Malvidae	triaperturate	1
Fabidae	triaperturate	1

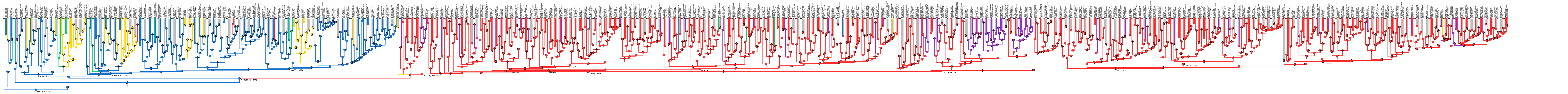

ML ancestral state reconstruction using rayDISC (R:corHMM)
5002_A. Aperture shape (D2d), ARD model

elongate
pore−like
colporate

Node	ML state	Prob
Angiospermae	elongate	0.93
Mesangiospermae	elongate	0.9997
Magnoliidae	elongate	0.9998
Monocotyledoneae	elongate	0.9999
Eudicotyledoneae	elongate	0.9817
Commelinidae	elongate	0.9959
Pentapetalae	colporate	0.8553
Superasteridae	colporate	0.9993
Asteridae	colporate	1
Lamiidae	colporate	0.9999
Campanulidae	colporate	1
Superrosidae	colporate	0.9992
Rosidae	colporate	0.9999
Malvidae	colporate	1
Fabidae	colporate	1

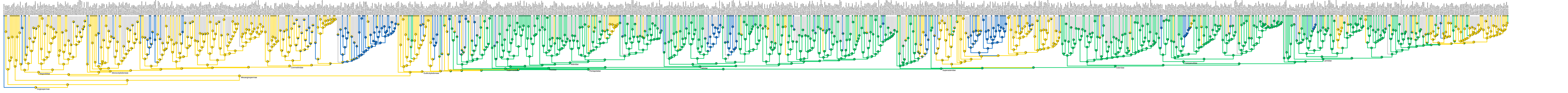

ML ancestral state reconstruction using rayDISC (R:corHMM)
5002_A. Aperture shape (D2d), ER model

- elongate
- pore−like
- colporate

Node	ML state	Prob
Angiospermae	elongate	0.9452
Mesangiospermae	elongate	0.9999
Magnoliidae	elongate	1
Monocotyledoneae	elongate	1
Eudicotyledoneae	elongate	0.9925
Commelinidae	elongate	0.9986
Pentapetalae	colporate	0.7445
Superasteridae	colporate	0.9661
Asteridae	colporate	0.9989
Lamiidae	colporate	0.9999
Campanulidae	colporate	1
Superrosidae	colporate	0.9802
Rosidae	colporate	0.9964
Malvidae	colporate	1
Fabidae	colporate	0.9999

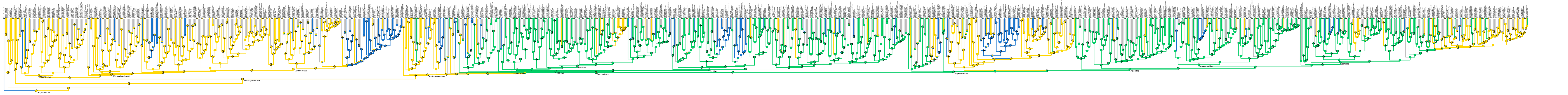

Supplement: Supplementary file 4 — Dataset S4 Ancestral state reconstructions for each character obtained with a maximum likelihood approach. [file NPH-241-1348-s005.pdf]
