# Supplementary material for: Angiosperm flowers reached their highest morphological diversity early in their evolutionary history
Source: New Phytol. 2023 Nov 29;241(3):1348–60. doi: 10.1111/nph.19389 (PMC10952840; doi:10.1111/nph.19389)

***New Phytologist* Supporting Information Data S5**

Article title: Angiosperm flowers reached their highest morphological diversity early in their evolutionary history

Authors: Andrea M. López-Martínez, Maria von Balthazar, Jürg Schönenberger, Susana Magallón, Hervé Sauquet, Marion Chartier

Article acceptance date: 20 October 2023

Content: Ancestral state reconstructions for each character obtained with a stochastic mapping approach.

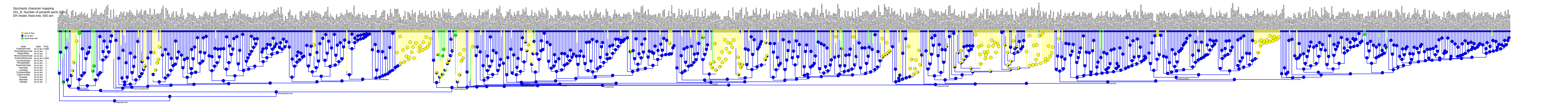
Stochastic character mapping
201_B. Number of perianth parts (D2c)
ER model, fixed tree, 500 sim
one to five
six to ten
more than ten
Node State Prob
Angiospermae six to ten 0.886
Mesangiospermae six to ten 1
Magnoliidae six to ten 1
Monocotyledoneae six to ten 1
Eudicotyledoneae six to ten 0.994
Commelinidae six to ten 1
Pentapetalae six to ten 1
Superasteridae six to ten 1
Asteridae six to ten 1
Lamiidae six to ten 1
Campanulidae six to ten 1
Superrosidae six to ten 1
Rosidae six to ten 1
Malvidae six to ten 1
Fabidae six to ten 1
Magnoliidae

Stochastic character mapping
100_B. Structural sex of flowers (D2d)
ER model, fixed tree, 500 sim

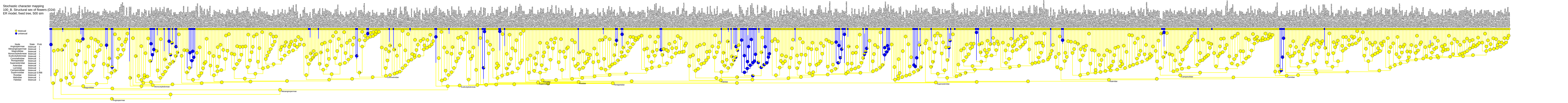

Node	State	Prob
Angiospermae	bisexual	1
Mesangiospermae	bisexual	1
Magnoliidae	bisexual	1
Monocotyledoneae	bisexual	1
Eudicotyledoneae	bisexual	1
Commelinidae	bisexual	1
Pentapetalae	bisexual	1
Superasteridae	bisexual	1
Asteridae	bisexual	1
Lamiidae	bisexual	1
Campanulidae	bisexual	1
Superrosidae	bisexual	0.998
Rosidae	bisexual	1
Malvidae	bisexual	1
Fabidae	bisexual	1

Stochastic character mapping
102_B. Ovary position (binary) (D2d)
ER model, fixed tree, 500 sim

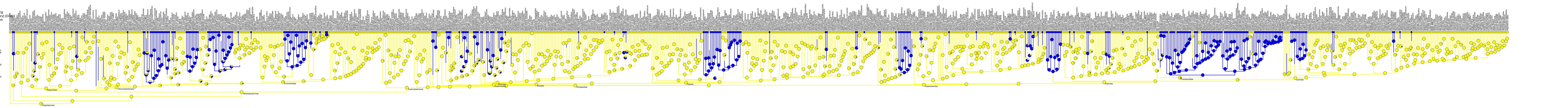

Node	State	Prob
Angiospermae	superior	0.996
Mesangiospermae	superior	1
Magnoliidae	superior	1
Monocotyledoneae	superior	1
Eudicotyledoneae	superior	1
Commelinidae	superior	0.996
Pentapetalae	superior	1
Superasteridae	superior	1
Asteridae	superior	1
Lamiidae	superior	1
Campanulidae	superior	0.894
Superrosidae	superior	1
Rosidae	superior	1
Malvidae	superior	1
Fabidae	superior	1

Stochastic character mapping
201_A. Perianth presence (D2c)
ER model, fixed tree, 500 sim

absent
present

Node	State	Prob
Angiospermae	present	1
Mesangiospermae	present	1
Magnoliidae	present	1
Monocotyledoneae	present	1
Eudicotyledoneae	present	1
Commelinidae	present	1
Pentapetalae	present	1
Superasteridae	present	1
Asteridae	present	1
Lamiidae	present	1
Campanulidae	present	1
Superrosidae	present	1
Rosidae	present	1
Malvidae	present	1
Fabidae	present	1

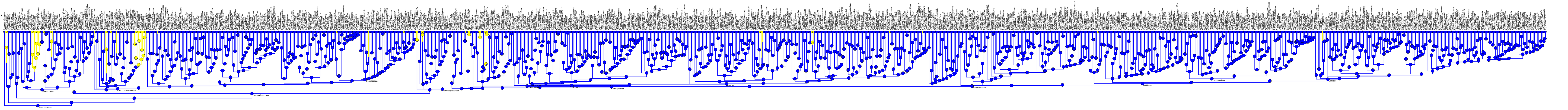

Stochastic character mapping
204_A. Fusion of perianth (D2c)
ER model, fixed tree, 500 sim

free
fused

Node	State	Prob
Angiospermae	free	0.988
Mesangiospermae	free	1
Magnoliidae	free	1
Monocotyledoneae	free	1
Eudicotyledoneae	free	1
Commelinidae	free	0.998
Pentapetalae	free	1
Superasteridae	free	0.974
Asteridae	free	0.836
Lamiidae	fused	0.992
Campanulidae	fused	0.99
Superrosidae	free	1
Rosidae	free	1
Malvidae	free	1
Fabidae	free	1

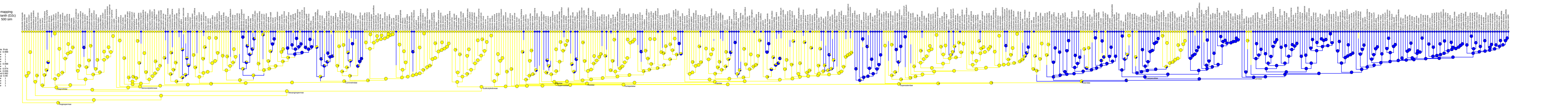

Stochastic character mapping
207_A. Symmetry of perianth (D2d)
ER model, fixed tree, 500 sim

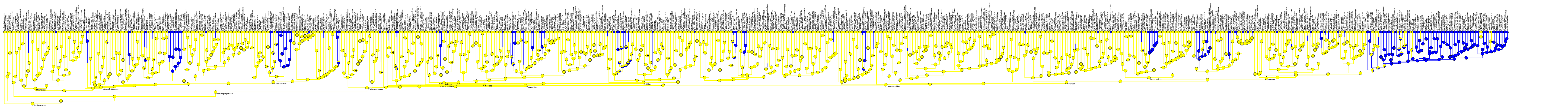

actinomorphic
zygomorphic

Node	State	Prob
Angiospermae	actinomorphic	1
Mesangiospermae	actinomorphic	1
Magnoliidae	actinomorphic	1
Monocotyledoneae	actinomorphic	1
Eudicotyledoneae	actinomorphic	1
Commelinidae	actinomorphic	1
Pentapetalae	actinomorphic	1
Superasteridae	actinomorphic	1
Asteridae	actinomorphic	1
Lamiidae	actinomorphic	1
Campanulidae	actinomorphic	1
Superrosidae	actinomorphic	1
Rosidae	actinomorphic	1
Malvidae	actinomorphic	1
Fabidae	actinomorphic	1

Stochastic character mapping
230_A. Perianth phyllotaxy (D2d)
ER model, fixed tree, 500 sim

- whorled
- spiral

Node	State	Prob
Angiospermae	whorled	0.85
Mesangiospermae	whorled	1
Magnoliidae	whorled	1
Monocotyledoneae	whorled	1
Eudicotyledoneae	whorled	1
Commelinidae	whorled	1
Pentapetalae	whorled	1
Superasteridae	whorled	1
Asteridae	whorled	1
Lamiidae	whorled	1
Campanulidae	whorled	1
Superrosidae	whorled	1
Rosidae	whorled	1
Malvidae	whorled	1
Fabidae	whorled	1

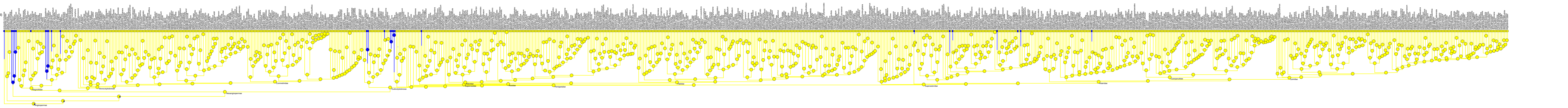

Stochastic character mapping
231_A. Number of perianth whorls (D2c)
ER model, fixed tree, 500 sim

one
two
more than two

Node	State	Prob
Angiospermae	two	0.854
Mesangiospermae	two	0.928
Magnoliidae	two	0.836
Monocotyledoneae	two	0.998
Eudicotyledoneae	two	0.932
Commelinidae	two	1
Pentapetalae	two	1
Superasteridae	two	1
Asteridae	two	1
Lamiidae	two	1
Campanulidae	two	1
Superrosidae	two	1
Rosidae	two	1
Malvidae	two	1
Fabidae	two	1

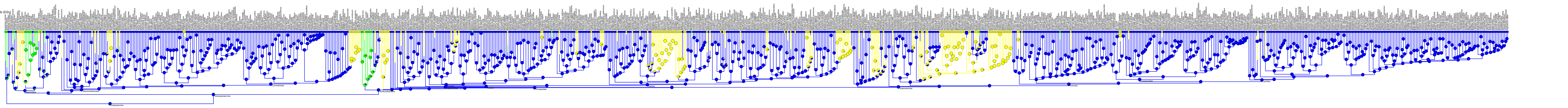

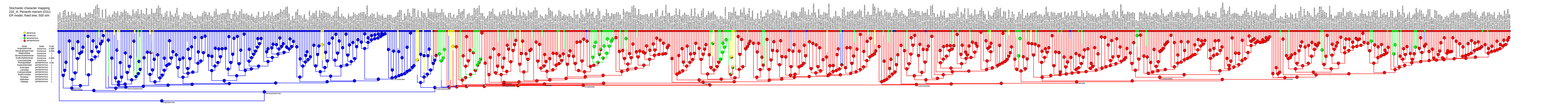
Stochastic character mapping
232_A. Perianth merism (D2c)
ER model, fixed tree, 500 sim
dimerous
trimerous
tetramerous
pentamerous
Node State Prob
Angiospermae trimerous 0.986
Mesangiospermae trimerous 0.994
Magnoliidae trimerous 1
Monocotyledoneae trimerous 1
Eudicotyledoneae trimerous 0.806
Commelinidae trimerous 1
Pentapetalae pentamerous 0.99
Superasteridae pentamerous 1
Asteridae pentamerous 1
Lamiidae pentamerous 1
Campanulidae pentamerous 1
Superrosidae pentamerous 1
Rosidae pentamerous 1
Malvidae pentamerous 1
Fabidae pentamerous 1
Magnoliidae

Stochastic character mapping
234_B. Perianth differentiation (D2d)
ER model, fixed tree, 500 sim

undifferentiated
differentiated

Node	State	Prob
Angiospermae	differentiated	0.946
Mesangiospermae	differentiated	1
Magnoliidae	differentiated	0.998
Monocotyledoneae	differentiated	0.772
Eudicotyledoneae	differentiated	1
Commelinidae	differentiated	0.956
Pentapetalae	differentiated	1
Superasteridae	differentiated	1
Asteridae	differentiated	1
Lamiidae	differentiated	1
Campanulidae	differentiated	1
Superrosidae	differentiated	1
Rosidae	differentiated	1
Malvidae	differentiated	1
Fabidae	differentiated	1

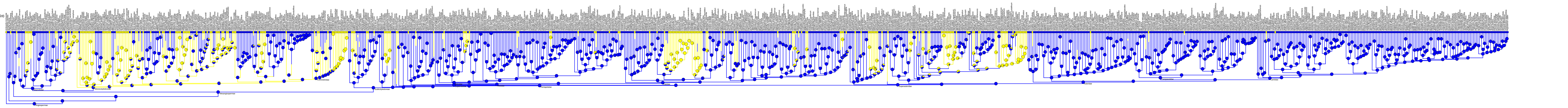

Stochastic character mapping
301_B. Number of fertile stamens (D2c)
ER model, fixed tree, 500 sim

- one to five
- six to ten
- more than ten

Node	State	Prob
Angiospermae	more than ten	0.766
Mesangiospermae	more than ten	0.598
Magnoliidae	more than ten	0.732
Monocotyledoneae	six to ten	0.916
Eudicotyledoneae	one to five	0.898
Commelinidae	six to ten	0.998
Pentapetalae	one to five	0.998
Superasteridae	one to five	1
Asteridae	one to five	1
Lamiidae	one to five	1
Campanulidae	one to five	1
Superrosidae	one to five	0.998
Rosidae	one to five	0.98
Malvidae	six to ten	0.868
Fabidae	one to five	0.752

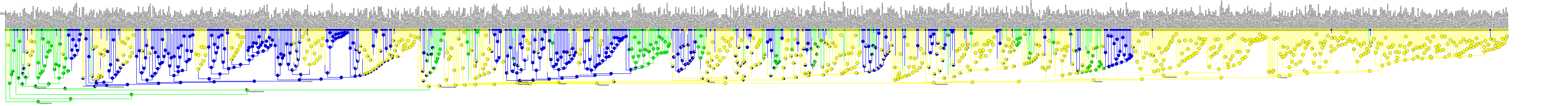

Stochastic character mapping
305_A. Filament (binary) (D2d)
ER model, fixed tree, 500 sim

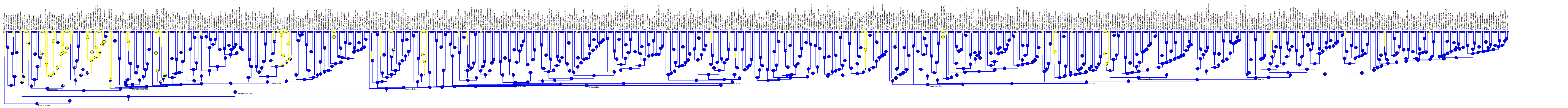

laminar
typical

Node	State	Prob
Angiospermae	typical	0.926
Mesangiospermae	typical	0.99
Magnoliidae	typical	0.92
Monocotyledoneae	typical	0.996
Eudicotyledoneae	typical	1
Commelinidae	typical	1
Pentapetalae	typical	1
Superasteridae	typical	1
Asteridae	typical	1
Lamiidae	typical	1
Campanulidae	typical	0.998
Superrosidae	typical	1
Rosidae	typical	1
Malvidae	typical	1
Fabidae	typical	1

Stochastic character mapping
306_A. Fusion of filaments (D2c)
ER model, fixed tree, 500 sim

free
fused

Node	State	Prob
Angiospermae	free	0.994
Mesangiospermae	free	1
Magnoliidae	free	1
Monocotyledoneae	free	1
Eudicotyledoneae	free	1
Commelinidae	free	1
Pentapetalae	free	1
Superasteridae	free	1
Asteridae	free	1
Lamiidae	free	1
Campanulidae	free	1
Superrosidae	free	1
Rosidae	free	1
Malvidae	free	1
Fabidae	free	1

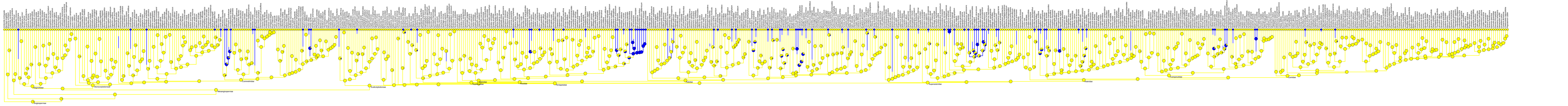

Stochastic character mapping
308_A. Fusion of filaments to inner perianth series (D2c)
ER model, fixed tree, 500 sim

free
fused

Node	State	Prob
Angiospermae	free	0.99
Mesangiospermae	free	1
Magnoliidae	free	1
Monocotyledoneae	free	1
Eudicotyledoneae	free	1
Commelinidae	free	0.882
Pentapetalae	free	1
Superasteridae	free	1
Asteridae	free	0.996
Lamiidae	fused	0.512
Campanulidae	free	0.994
Superrosidae	free	1
Rosidae	free	1
Malvidae	free	1
Fabidae	free	1

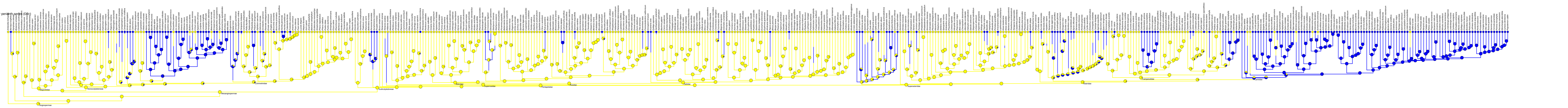

Stochastic character mapping
311_A. Anther orientation (D2d), ER model, fixed tree, 500 sim

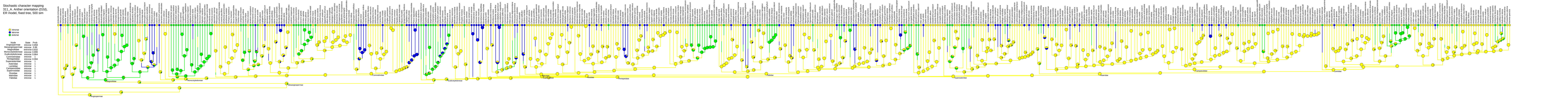

Node	State	Prob
Angiospermae	introrse	0.858
Mesangiospermae	introrse	0.86
Magnoliidae	extrorse	0.832
Monocotyledoneae	introrse	0.896
Eudicotyledoneae	introrse	0.864
Commelinidae	introrse	1
Pentapetalae	introrse	0.994
Superasteridae	introrse	1
Asteridae	introrse	1
Lamiidae	introrse	1
Campanulidae	introrse	1
Superrosidae	introrse	1
Rosidae	introrse	1
Malvidae	introrse	1
Fabidae	introrse	1

Stochastic character mapping
312_A. Anther attachment (binary) (D2d)
ER model, fixed tree, 500 sim

basifixed
dorsifixed

Node	State	Prob
Angiospermae	basifixed	0.692
Mesangiospermae	basifixed	0.69
Magnoliidae	basifixed	0.738
Monocotyledoneae	basifixed	0.956
Eudicotyledoneae	basifixed	0.718
Commelinidae	basifixed	1
Pentapetalae	basifixed	0.774
Superasteridae	basifixed	0.998
Asteridae	basifixed	1
Lamiidae	dorsifixed	1
Campanulidae	dorsifixed	1
Superrosidae	basifixed	0.54
Rosidae	dorsifixed	0.514
Malvidae	dorsifixed	1
Fabidae	dorsifixed	0.97

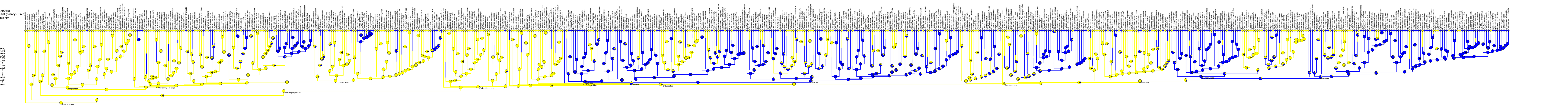

Stochastic character mapping
313_A. Anther dehiscence (D2d)
ER model, fixed tree, 500 sim

whorled
spiral

Node	State	Prob
Angiospermae	longitudinal slit	1
Mesangiospermae	longitudinal slit	1
Magnoliidae	longitudinal slit	1
Monocotyledoneae	longitudinal slit	1
Eudicotyledoneae	longitudinal slit	1
Commelinidae	longitudinal slit	1
Pentapetalae	longitudinal slit	1
Superasteridae	longitudinal slit	1
Asteridae	longitudinal slit	1
Lamiidae	longitudinal slit	1
Campanulidae	longitudinal slit	1
Superrosidae	longitudinal slit	1
Rosidae	longitudinal slit	1
Malvidae	longitudinal slit	1
Fabidae	longitudinal slit	1

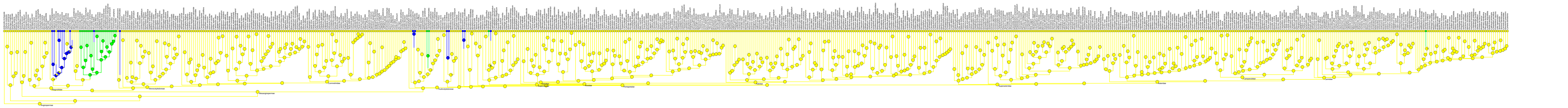

Stochastic character mapping
314_A. Connective extension (apical) (D2d)
ER model, fixed tree, 500 sim

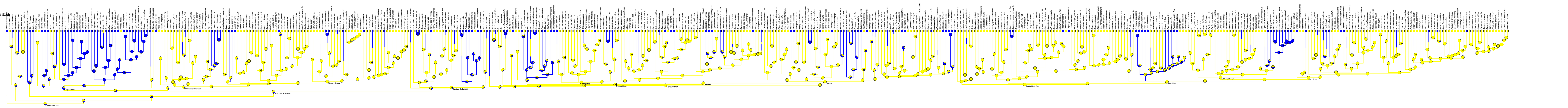

absent
present

Node	State	Prob
Angiospermae	absent	0.628
Mesangiospermae	absent	0.72
Magnoliidae	present	0.638
Monocotyledoneae	absent	0.906
Eudicotyledoneae	absent	0.836
Commelinidae	absent	1
Pentapetalae	absent	0.982
Superasteridae	absent	1
Asteridae	absent	0.998
Lamiidae	absent	1
Campanulidae	absent	1
Superrosidae	absent	0.994
Rosidae	absent	1
Malvidae	absent	1
Fabidae	absent	1

Stochastic character mapping
330_A. Androecium structural phyllotaxy (D2d)
ER model, fixed tree, 500 sim

whorled
spiral

Node	State	Prob
Angiospermae	whorled	0.89
Mesangiospermae	whorled	1
Magnoliidae	whorled	0.998
Monocotyledoneae	whorled	1
Eudicotyledoneae	whorled	1
Commelinidae	whorled	1
Pentapetalae	whorled	1
Superasteridae	whorled	1
Asteridae	whorled	1
Lamiidae	whorled	1
Campanulidae	whorled	1
Superrosidae	whorled	1
Rosidae	whorled	1
Malvidae	whorled	1
Fabidae	whorled	1

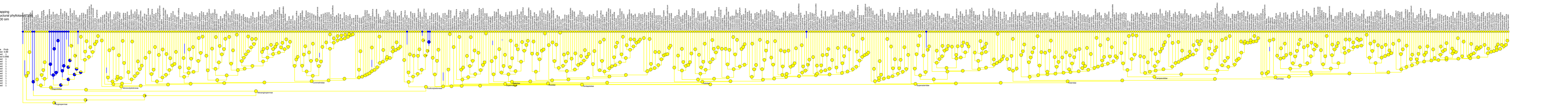

Stochastic character mapping
331_A. Number of androecium structural whorls (D2c)
ER model, fixed tree, 500 sim

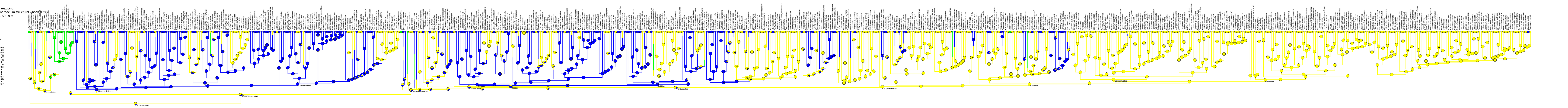

Node	State	Prob
Angiospermae	one	0.692
Mesangiospermae	one	0.69
Magnoliidae	one	0.738
Monocotyledoneae	two	0.956
Eudicotyledoneae	one	0.718
Commelinidae	two	1
Pentapetalae	one	0.774
Superasteridae	one	0.998
Asteridae	one	1
Lamiidae	one	1
Campanulidae	one	1
Superrosidae	one	0.54
Rosidae	two	0.514
Malvidae	two	1
Fabidae	two	0.97

Stochastic character mapping
332_A. Androecium structural merism (D2c)
ER model, fixed tree, 500 sim

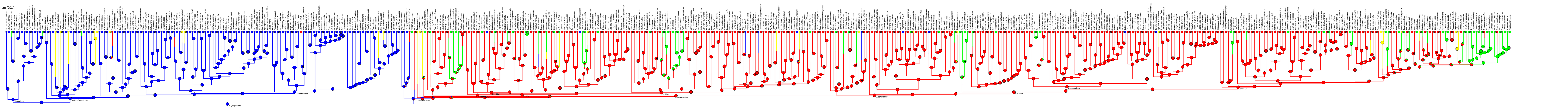

Node	State	Prob
Angiospermae	trimerous	0.94
Mesangiospermae	trimerous	0.94
Magnoliidae	trimerous	1
Monocotyledoneae	trimerous	1
Eudicotyledoneae	trimerous	0.776
Commelinidae	trimerous	1
Pentapetalae	pentamerous	1
Superasteridae	pentamerous	1
Asteridae	pentamerous	0.998
Lamiidae	pentamerous	1
Campanulidae	pentamerous	1
Superrosidae	pentamerous	1
Rosidae	pentamerous	1
Malvidae	pentamerous	1
Fabidae	pentamerous	1

Stochastic character mapping
400_A. Gynoecium phyllotaxy (D2d)
ER model, fixed tree, 500 sim

whorled
spiral

Node	State	Prob
Angiospermae	free	0.968
Mesangiospermae	free	0.976
Magnoliidae	free	0.972
Monocotyledoneae	free	0.824
Eudicotyledoneae	free	0.976
Commelinidae	fused	1
Pentapetalae	free	0.718
Superasteridae	fused	0.774
Asteridae	fused	0.988
Lamiidae	fused	0.996
Campanulidae	fused	1
Superrosidae	fused	0.678
Rosidae	fused	0.898
Malvidae	fused	0.998
Fabidae	fused	0.944

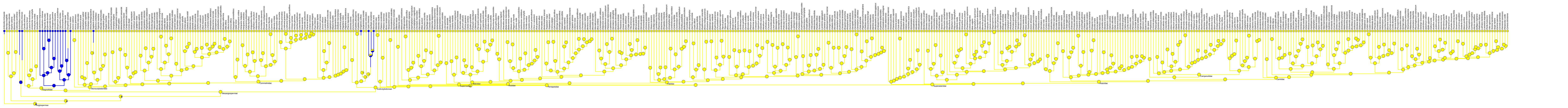

Stochastic character mapping
401_B. Number of structural carpels (D2c)
ER model, fixed tree, 500 sim

- one
- two
- three
- four to five
- more than five

Node	State	Prob
Angiospermae	more than five	0.912
Mesangiospermae	more than five	0.882
Magnoliidae	more than five	0.95
Monocotyledoneae	three	0.79
Eudicotyledoneae	more than five	0.644
Commelinidae	three	1
Pentapetalae	two	0.514
Superasteridae	two	0.522
Asteridae	two	0.786
Lamiidae	two	1
Campanulidae	two	1
Superrosidae	two	0.51
Rosidae	four to five	0.484
Malvidae	four to five	0.994
Fabidae	four to five	0.988

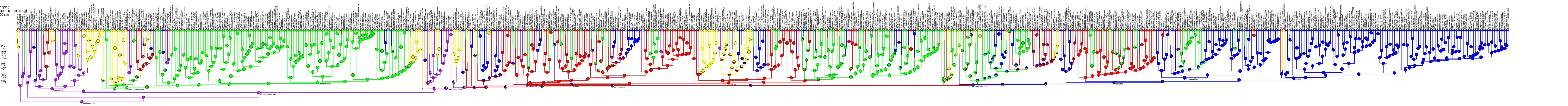

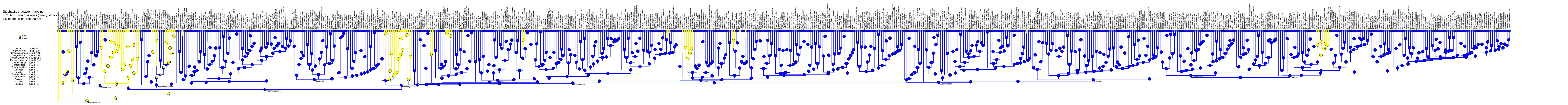
Stochastic character mapping
403_A. Fusion of ovaries (binary) (D2c)
ER model, fixed tree, 500 sim
free
fused
Node State Prob
Angiospermae free 0.77
Mesangiospermae fused 0.81
Magnoliidae fused 0.86
Monocotyledoneae fused 0.996
Eudicotyledoneae fused 0.814
Commelinidae fused 1
Pentapetalae fused 1
Superasteridae fused 1
Asteridae fused 1
Lamiidae fused 1
Campanulidae fused 1
Superrosidae fused 1
Rosidae fused 1
Malvidae fused 1
Fabidae fused 1
Magnoliidae

Stochastic character mapping
404_B. Style differentiation (D2d)
ER model, fixed tree, 500 sim

absent
present

Node	State	Prob
Angiospermae	absent	0.632
Mesangiospermae	present	1
Magnoliidae	present	1
Monocotyledoneae	present	1
Eudicotyledoneae	present	1
Commelinidae	present	1
Pentapetalae	present	1
Superasteridae	present	1
Asteridae	present	1
Lamiidae	present	1
Campanulidae	present	1
Superrosidae	present	1
Rosidae	present	1
Malvidae	present	1
Fabidae	present	1

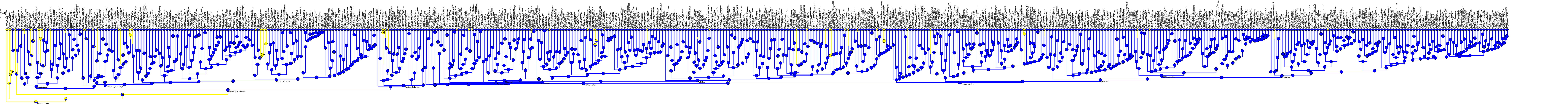

Stochastic character mapping
406_A. Fusion of styles (D2c)
ER model, fixed tree, 500 sim

free
fused

Node	State	Prob
Angiospermae	free	0.968
Mesangiospermae	free	0.976
Magnoliidae	free	0.972
Monocotyledoneae	free	0.824
Eudicotyledoneae	free	0.976
Commelinidae	fused	1
Pentapetalae	free	0.718
Superasteridae	fused	0.774
Asteridae	fused	0.988
Lamiidae	fused	0.996
Campanulidae	fused	1
Superrosidae	fused	0.678
Rosidae	fused	0.898
Malvidae	fused	0.998
Fabidae	fused	0.944

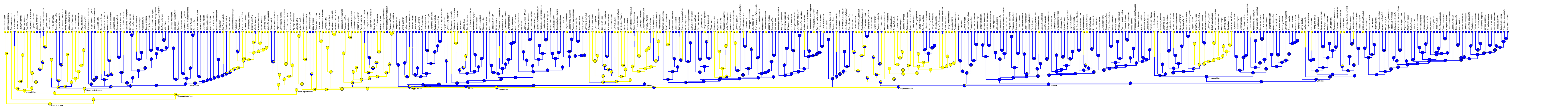

Stochastic character mapping
411_A. Number of ovules per functional carpel (D2c)
ER model, fixed tree, 500 sim

Node	State	Prob
Angiospermae	one	0.79
Mesangiospermae	one	0.82
Magnoliidae	one	0.794
Monocotyledoneae	one	0.796
Eudicotyledoneae	one	0.8
Commelinidae	one	0.922
Pentapetalae	one	0.778
Superasteridae	one	0.948
Asteridae	one	0.952
Lamiidae	one	0.874
Campanulidae	one	0.946
Superrosidae	three or more	0.532
Rosidae	three or more	0.536
Malvidae	three or more	0.682
Fabidae	two	0.484

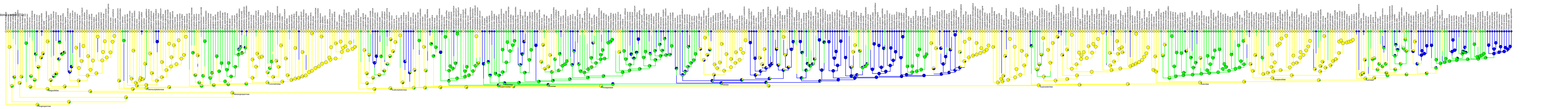

Stochastic character mapping
412_A. Placentation (D2d)
ER model, fixed tree, 500 sim

- axile
- parietal
- free central
- laminar

Node	State	Prob
Angiospermae	axile	0.742
Mesangiospermae	axile	1
Magnoliidae	axile	0.984
Monocotyledoneae	axile	1
Eudicotyledoneae	axile	1
Commelinidae	axile	1
Pentapetalae	axile	1
Superasteridae	axile	1
Asteridae	axile	1
Lamiidae	axile	1
Campanulidae	axile	1
Superrosidae	axile	1
Rosidae	axile	1
Malvidae	axile	1
Fabidae	axile	1

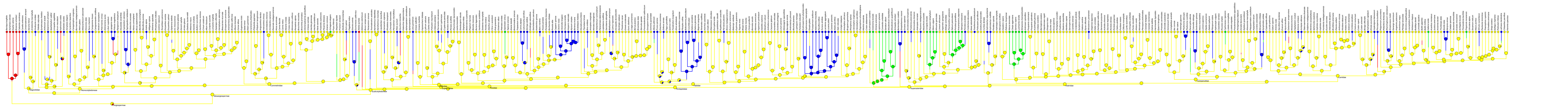

Stochastic character mapping
5000_A. Number of apertures (D2c)
ER model, fixed tree, 500 sim

- inaperturate
- monoaperturate
- diaperturate
- triaperturate
- polyaperturate

Node	State	Prob
Angiospermae	monoaperturate	0.996
Mesangiospermae	monoaperturate	0.996
Magnoliidae	monoaperturate	1
Monocotyledoneae	monoaperturate	1
Eudicotyledoneae	triaperturate	1
Commelinidae	monoaperturate	1
Pentapetalae	triaperturate	1
Superasteridae	triaperturate	1
Asteridae	triaperturate	1
Lamiidae	triaperturate	1
Campanulidae	triaperturate	1
Superrosidae	triaperturate	1
Rosidae	triaperturate	1
Malvidae	triaperturate	1
Fabidae	triaperturate	1

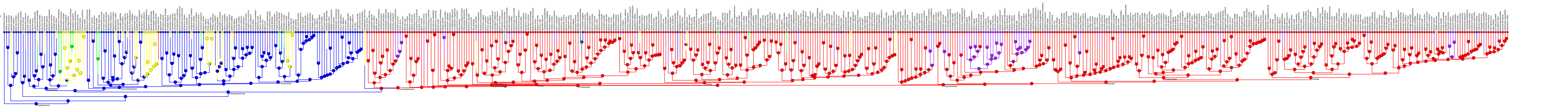

Stochastic character mapping
5002_A. Aperture shape (D2d)
ER model, fixed tree, 500 sim

- elongate
- pore like
- colporate

Node	State	Prob
Angiospermae	elongate	0.96
Mesangiospermae	elongate	0.998
Magnoliidae	elongate	1
Monocotyledoneae	elongate	1
Eudicotyledoneae	elongate	0.994
Commelinidae	elongate	1
Pentapetalae	colporate	0.76
Superasteridae	colporate	0.976
Asteridae	colporate	0.998
Lamiidae	colporate	1
Campanulidae	colporate	1
Superrosidae	colporate	0.988
Rosidae	colporate	1
Malvidae	colporate	1
Fabidae	colporate	1

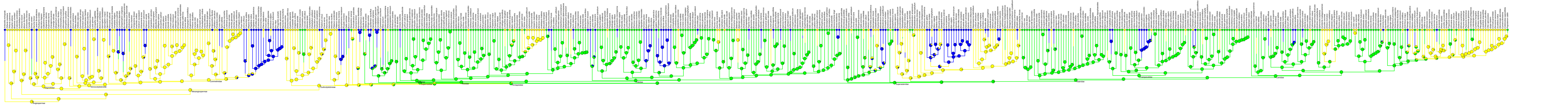

Supplement: Supplementary file 5 — Dataset S5 Ancestral state reconstructions for each character obtained with a stochastic mapping approach. [file NPH-241-1348-s002.pdf]
