# Supplementary material for: Angiosperm flowers reached their highest morphological diversity early in their evolutionary history
Source: New Phytol. 2023 Nov 29;241(3):1348–60. doi: 10.1111/nph.19389 (PMC10952840; doi:10.1111/nph.19389)
Supplement: Supplementary file 6 — Fig. S1 Distribution of missing and nonapplicable data for living species. Fig. S2 Distribution of missing and nonapplicable data for fossil species. Fig. S3 Rarefied floral disparity (meanD and R) through time. Fig. S4 Position of Neogene fossils in the floral morphospace (Fig. 1). Fig. S5 Distribution of character states in the morphospace (Fig. 2). Fig. S6 Position of eccentric species in the morphospace ordination. Fig. S7 Distribution of character states in living Angiosperms. Fig. S8 Rarefied floral disparity (meanD and R) for living angiosperms. Fig. S9 Position of additional living angiosperm clades in the floral morphospace (Fig. 3). Fig. S10 Correlation between sample size and group sizes. Fig. S11 Phylogenetic distribution of species in the four time bins studied and for eccentric species. Methods S1 List of nonapplicable and impossible combinations. Methods S2 Calculation of the mean character difference. Table S1 Divergence time estimates for the 15 ASR. Table S2 Disparity (meanD) for the study angiosperm group. Please note: Wiley is not responsible for the content or functionality of any Supporting Information supplied by the authors. Any queries (other than missing material) should be directed to the New Phytologist Central Office. [file NPH-241-1348-s006.pdf]

New Phytologist

***New Phytologist* Supporting Information**

Article title: Angiosperm flowers reached their highest morphological diversity early in their evolutionary history

Authors: Andrea M. López-Martínez, Maria von Balthazar, Jürg Schönenberger, Susana Magallón, Hervé Sauquet, Marion Chartier

Article acceptance date: 20 October 2023

Contents of this document:

Fig. S1 Distribution of missing and non-applicable data for living species 2
Fig. S2 Distribution of missing and non-applicable data for fossil species 3
Fig. S3 Rarefied floral disparity (*meanD* and *R*) through time 4
Fig. S4 Position of Neogene fossils in the floral morphospace (Fig. 1) 5
Fig. S5 Distribution of character states in the morphospace (Fig. 2) 6
Fig. S4 Position of eccentric species in the morphospace ordination. 13
Fig. S7 Distribution of character states in living Angiosperms 15
Fig. S8 Rarefied floral disparity (*meanD* and *R*) for living angiosperms 16
Fig. S9 Position of additional living angiosperm clades in the floral morphospace (Fig. 3) 17
Fig. S10 Correlation between sample size and groups sizes 18
Fig. S11 Phylogenetic distribution of species in the four time bins studied and for eccentric species 19
Methods S1 List of non-applicable and impossible combinations. 20
Methods S2 Calculation of the mean character difference 21
Table S1 Divergence times estimates for the 15 ASR 22
Table S2 Disparity (*meanD*) for the study angiosperm group 23

Also available as separate files:

Data S1. Script used to generate the distance matrix, the ordination of the space, and calculate D.
Data S2. Morphological dataset, information about scored fossils and about study groups.
Data S3. Proteus extraction used to generate the dataset, containing all references.
Data S4. Ancestral state reconstructions for each character obtained with a maximum likelihood approach.
Data S5. Ancestral state reconstructions for each character obtained with a stochastic mapping approach.

Fig. S1 Distribution of missing and non-applicable data for living species

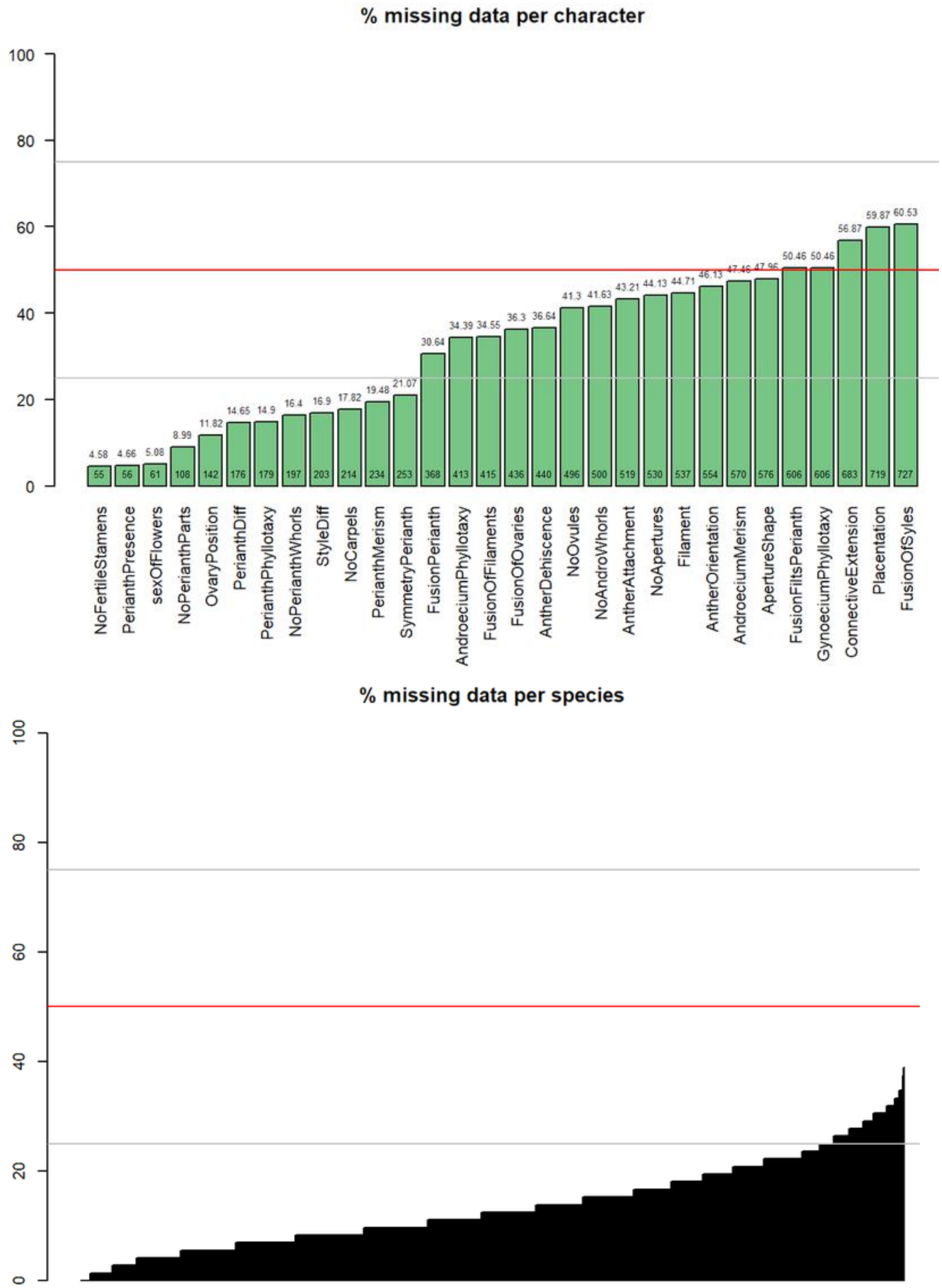

Distribution of missing and non-applicable data per character (top) and species (bottom) for the 1201 living species in our dataset. On top: number at the bottom of the barplots = number of missing entries, on top of barplot: % missing entries per character.

Fig. S2 Distribution of missing and non-applicable data for fossil species

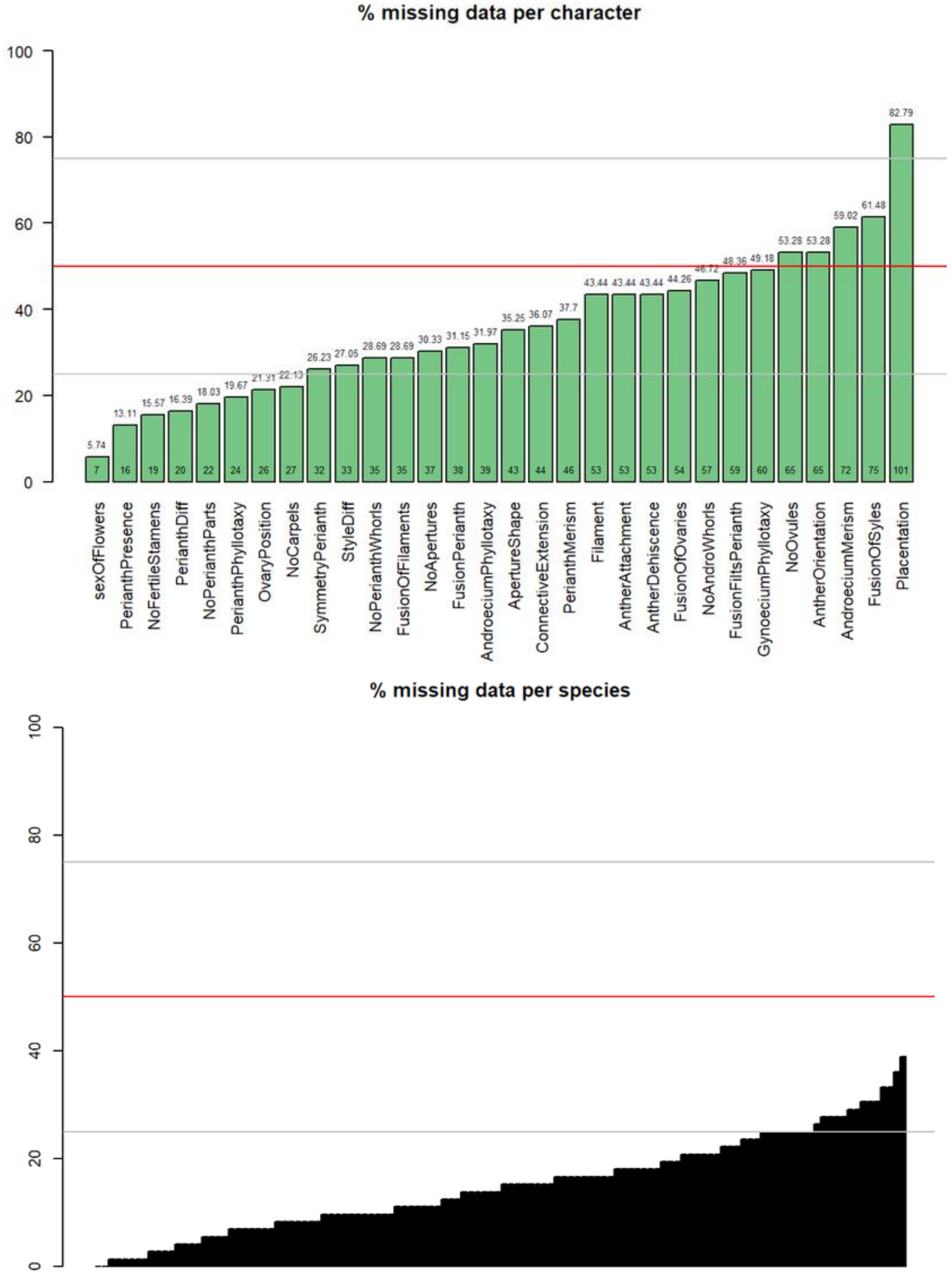

Distribution of missing and non-applicable data per character (top) and species (bottom) for the 121 fossil species in our dataset. On top: number at the bottom of the barplots = number of missing entries, on top of barplot: % missing entries per character.

Fig. S3 Rarefied floral disparity (*meanD* and *R*) through time

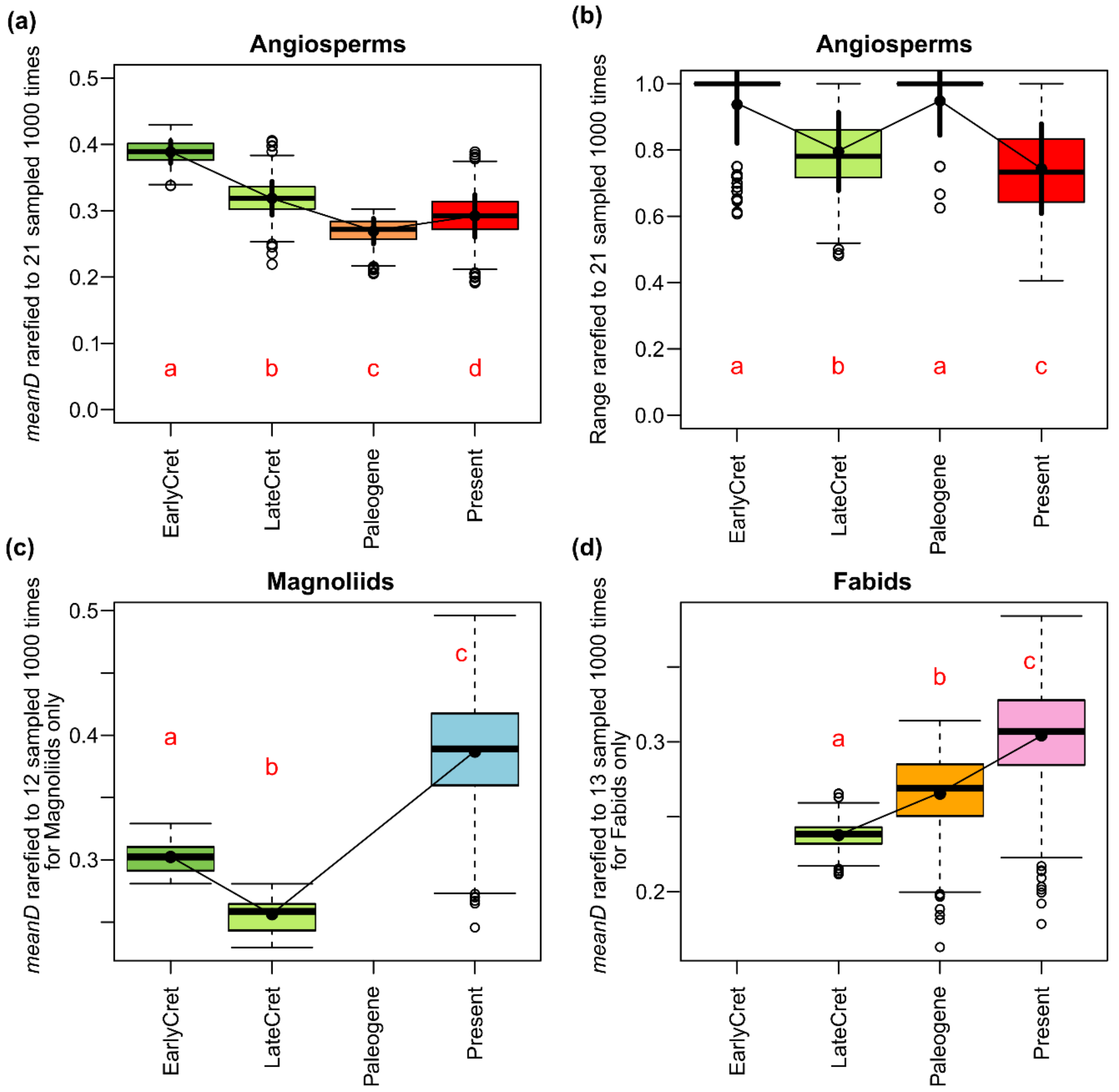

(**a**) and (**b**): rarefied *meanD* and rarefied *R* for the four study time bins. In **(a)**: Kruskal-Wallis chi-squared = 2920.7, df = 3, p-value < 2.2e-16; in **(b)**: chi-squared = 1517, df = 3, p-value < 2.2e-16. (**c**) and (**d**): rarefied *meanD* calculated for three different time bins for either a random sampling (without replacement, and with 1000 repetitions) of 12 magnoliids (**c**) or 13 fabids (**d**) only. In **(c)**: Kruskal-Wallis chi-squared = 2558.2, df = 2, p-value < 2.2e-16; in **(b)**: chi-squared = 1730.5, df = 2, p-value < 2.2e-16.
Black dots = disparity (*meanD*) for each time bin. SD = standard deviation (black error bars). Boxplots = distribution of D for each group. Red letters indicate post hoc test results, groups with a different letter significantly differ from each other.
Note that results displayed in (**c**) and (**d**) are based on a character set designed to study angiosperms as a whole and that proper studies should be perform for each subclade, using character sets tuned for these clades, to properly understand their evolution.

Fig. S4 Position of Neogene fossils in the floral morphospace (Fig. 1)

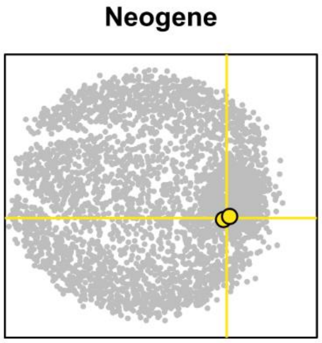

Fossils from the Neogene were excluded from the statistical analyses as they contained only two species in our sampling. They were anyway included in the morphospace presented in Fig. 1.

Fig. S5 Distribution of character states in the morphospace (Fig. 2)

The distribution of these character states is quasi identical for the three morphospaces presented in this study (Fig. 1, Fig. 2 and Fig. 3), therefore we only display them for Fig. 2.

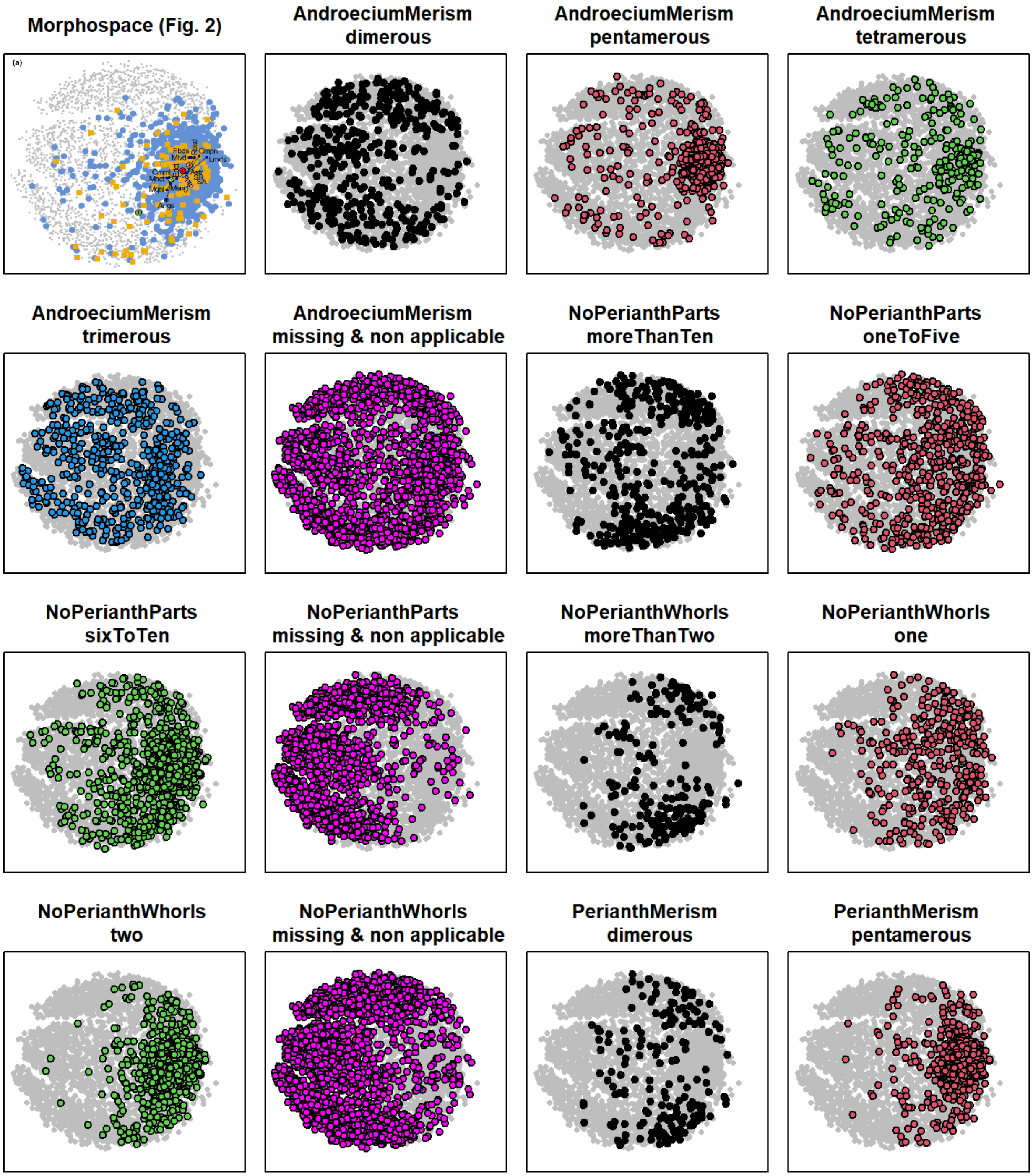

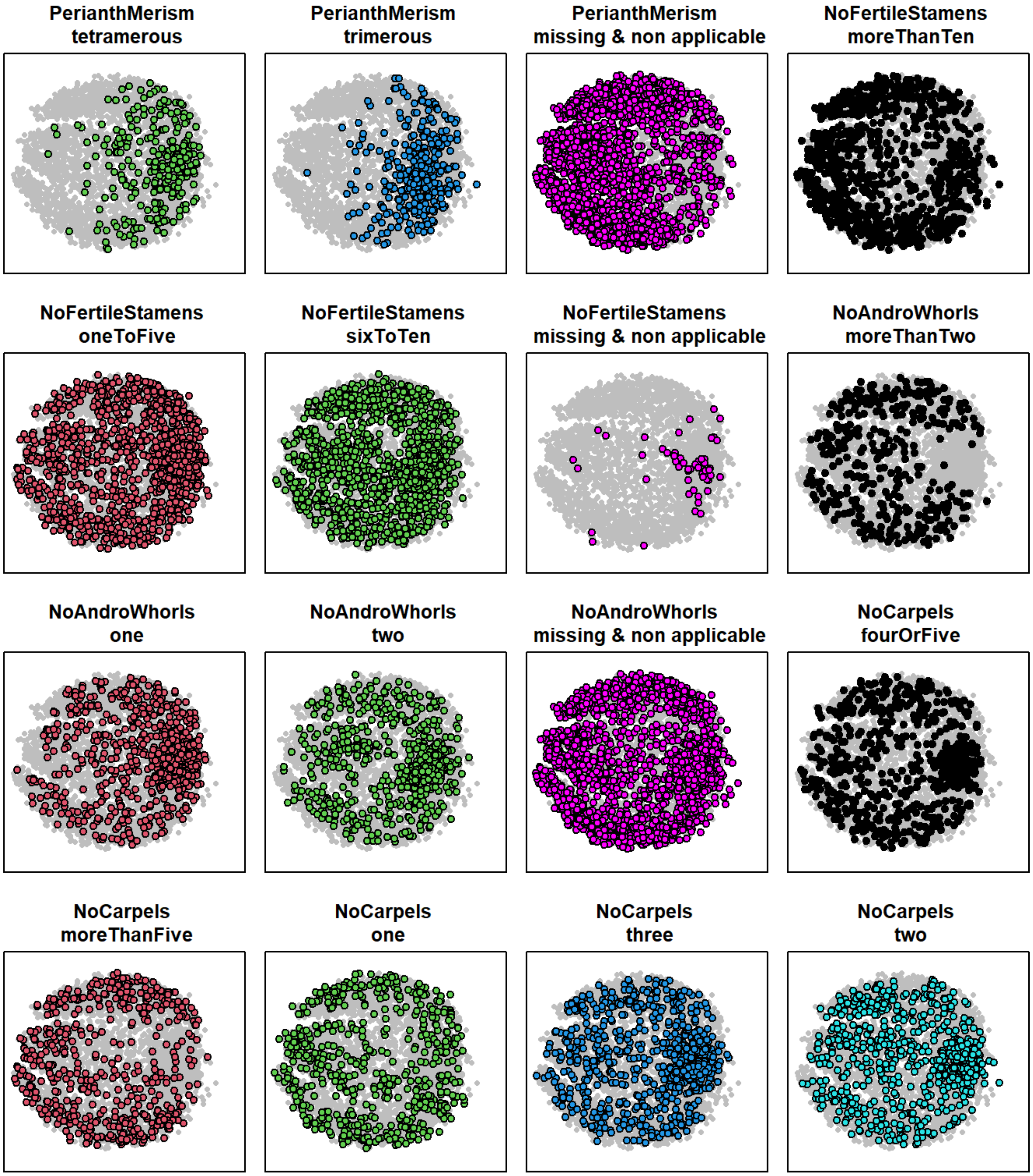
PerianthMerism tetramerous
PerianthMerism trimerous
PerianthMerism missing & non applicable
NoFertileStamens moreThanTen
NoFertileStamens oneToFive
NoFertileStamens sixToTen
NoFertileStamens missing & non applicable
NoAndroWhorls moreThanTwo
NoAndroWhorls one
NoAndroWhorls two
NoAndroWhorls missing & non applicable
NoCarpels fourOrFive
NoCarpels moreThanFive
NoCarpels one
NoCarpels three
NoCarpels two

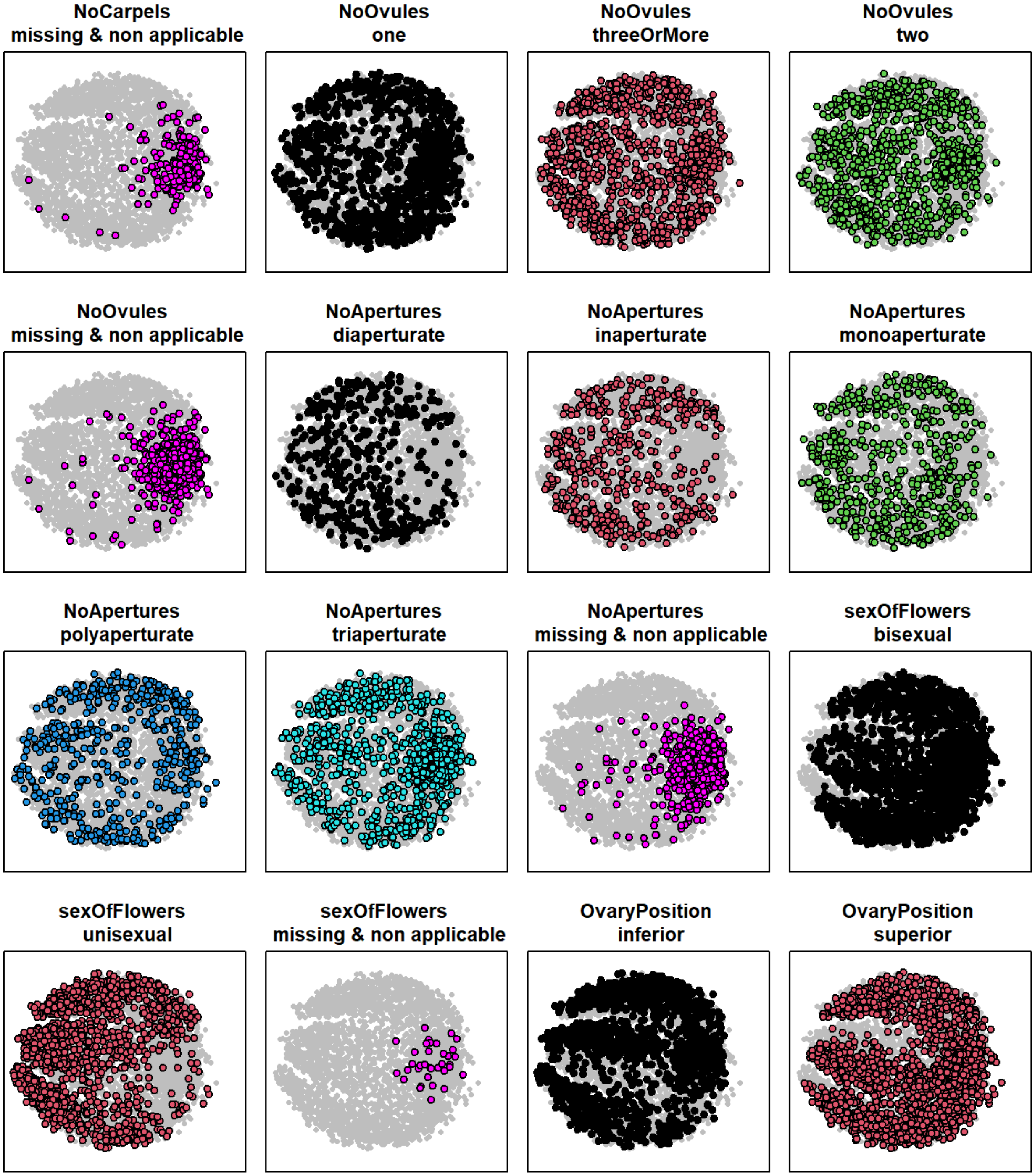
NoCarpels
missing & non applicable
NoOvules
one
NoOvules
threeOrMore
NoOvules
two
NoOvules
missing & non applicable
NoApertures
diaperturate
NoApertures
inaperturate
NoApertures
monoaperturate
NoApertures
polyaperturate
NoApertures
triaperturate
NoApertures
missing & non applicable
sexOfFlowers
bisexual
sexOfFlowers
unisexual
sexOfFlowers
missing & non applicable
OvaryPosition
inferior
OvaryPosition
superior

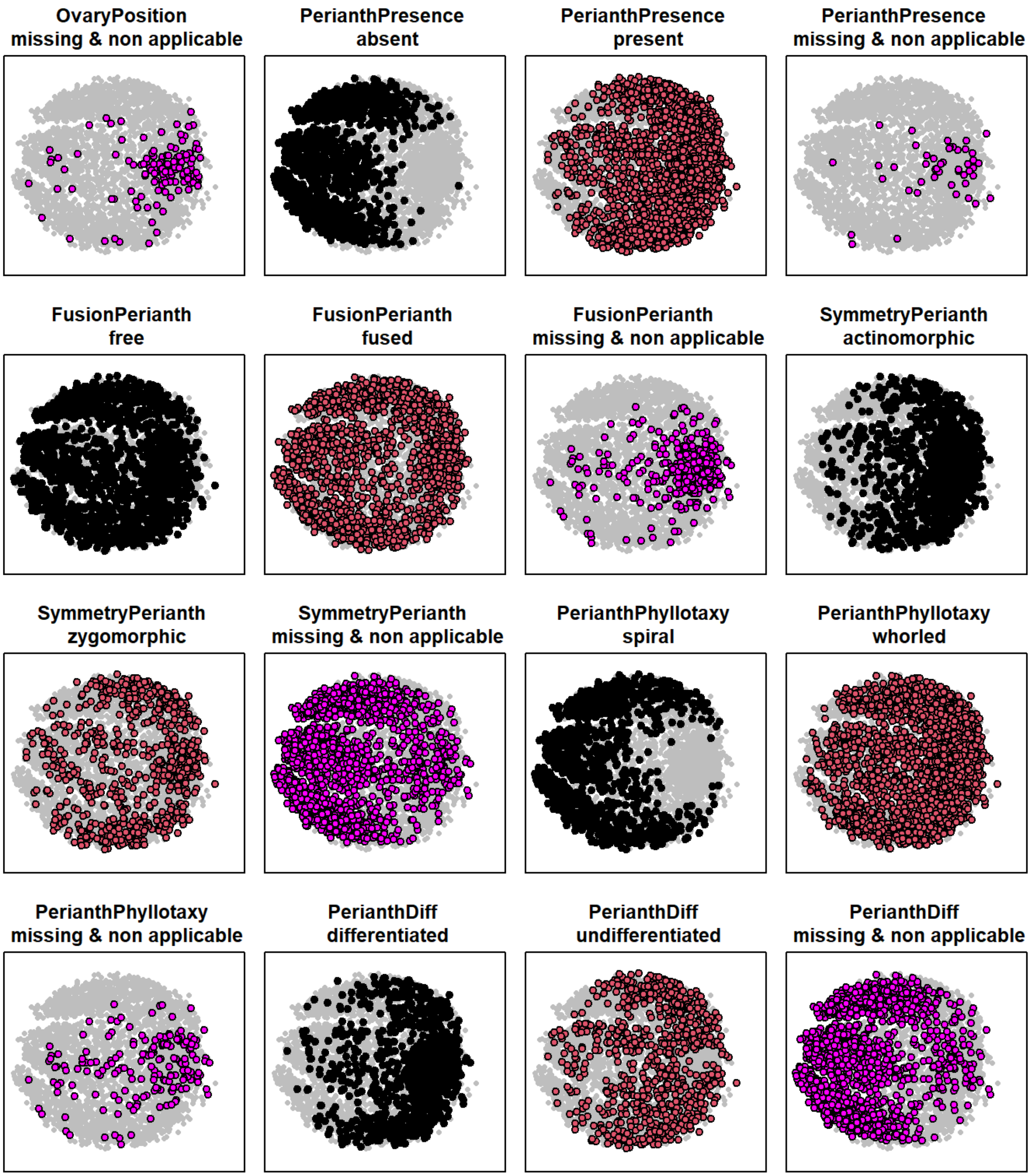
OvaryPosition
missing & non applicable
PerianthPresence
absent
PerianthPresence
present
PerianthPresence
missing & non applicable
FusionPerianth
free
FusionPerianth
fused
FusionPerianth
missing & non applicable
SymmetryPerianth
actinomorphic
SymmetryPerianth
zygomorphic
SymmetryPerianth
missing & non applicable
PerianthPhyllotaxy
spiral
PerianthPhyllotaxy
whorled
PerianthPhyllotaxy
missing & non applicable
PerianthDiff
differentiated
PerianthDiff
undifferentiated
PerianthDiff
missing & non applicable

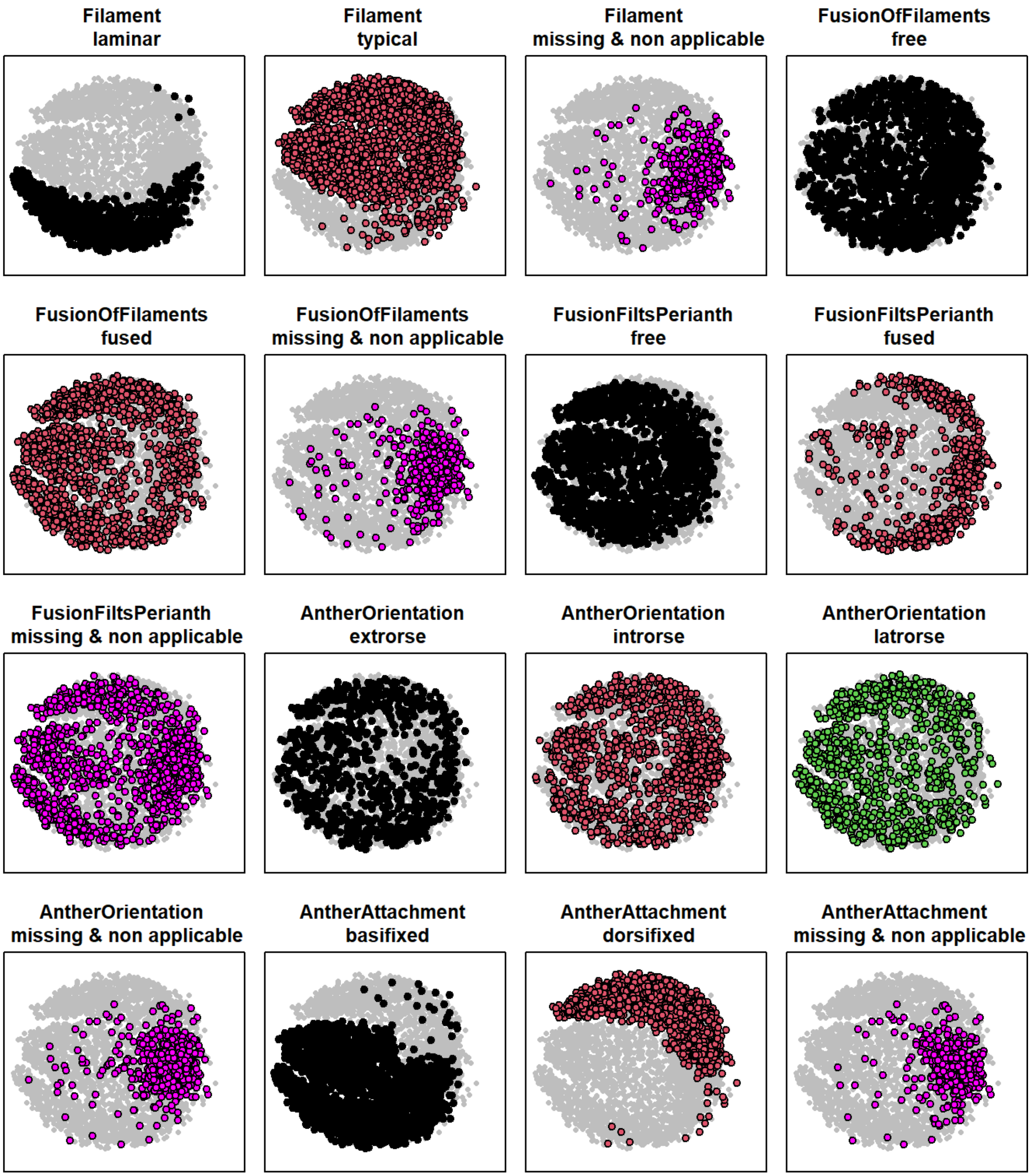
Filament
laminar
Filament
typical
Filament
missing & non applicable
FusionOfFilaments
free
FusionOfFilaments
fused
FusionOfFilaments
missing & non applicable
FusionFiltsPerianth
free
FusionFiltsPerianth
fused
FusionFiltsPerianth
missing & non applicable
AntherOrientation
extrorse
AntherOrientation
introrse
AntherOrientation
latrorse
AntherOrientation
missing & non applicable
AntherAttachment
basifixed
AntherAttachment
dorsifixed
AntherAttachment
missing & non applicable

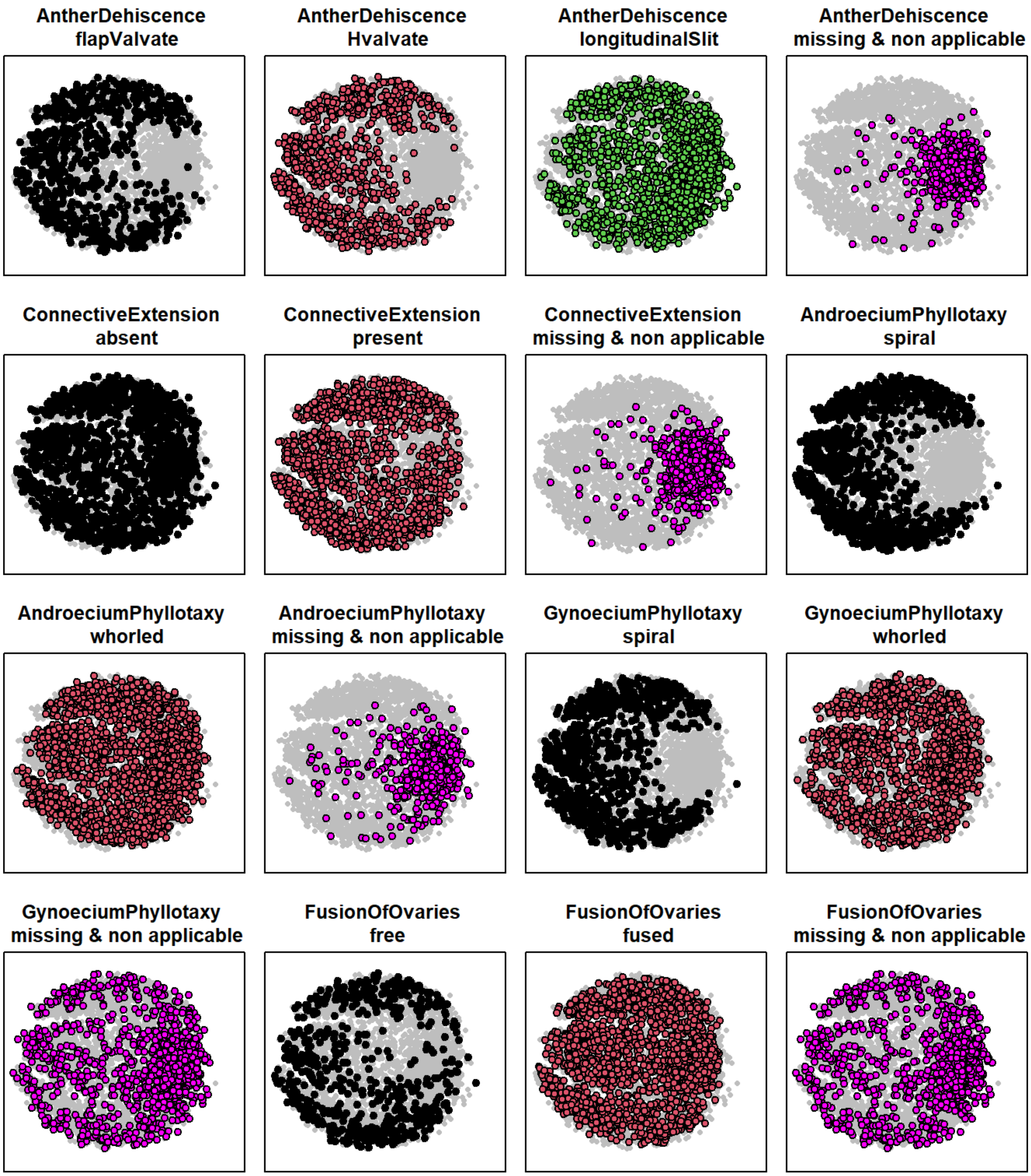
AntherDehiscence flapValvate
AntherDehiscence Hvalvate
AntherDehiscence longitudinalSlit
AntherDehiscence missing & non applicable
ConnectiveExtension absent
ConnectiveExtension present
ConnectiveExtension missing & non applicable
AndroeciumPhyllotaxy spiral
AndroeciumPhyllotaxy whorled
AndroeciumPhyllotaxy missing & non applicable
GynoeciumPhyllotaxy spiral
GynoeciumPhyllotaxy whorled
GynoeciumPhyllotaxy missing & non applicable
FusionOfOvaries free
FusionOfOvaries fused
FusionOfOvaries missing & non applicable

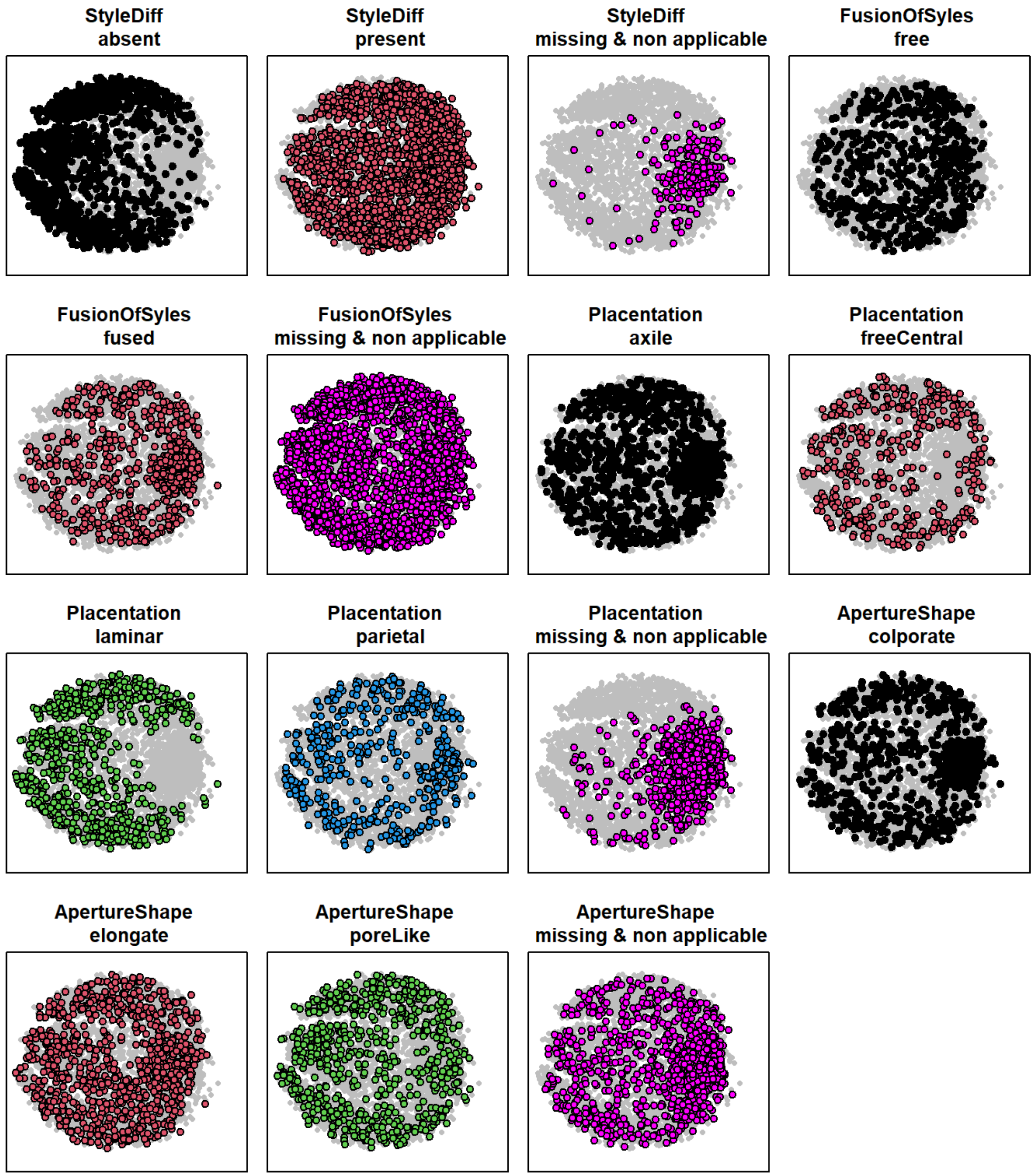
StyleDiff absent
StyleDiff present
StyleDiff missing & non applicable
FusionOfSyles free
FusionOfSyles fused
FusionOfSyles missing & non applicable
Placentation axile
Placentation freeCentral
Placentation laminar
Placentation parietal
Placentation missing & non applicable
ApertureShape colporate
ApertureShape elongate
ApertureShape poreLike
ApertureShape missing & non applicable

Fig. S4 Position of eccentric species in the morphospace ordination.

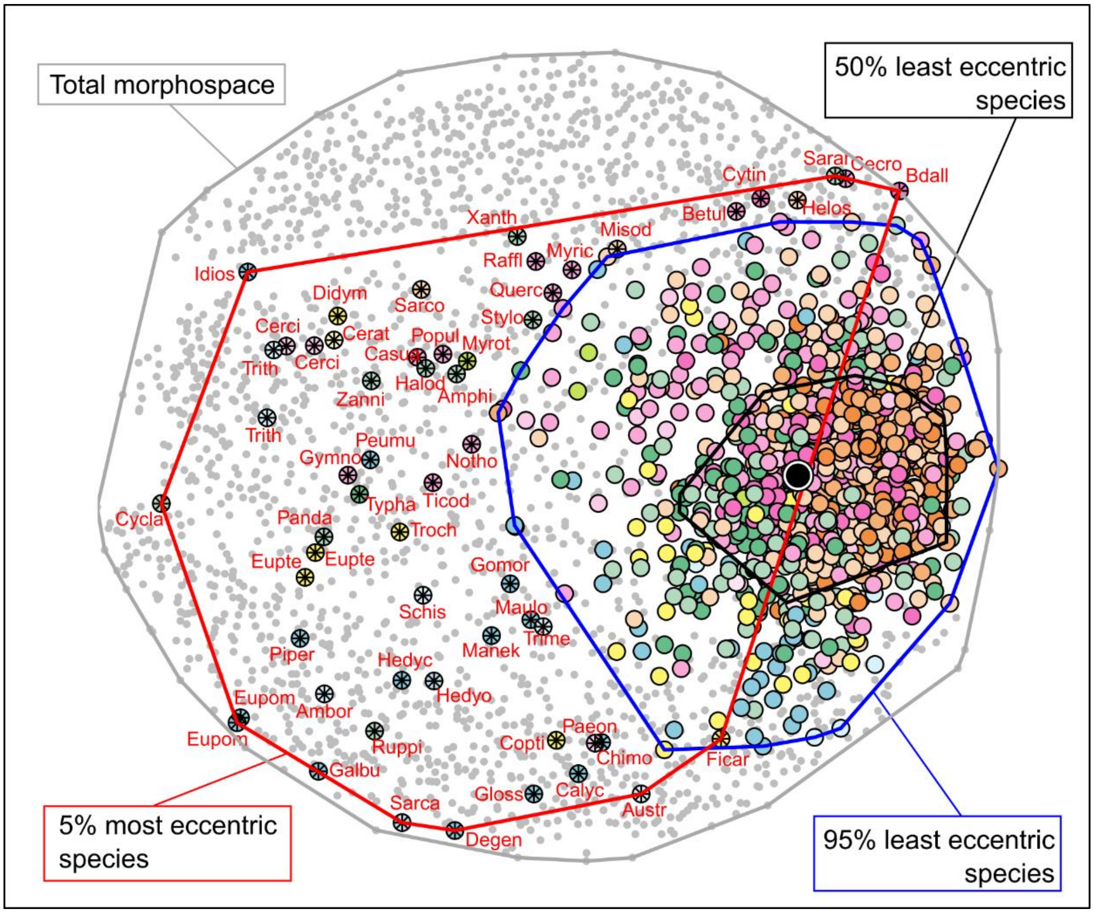

Morphospace ordination in which all morphologies (total morphospace) are surrounded by a gray convex hull, the 5% most eccentric living species by a red convex hull (this area represents 59.26% of the total morphospace area), the 95% (35.51% of the total morphospace area) least eccentric species by a blue convex hull, and the 50% (8.03% of the total morphospace area) least eccentric species by a black convex hull. The black dot indicates the position of centroid for living species. Gray dots = theoretical combinations; colored dots = living species; colored dots with a star: 5% most eccentric species (n = 58, listed below). nMDS = Fig.3.

1 . *Eupomatia bennettii*
2 . *Eupomatia laurina*
3 . *Cyclanthus bipartitus*
4 . *Galbulimima belgraveana*
5 . *Sarcandra chloranthoides*
6 . *Degeneria vitiensis*
7 . *Idiospermum australiense*
8 . *Amborella trichopoda*
9 . *Trithuria cowieana*
10 . *Euptelea polyandra*
11 . *Euptelea pleiosperma*
12 . *Trithuria submersa*
13 . *Ruppia megacarpa*
14 . *Cercidiphyllum magnificum*
15 . *Cercidiphyllum japonicum*
16 . *Hedycarya arborea*
17 . *Piper betle*
18 . *Didymeles perrieri*
19 . *Pandanus tectorius*
20 . *Ceratophyllum demersum*
21 . *Glossocalyx brevipes*
22 . *Sarcobatus vermiculatus*
23 . *Gymnostoma deplancheanum*
24 . *Peumus boldus*
25 . *Trochodendron aralioides*
26 . *Coptis chinensis*
27 . *Typha latifolia*
28 . *Zannichellia palustris*
29 . *Hedyosmum arborescens*
30 . *Cecropia insignis*
31 . *Rafflesia keithii*
32 . *Schisandra chinensis*
33 . *Calycanthus floridus*

34 . *Austrobaileya scandens*
35 . *Casuarina cunninghamiana*
36 . *Xanthosoma sagittifolium*
37 . *Sararanga sinuosa*
38 . *Paeonia californica*
39 . *Nothofagus cunninghamii*
40 . *Manekia sydowii*
41 . *Populus tremuloides*
42 . *Halodule wrightii*
43 . *Chimonanthus praecox*
44 . *Mauloutchia chapelieri*
45 . *Myrothamnus flabellifolia*
46 . *Bdallophytum americanum*
47 . *Amphibolis griffithii*
48 . *Gomortega keule*
49 . *Ticodendron incognitum*
50 . *Misodendrum linearifolium*
51 . *Quercus rubra*
52 . *Helosis cayennensis*
53 . *Cytinus hypocistis*
54 . *Trimenia moorei*
55 . *Ficaria verna*
56 . *Betula ermanii*
57 . *Stylochaeton bogneri*
58 . *Myrica cerifera*

Fig. S7 Distribution of character states in living Angiosperms

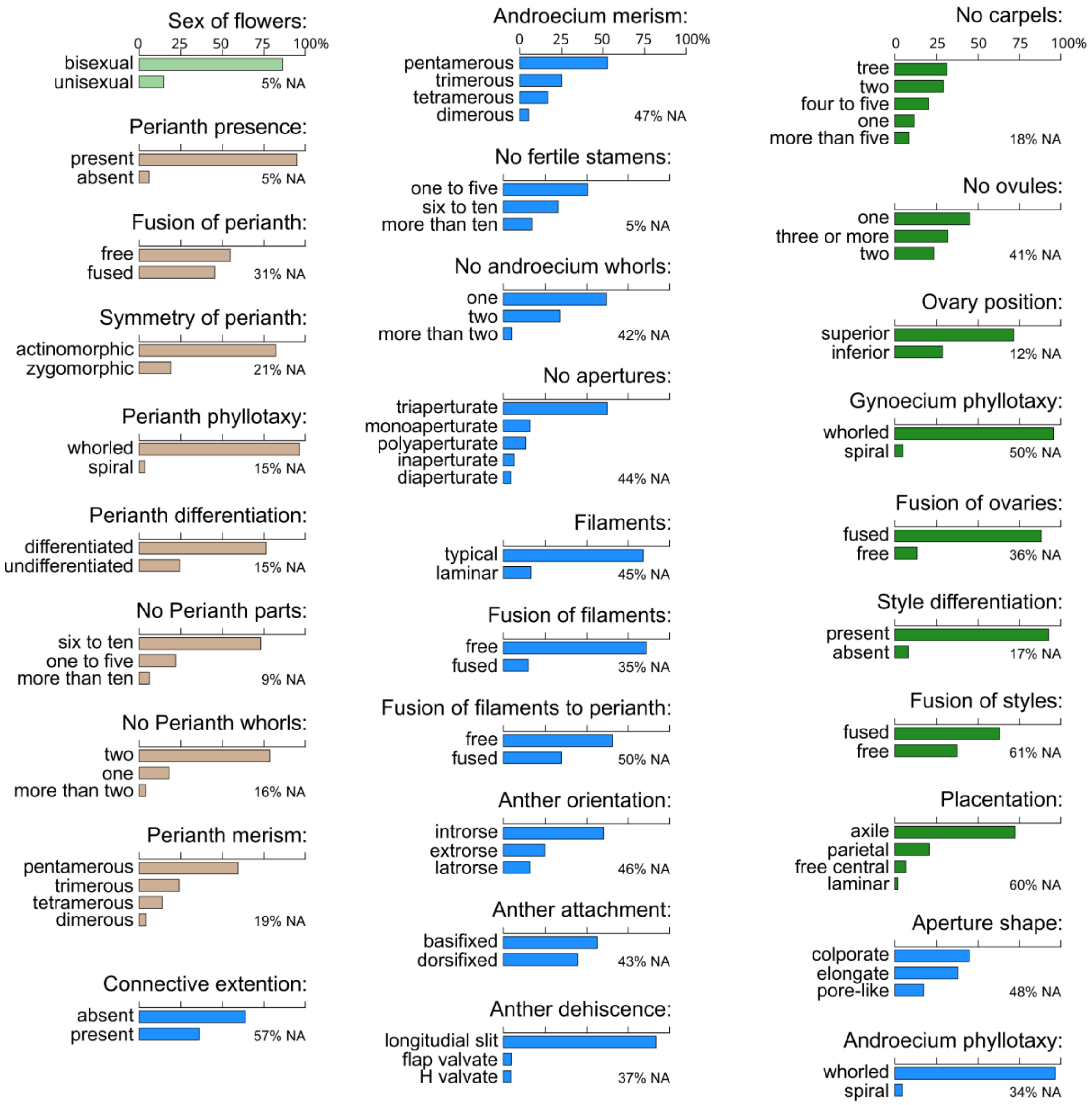

Frequency of the study character states in the 1201 sampled living angiosperms. The percentage of missing data in the dataset is given for each character on the bottom right of each graph.

Fig. S8 Rarefied floral disparity (*meanD* and *R*) for living angiosperms

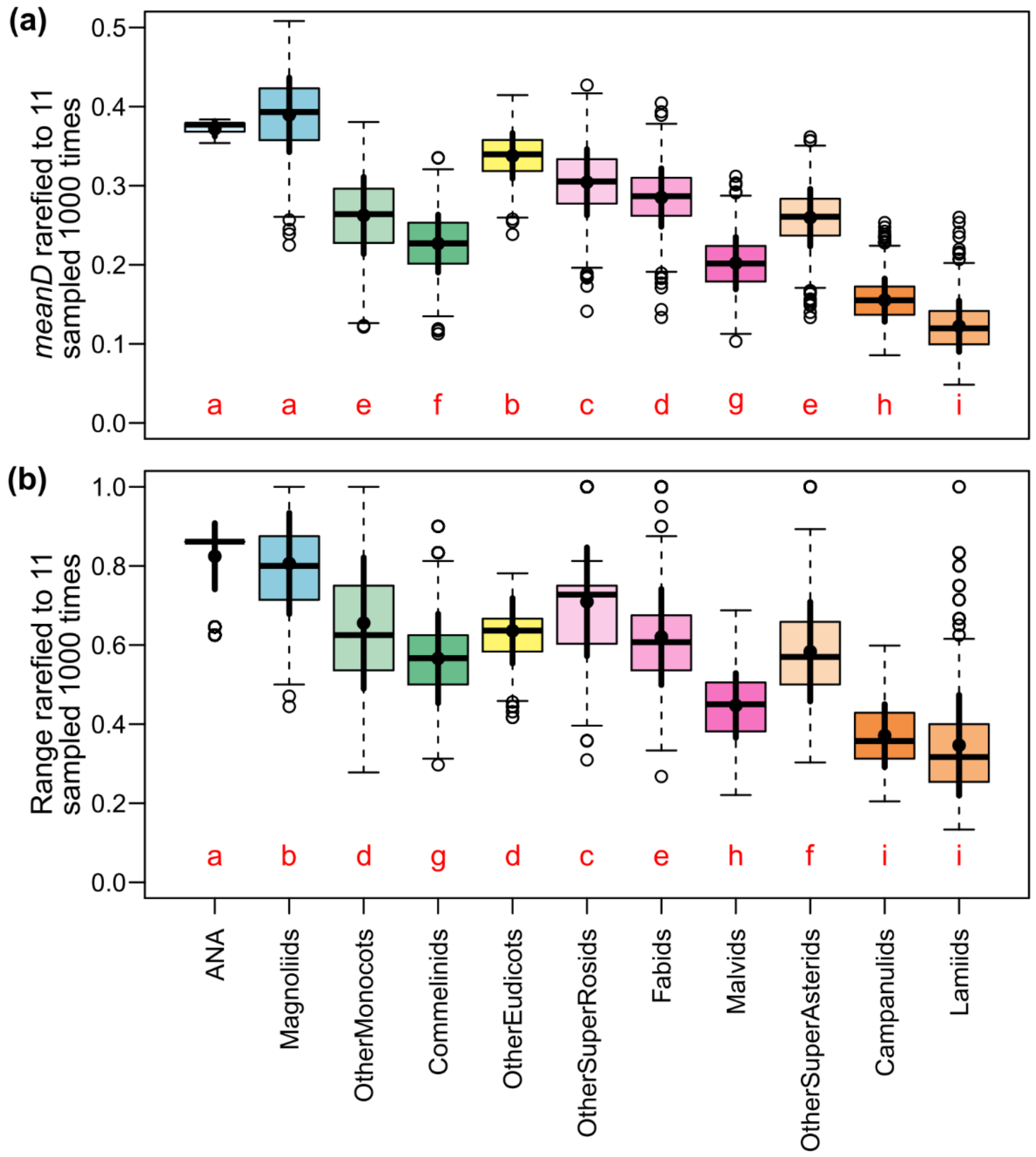

Rarefied *meanD* and rarefied *R* for eleven angiosperm clades and grades. Groups with the same red letter are not significantly different. In **(a)**: Kruskal-Wallis chi-squared = 9211.5, df = 10, p-value < 2.2e-16; in **(b)**: chi-squared = 7069.1, df = 10, p-value < 2.2e-16. *Post hoc* tests results are indicated by the red letters, groups with different letters significantly differ.

Fig. S9 Position of additional living angiosperm clades in the floral morphospace (Fig. 3)

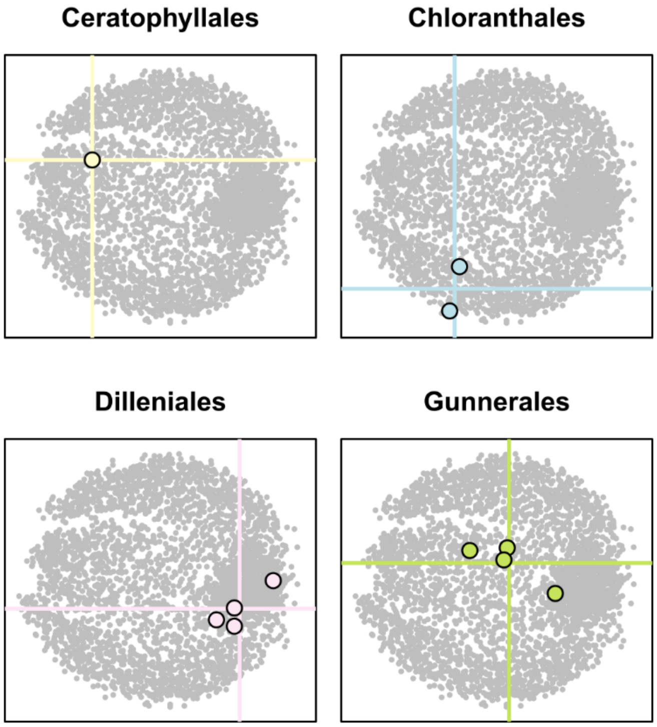

The orders Chloranthales, Ceratophyllales, Gunnerales, and Dilleniales were excluded from the statistical analyses as they contained only two to four species in our sampling and because they cannot be assigned to any previously specified group. They were anyway included in the morphospace presented in Fig. 3.

Fig. S10 Correlation between sample size and groups sizes

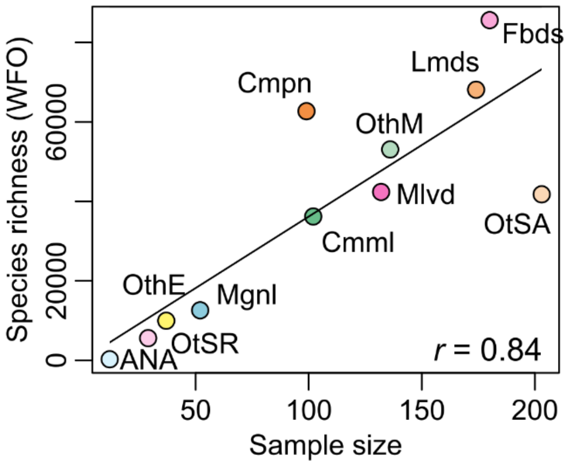

Correlations between species richness and sample size. Black lines: linear regressions. *r* = Pearson's product moment correlation coefficient. ANA = ANA grade; Mgnl = magnoliids; OthM = the other monocot grade; Cmml = commelinids; OthE = the other eudicot grade, OtSA = the other superasterids grade; Lmds = lamiids; Cmpn = campanulids; OtSR = the other superrosidae grade; Fbds = fabiids; Mlvd = malviids.

Fig. S11 Phylogenetic distribution of species in the four time bins studied and for eccentric species

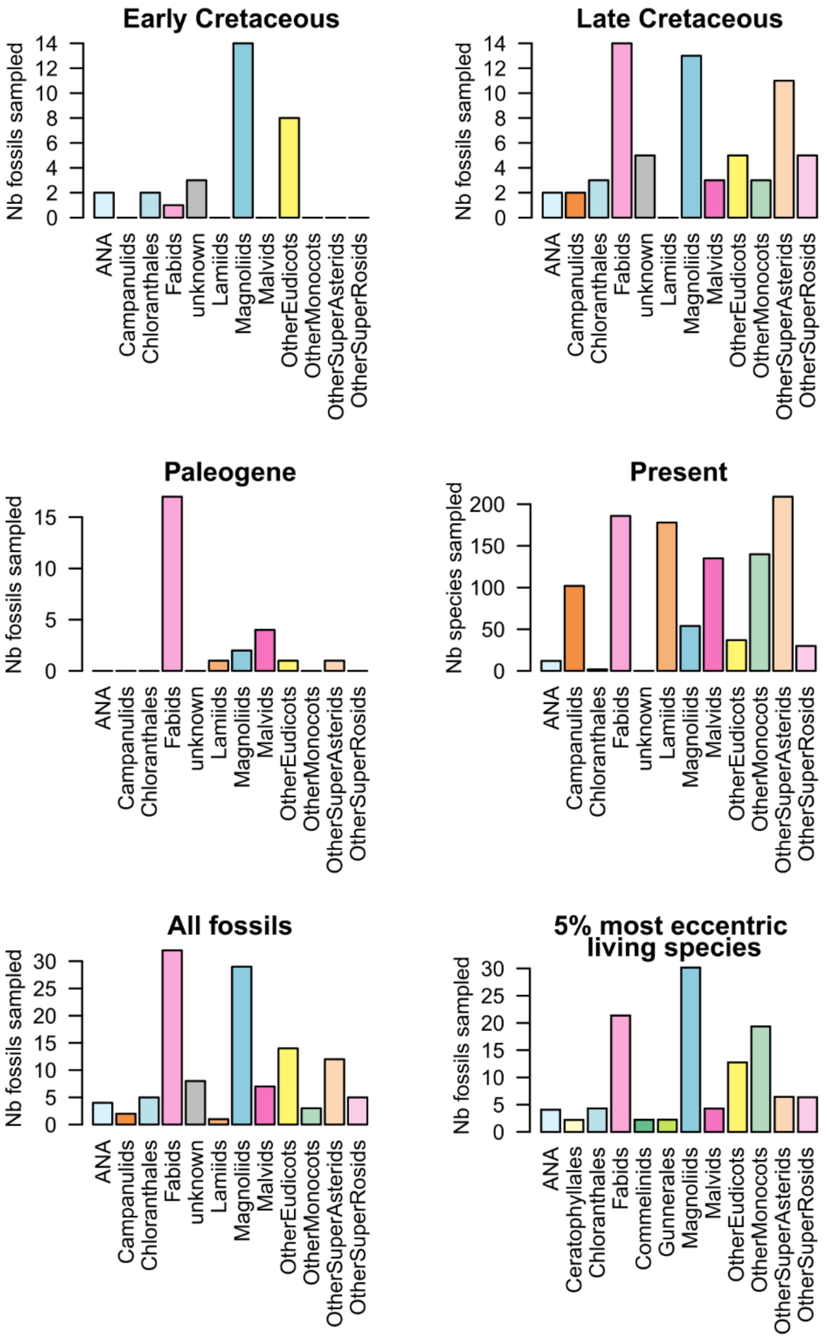

Number of species belonging to each of the study 11 groups, for each stratigraphic time bin analyzed, for all fossils pooled together and for the 5% (n = 58) most eccentric living species. The two fossils from the Neogene (not shown here) were affiliated to the Malvids and the Fabids.

Methods S1 List of non-applicable and impossible combinations.

Character state combinations that are non-applicable (NA) mostly refer to the definition of organs when they are absent (for example differentiation of perianth when there is no perianth), or that are impossible per definition (inferior ovary without union of carpels).

1. Perianth absence -> perianth merism is NA
2. Perianth absence -> perianth differentiation is NA
3. Perianth absence -> perianth fusion is NA
4. Perianth absence -> number of perianth whorls is NA
5. Perianth absence -> number of perianth parts is NA
6. Perianth absence -> fusion of filaments to perianth is NA

7. Spiral androecium phyllotaxy -> androecium merism is NA
8. Spiral androecium phyllotaxy -> number of androecium whorls is NA

9. Spiral perianth phyllotaxy -> perianth merism is NA
10. Spiral perianth phyllotaxy -> number of perianth whorls is NA

11. One ovary -> gynoecium phyllotaxy is NA
12. One ovary -> fusion of ovaries is NA
13. One ovary -> fusion of styles is NA

14. Style undifferentiated -> fusion of styles is NA

15. Pollen inaperturate -> aperture shape is NA

16. Free ovaries + parietal placentation is impossible
17. Free ovaries + free central placentation is impossible
18. Inferior ovary + free carpels is impossible

Methods S2 Calculation of the mean character difference

Let us have two taxa *A* and *B* described from a dataset with *N* morphological characters. For a character *i*, the difference d_{ABi} between *A* and *B* was calculated in different ways depending on the type of character:

- for ordered categorical characters (e.g. number of perianth parts), d_{ABi} was calculated as the number of steps between the values of the character for *A* and *B*, divided by the maximum possible step difference for the character in the whole dataset*.

- for binary and unordered categorical characters (e.g. sex of flowers), d_{ABi} took the value {1} if *A* and *B* shared the same state, {0} if not;

- if the value of a character was missing for *A* or/and *B*, this character was removed from the calculation of *D*. *N* was thus reduced to the number N_{AB} of characters with no missing data for *A* or *B*.

The mean character difference D_{AB}between taxa *A* and *B* was finally computed as:

$$D_{AB} = \sum [d_{ABii} / N_{AB}].$$

D was calculated for each pair of taxa to generate the dissimilarity matrix.

Note that some taxa were removed while computing the distance matrix if a pairwise distance could not be calculated between these taxa and too many other taxa (for example, because there were too many missing data or not enough overlapping data).

* for that reason, all values of *D* in the distance matrix depend on the data included in the analysis.

Table S1 Divergence times estimates for the 15 ASR

Node	RC_complete Mya	CC_complete Mya	Period
Angiospermae	195,76	154,12	Jurassic
Mesangiospermae	163,79	152,34	Jurassic
Magnoliidae	153,66	146,46	Jurassic
Monocotyledoneae	151,12	146,46	Early Cretaceous-Jurassic
Commelinidae	133,38	129,13	Early Cretaceous
Eudicotyledoneae	151,94	148,07	Early Cretaceous
Pentapetalae	146,86	143,36	Early Cretaceous
Superasteridae	143,87	140,23	Early Cretaceous
Asteridae	136,73	133,85	Early Cretaceous
Campanulidae	124,39	122,05	Early Cretaceous
Lamidae	125,67	124,23	Early Cretaceous
Superrosidae	143,82	139,99	Early Cretaceous
Rosidae	142,54	138,66	Early Cretaceous
Fabidae	138,06	133,74	Early Cretaceous
Malvidae	138,05	134,01	Early Cretaceous

Divergence times estimates (averages) from Ramírez-Barahora et al. (2020) *Nat Ecol Evol* for the 15 ancestral state reconstructions of angiosperms used in this study. RC = relaxed calibration, CC = constrained calibration. Mya = million years ago.

Table S2 Disparity (*meanD*) for the study angiosperm group

Group	*meanD*	SD	n
ANA grade	0,3847	0,1425	12
campanulids	0,1573	0,0941	99
commelinids	0,2284	0,133	102
fabids	0,2837	0,1346	180
lamiids	0,1197	0,0883	174
magnoliids	0,3836	0,1636	52
malvids	0,2029	0,1034	132
other super asterid grade	0,2517	0,1237	203
other eudicot grade	0,3368	0,1245	37
other monocot grade	0,2578	0,1562	136
other super rosid grade	0,2949	0,1493	29

SD = standard deviation, n = sample size after computing the distance matrix.
